# Correction: Increase of circulating IGFBP-4 following genotoxic stress and its implication for senescence

**DOI:** 10.7554/eLife.80871

**Published:** 2022-07-01

**Authors:** Nicola Alessio, Tiziana Squillaro, Giovanni Di Bernardo, Giovanni Galano, Roberto De Rosa, Mariarosa AB Melone, Gianfranco Peluso, Umberto Galderisi

**Keywords:** Human, Mouse

Alessio N, Squillaro T, Di Bernardo G, Galano G, De Rosa R, Melone MAB, Peluso G, Galderisi U. 2020. Increase of circulating IGFBP-4 following genotoxic stress and its implication for senescence. eLife **9**:e54523. doi: 10.7554/eLife.54523.

Published 30 March 2020

We have been made aware through PubPeer of a mistake in Figure 1C. Specifically, it was pointed out that the BM-MSC 144 hr post treatment panel from Figure 1C overlaps with one of the human MSC panels from Figure 2 of our 2017 Neoplasia paper (Allesio et al., 2017a).

These micrographs are representative images of senescence detection, as we state in the Material and methods. The procedure for counting senescent cells is the following: the percentage of positive cells is calculated by the number of cells that expressed the specific marker stain out of at least 500 cells in different microscope fields. The microscope magnification was 100–200 X. In this condition, we can detect roughly 40/50 cells per field. This means that for every count we had to analyze at least 10 different microscopic fields.

In this context, some pictures are reported as representative images. Unfortunately, we made mistakes in the figure preparation for this manuscript and in our 2017 Neoplasia paper, and mistakenly used the same images twice.

We provide the new Figure 1, in which the representative images in panel c and h have been replaced. The original image data (gels and micrographs) and the spreadsheets used to generate all plots have now been added to the published article as source data for each figure.

The changes do not affect the results or conclusions of the original paper. We apologize for the mistake and the confusion this may have caused.

Corrected Figure 1:

**Figure 1:**
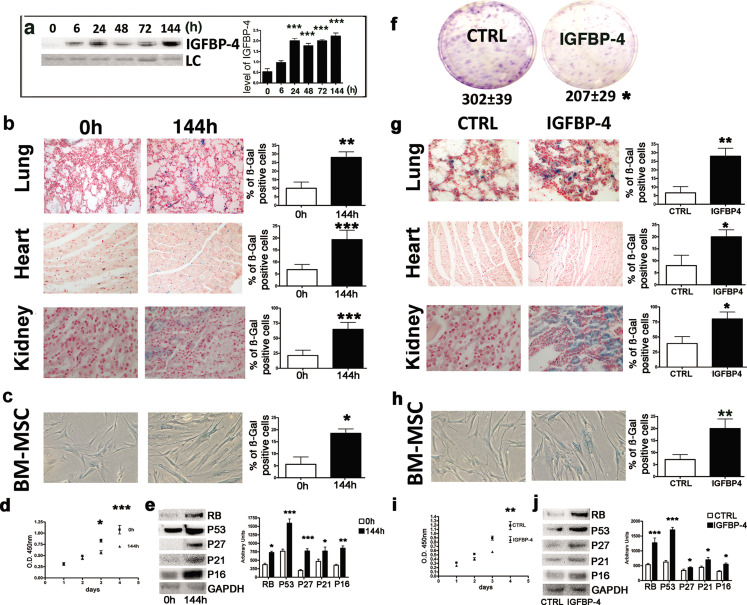


The original Figure 1 is shown here for reference:

**Figure 2:**
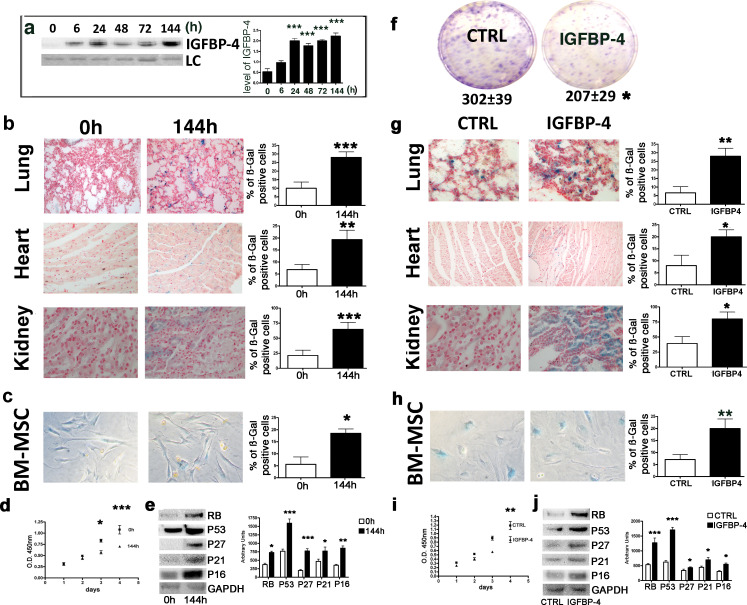


The article has been corrected accordingly.

Editors’ note: The authors contacted eLife on 19 March, 2022 requesting a Correction to address the issue with the duplicated images as reported on PubPeer. On 30 March, 2022 we asked the authors to provide the original image data for the two affected images, and they provided a dataset containing the images that have been used for the corrected version of the figure. On 21 April, 2022 we requested that the authors provide all the original image data for all the gels and micrographs for auditing purposes, which the authors provided and have been checked systematically. All original images corresponding to the panels shown in all figures, as well as the spreadsheets used to generate all plots are available as source data in the corrected manuscript.

