# Supplementary figures and images for: Increase of circulating IGFBP-4 following genotoxic stress and its implication for senescence
Source: eLife. 2020 Mar 30;9:e54523. doi: 10.7554/eLife.54523 (PMC7136022; doi:10.7554/eLife.54523)

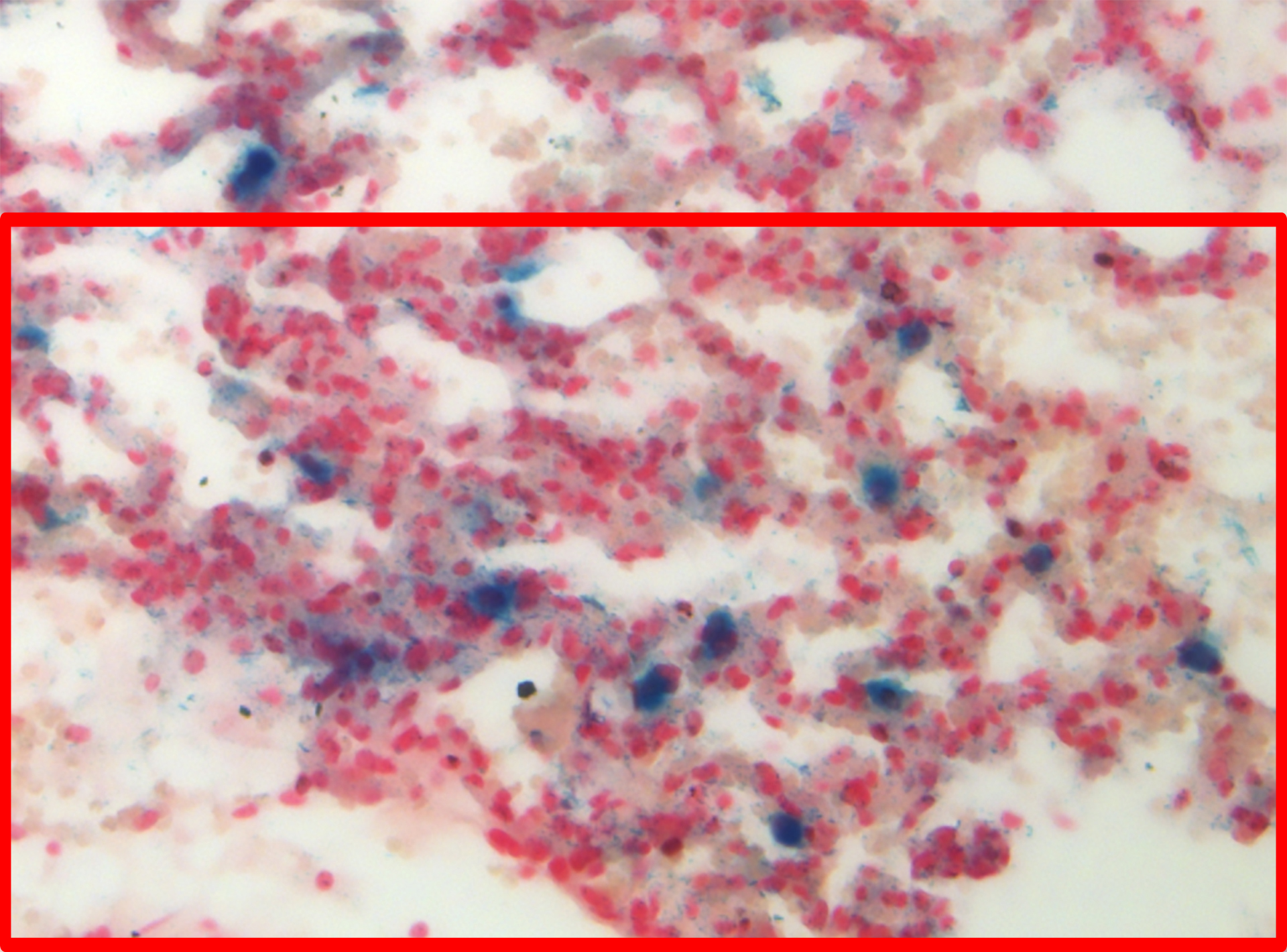

Supplement: Figure 1—source data 1. [file elife-54523-fig1-data1.zip › Figure 1/g/Lung IGFBP4.jpg]

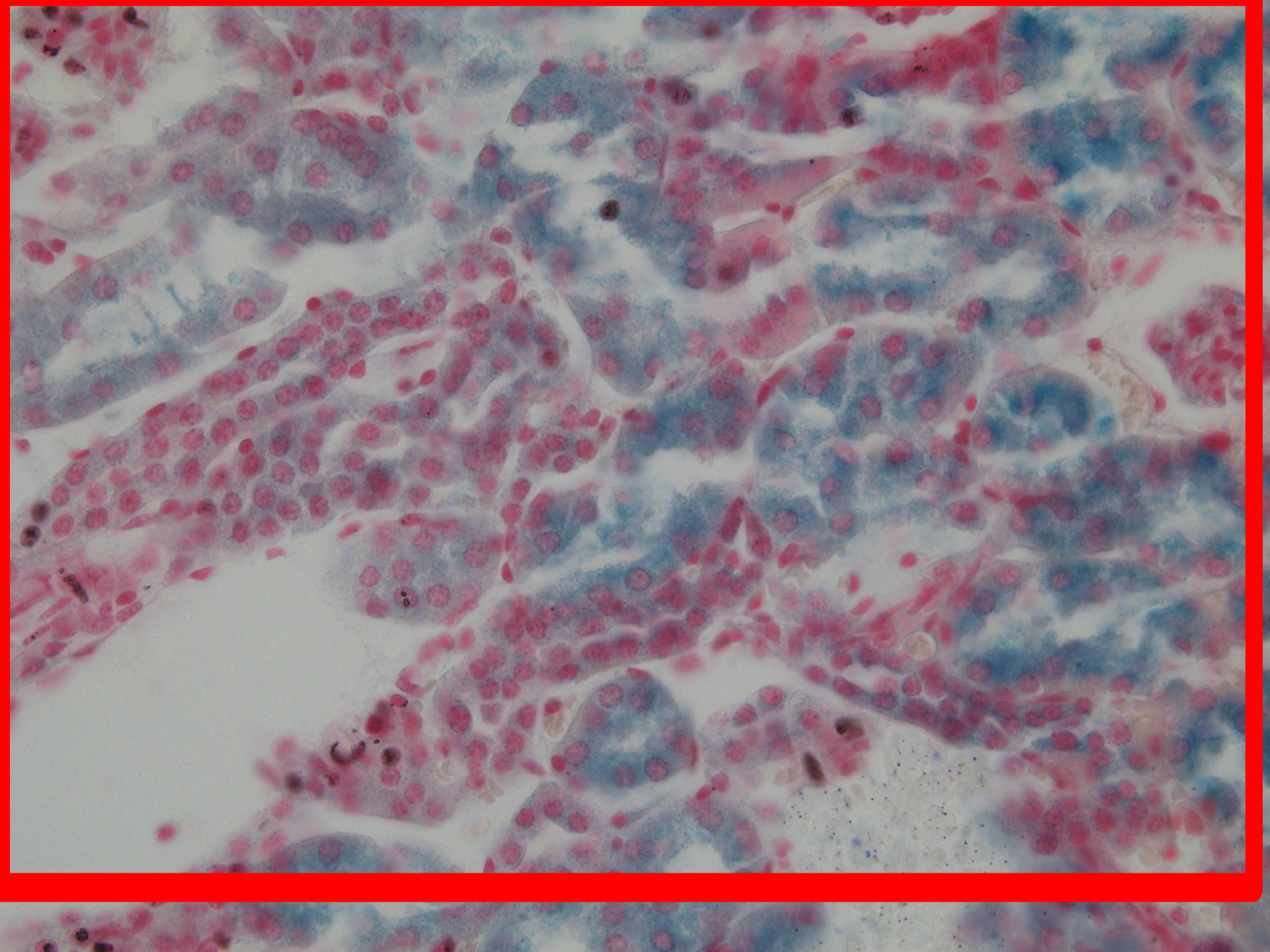

Supplement: Figure 1—source data 1. [file elife-54523-fig1-data1.zip › Figure 1/g/kidney IGFBP4.jpg]

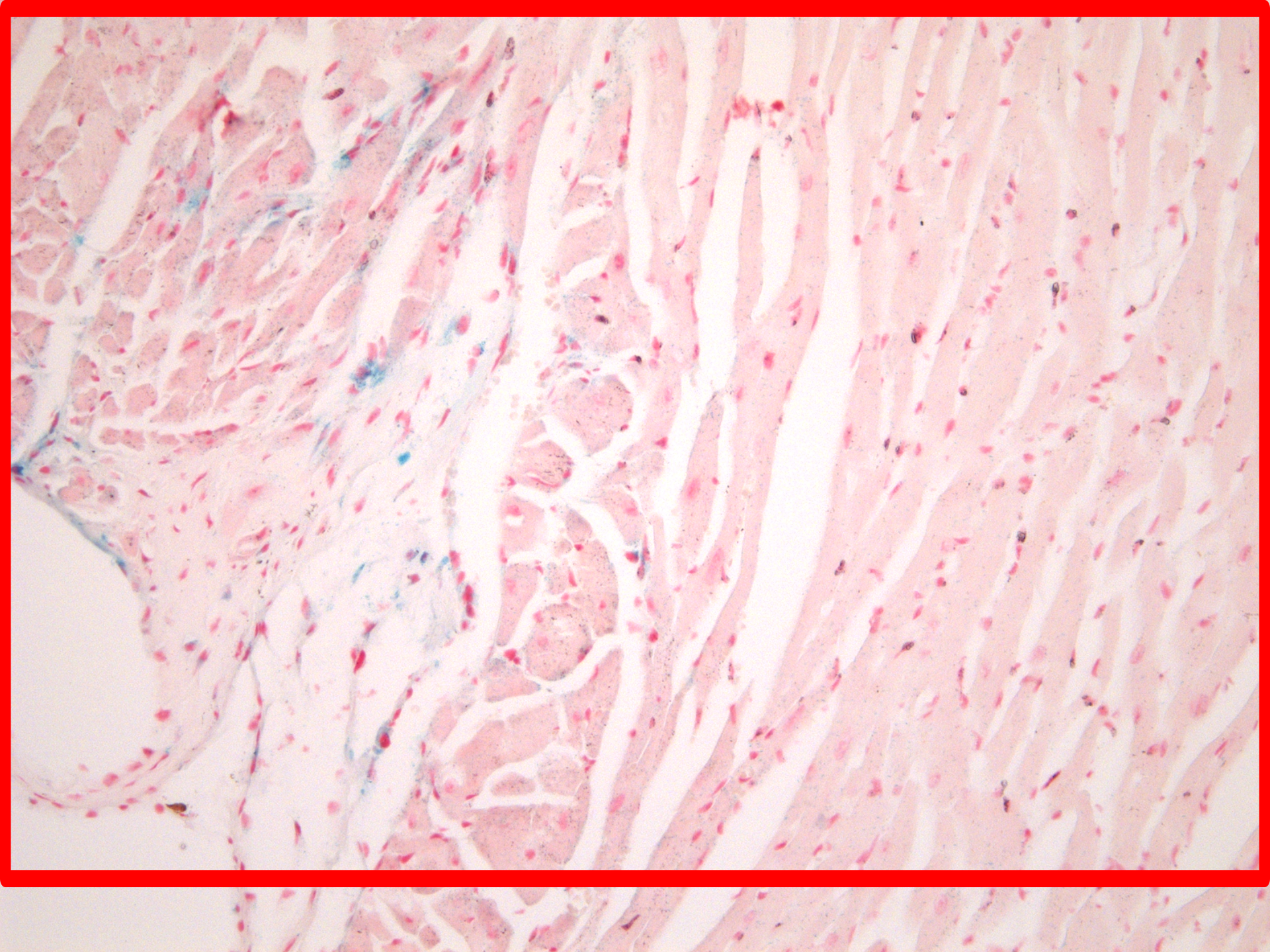

Supplement: Figure 1—source data 1. [file elife-54523-fig1-data1.zip › Figure 1/g/heart IGFBP4.tif]

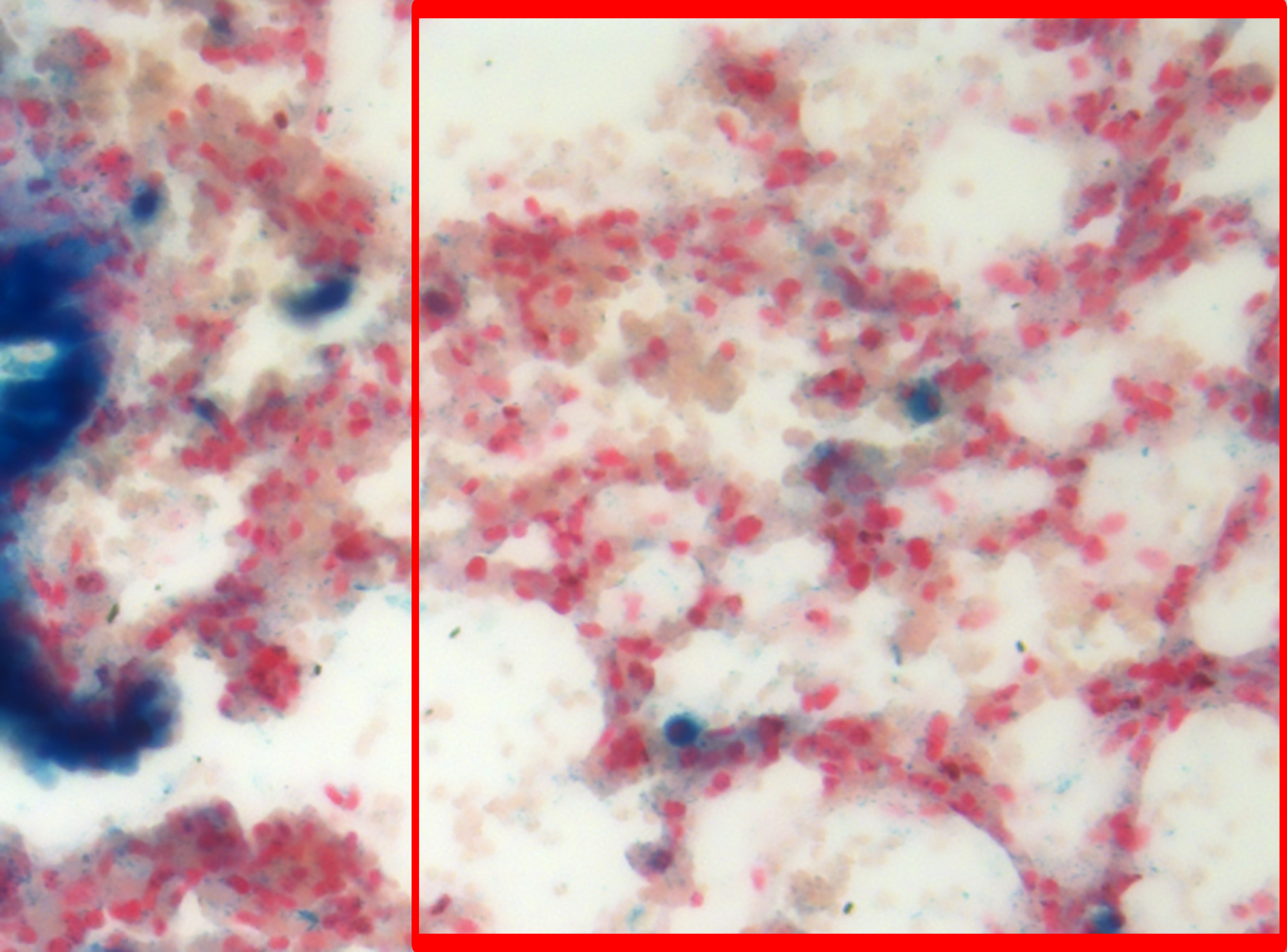

Supplement: Figure 1—source data 1. [file elife-54523-fig1-data1.zip › Figure 1/g/Lung CTRL.jpg]

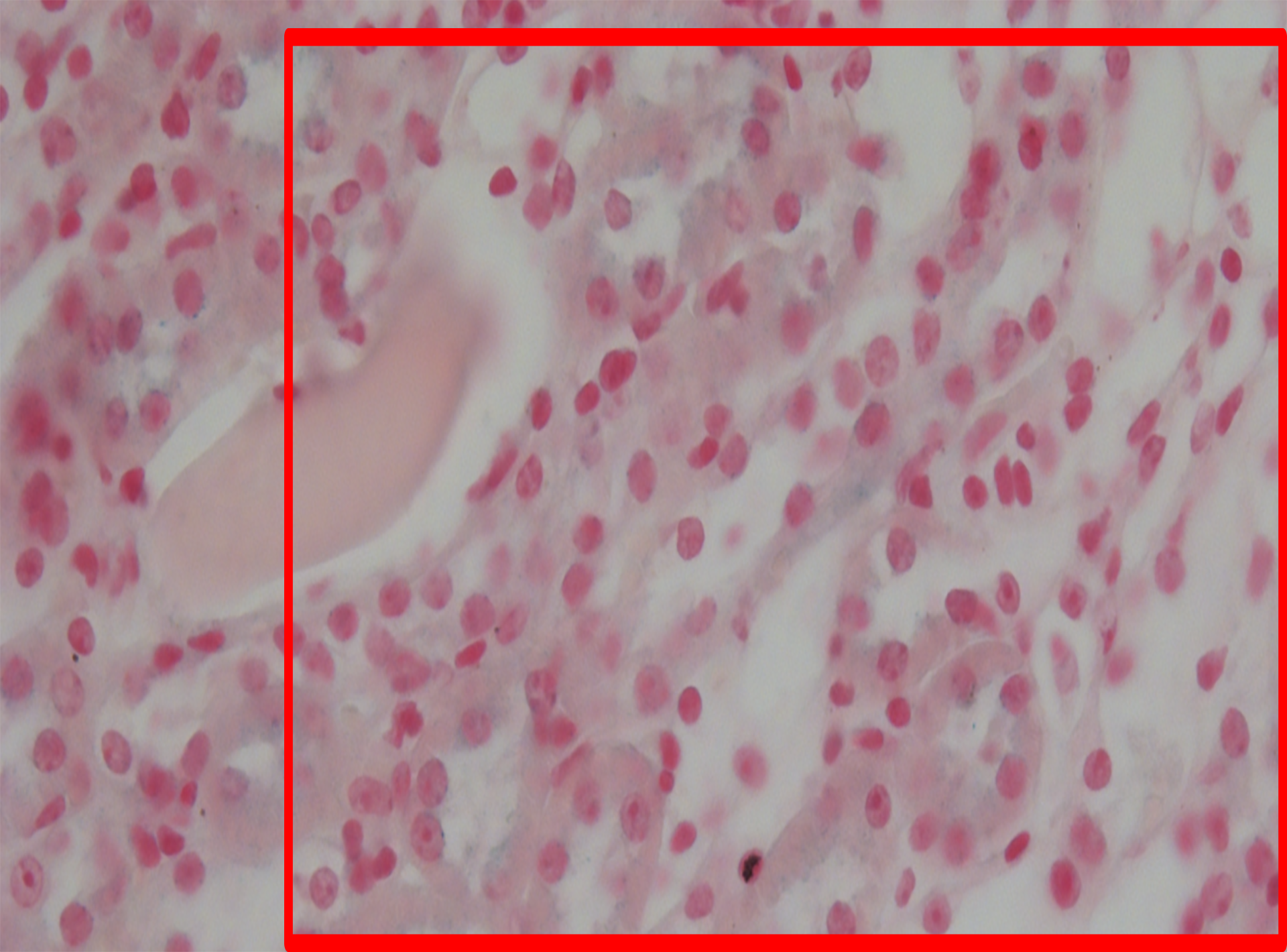

Supplement: Figure 1—source data 1. [file elife-54523-fig1-data1.zip › Figure 1/g/Kidney CTRL.tif]

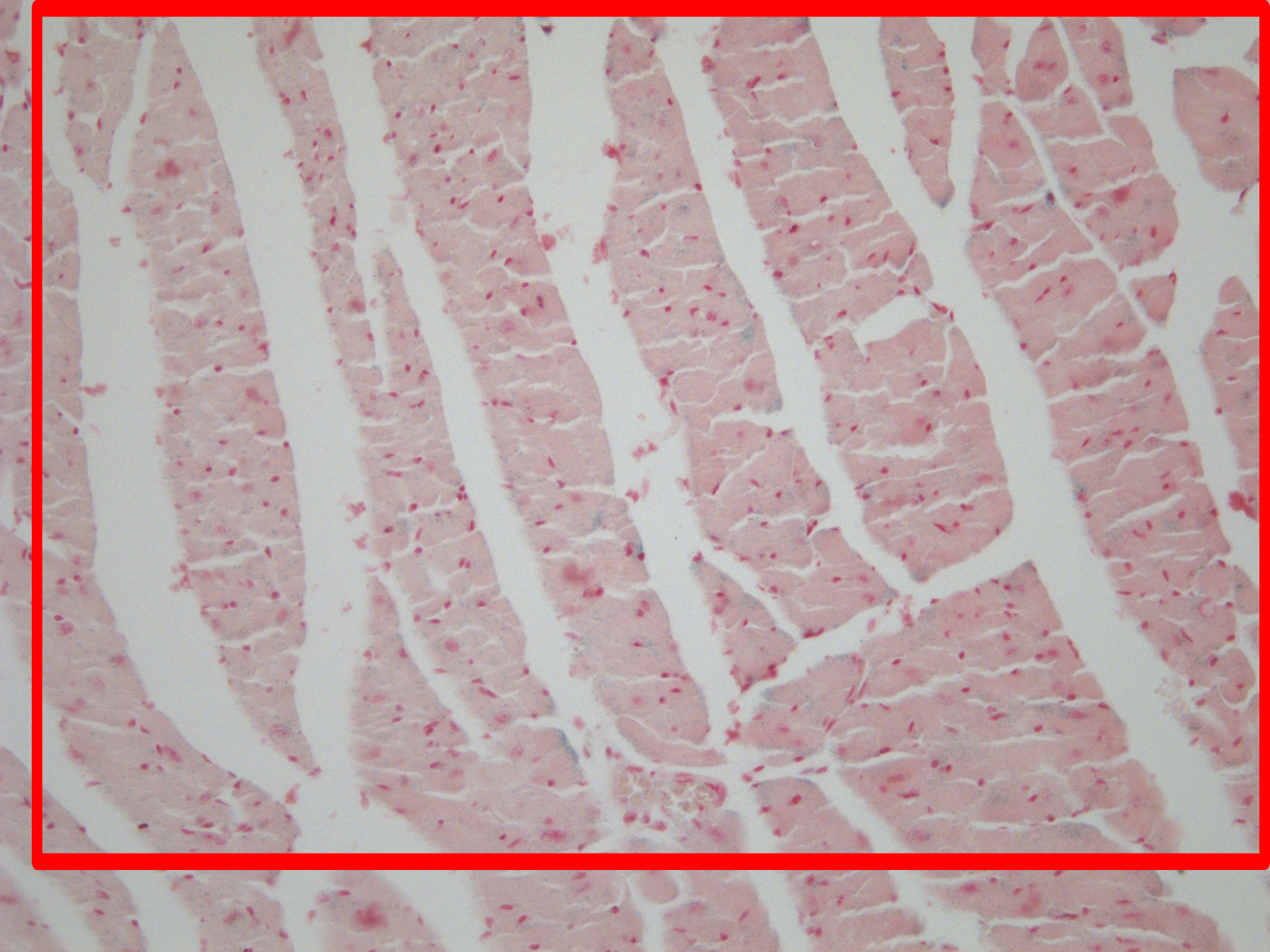

Supplement: Figure 1—source data 1. [file elife-54523-fig1-data1.zip › Figure 1/g/heart CTRL.tif]

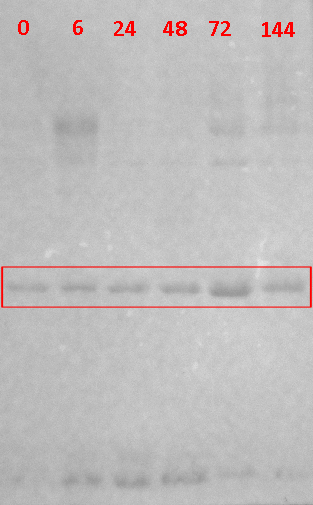

Supplement: Figure 1—source data 1. [file elife-54523-fig1-data1.zip › Figure 1/a/A LC.jpg]

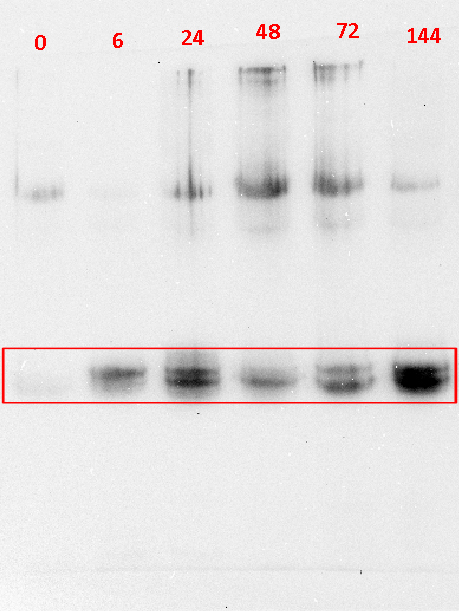

Supplement: Figure 1—source data 1. [file elife-54523-fig1-data1.zip › Figure 1/a/A WB IGFBP4.jpg]

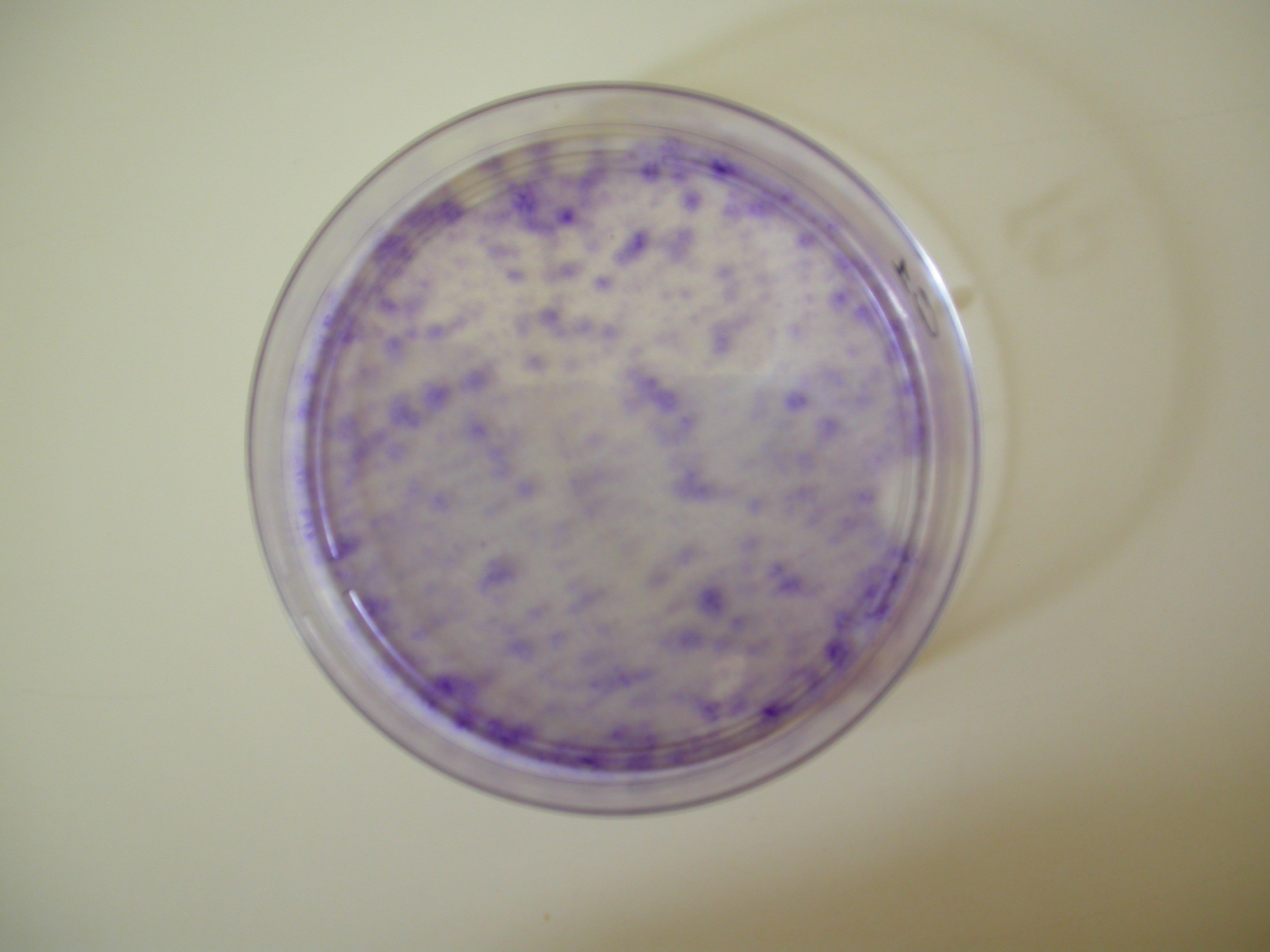

Supplement: Figure 1—source data 1. [file elife-54523-fig1-data1.zip › Figure 1/f/CTRL.JPG]

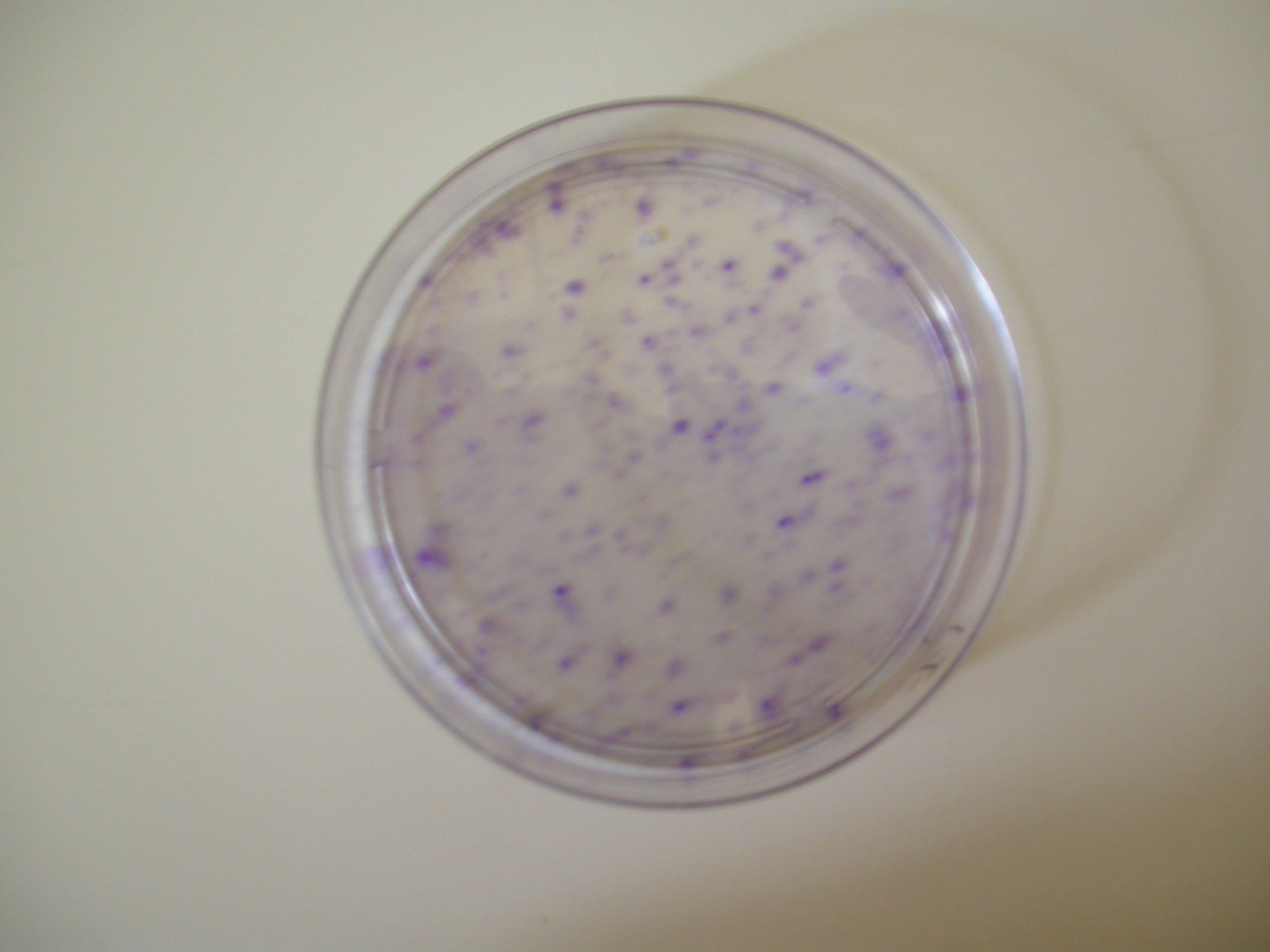

Supplement: Figure 1—source data 1. [file elife-54523-fig1-data1.zip › Figure 1/f/IGFBP4 .JPG]

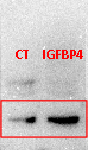

Supplement: Figure 1—source data 1. [file elife-54523-fig1-data1.zip › Figure 1/j/2.P53.jpg]

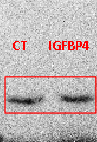

Supplement: Figure 1—source data 1. [file elife-54523-fig1-data1.zip › Figure 1/j/6.GAPDH.jpg]

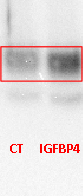

Supplement: Figure 1—source data 1. [file elife-54523-fig1-data1.zip › Figure 1/j/5.P16.jpg]

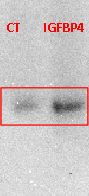

Supplement: Figure 1—source data 1. [file elife-54523-fig1-data1.zip › Figure 1/j/3.P27.jpg]

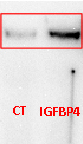

Supplement: Figure 1—source data 1. [file elife-54523-fig1-data1.zip › Figure 1/j/1.RB.jpg]

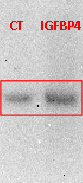

Supplement: Figure 1—source data 1. [file elife-54523-fig1-data1.zip › Figure 1/j/4.P21.jpg]

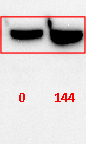

Supplement: Figure 1—source data 1. [file elife-54523-fig1-data1.zip › Figure 1/e/2.P53.jpg]

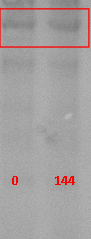

Supplement: Figure 1—source data 1. [file elife-54523-fig1-data1.zip › Figure 1/e/6.GAPDH.jpg]

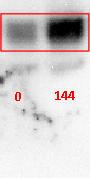

Supplement: Figure 1—source data 1. [file elife-54523-fig1-data1.zip › Figure 1/e/5.P16.jpg]

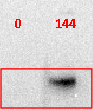

Supplement: Figure 1—source data 1. [file elife-54523-fig1-data1.zip › Figure 1/e/3.P27.jpg]

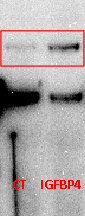

Supplement: Figure 1—source data 1. [file elife-54523-fig1-data1.zip › Figure 1/e/1.RB.jpg]

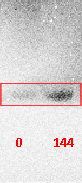

Supplement: Figure 1—source data 1. [file elife-54523-fig1-data1.zip › Figure 1/e/4.P21.jpg]

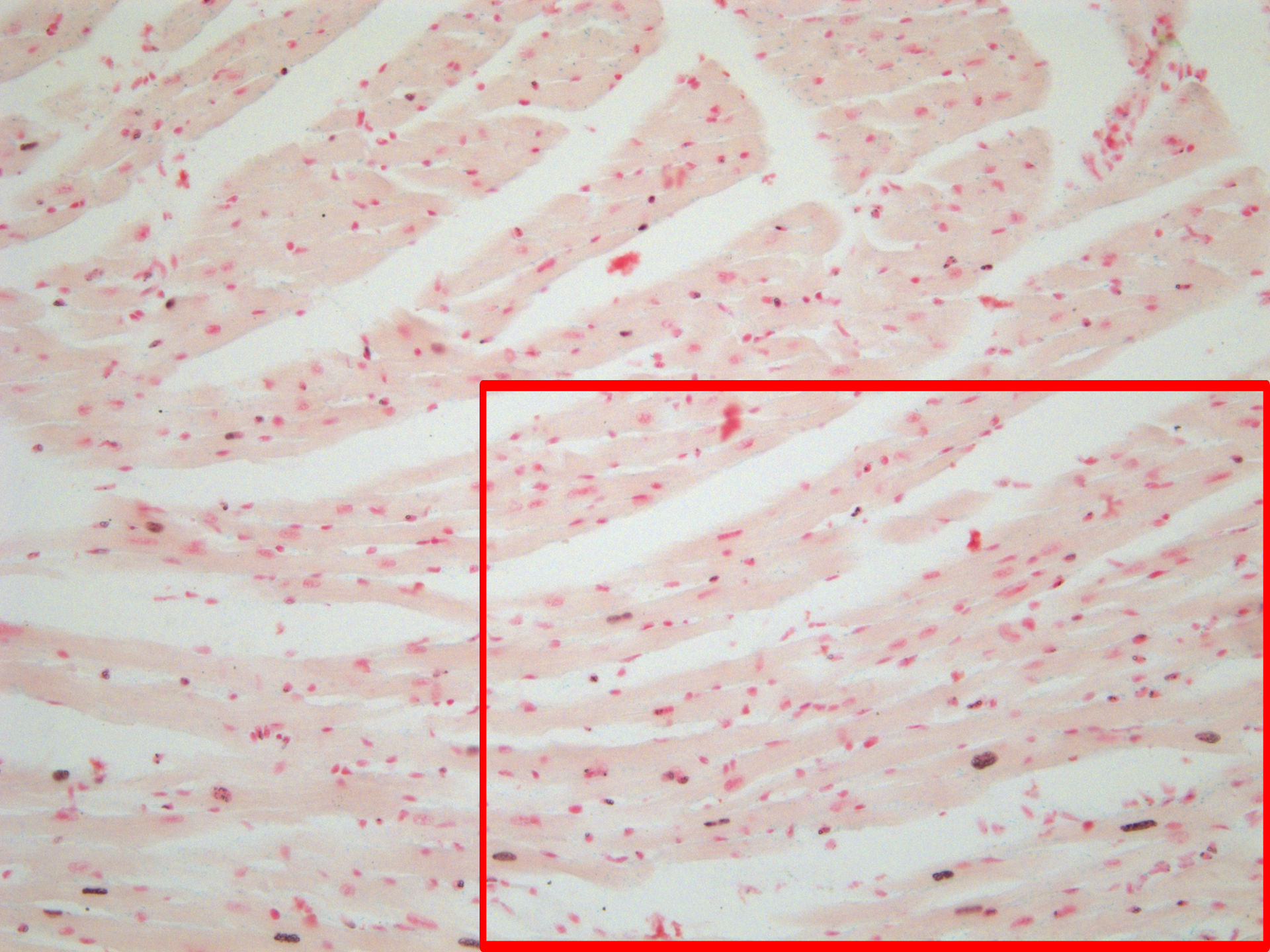

Supplement: Figure 1—source data 1. [file elife-54523-fig1-data1.zip › Figure 1/b/heart 0h.tif]

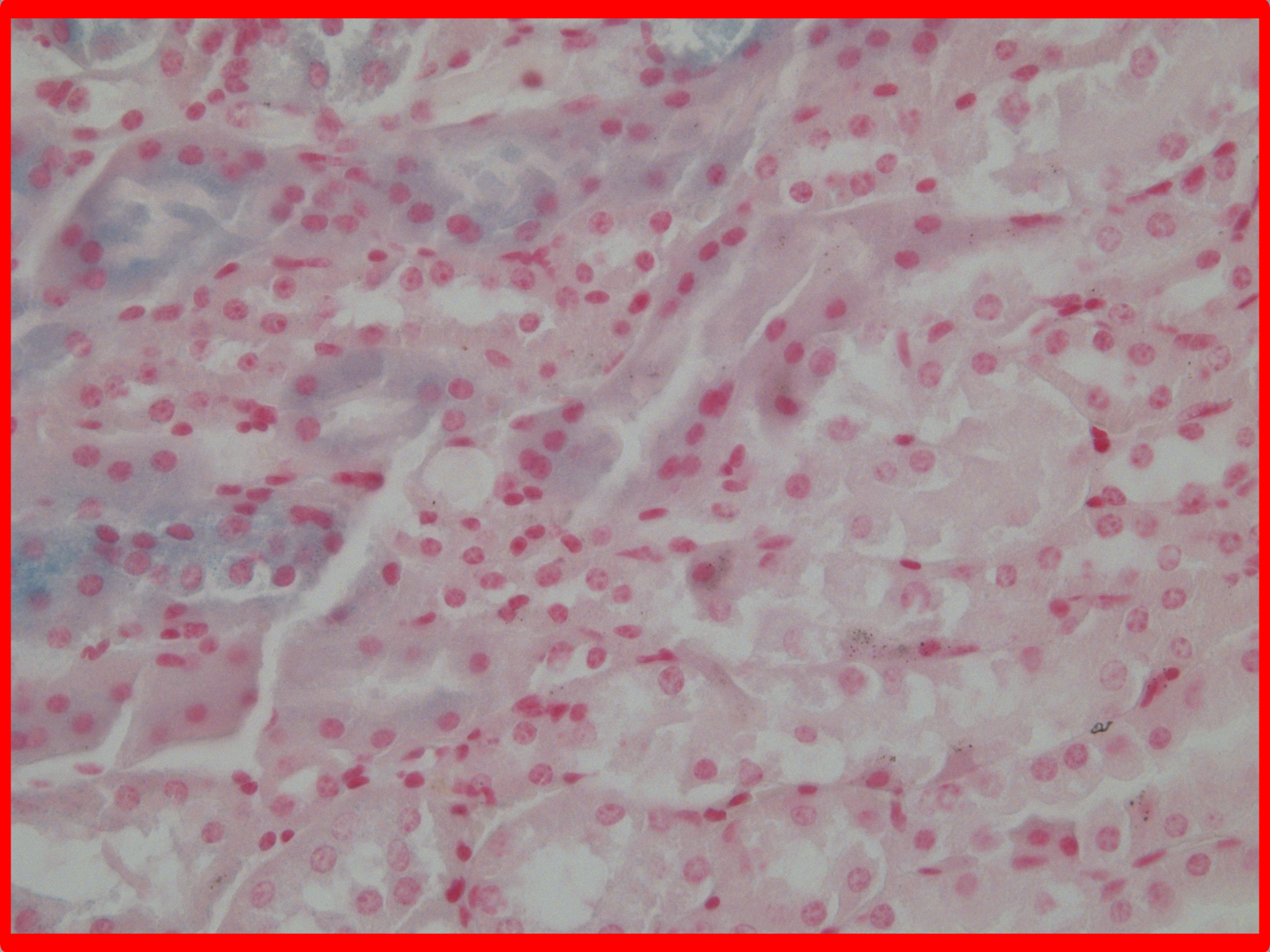

Supplement: Figure 1—source data 1. [file elife-54523-fig1-data1.zip › Figure 1/b/kidney 144h.tif]

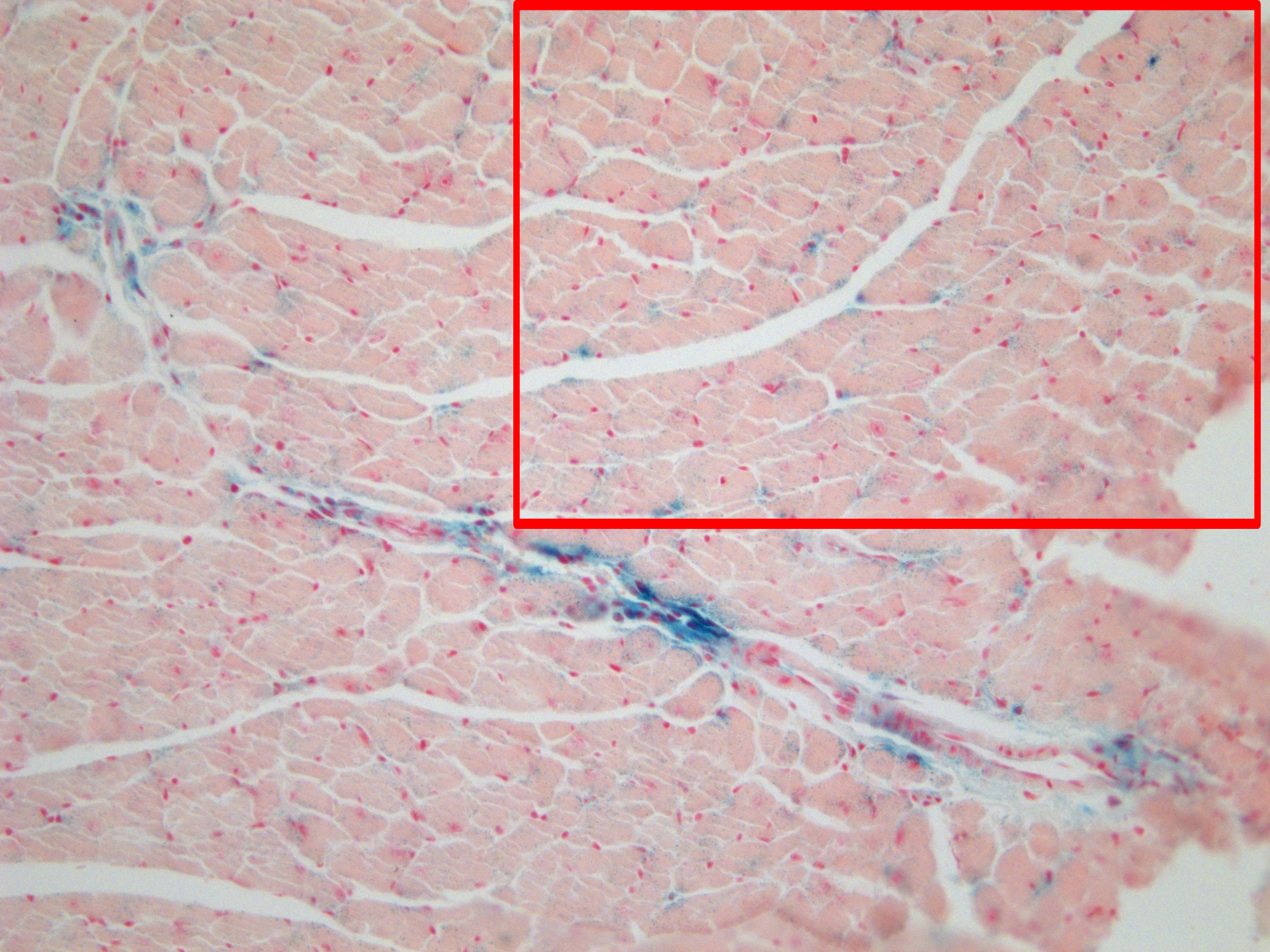

Supplement: Figure 1—source data 1. [file elife-54523-fig1-data1.zip › Figure 1/b/heart 144h.tif]

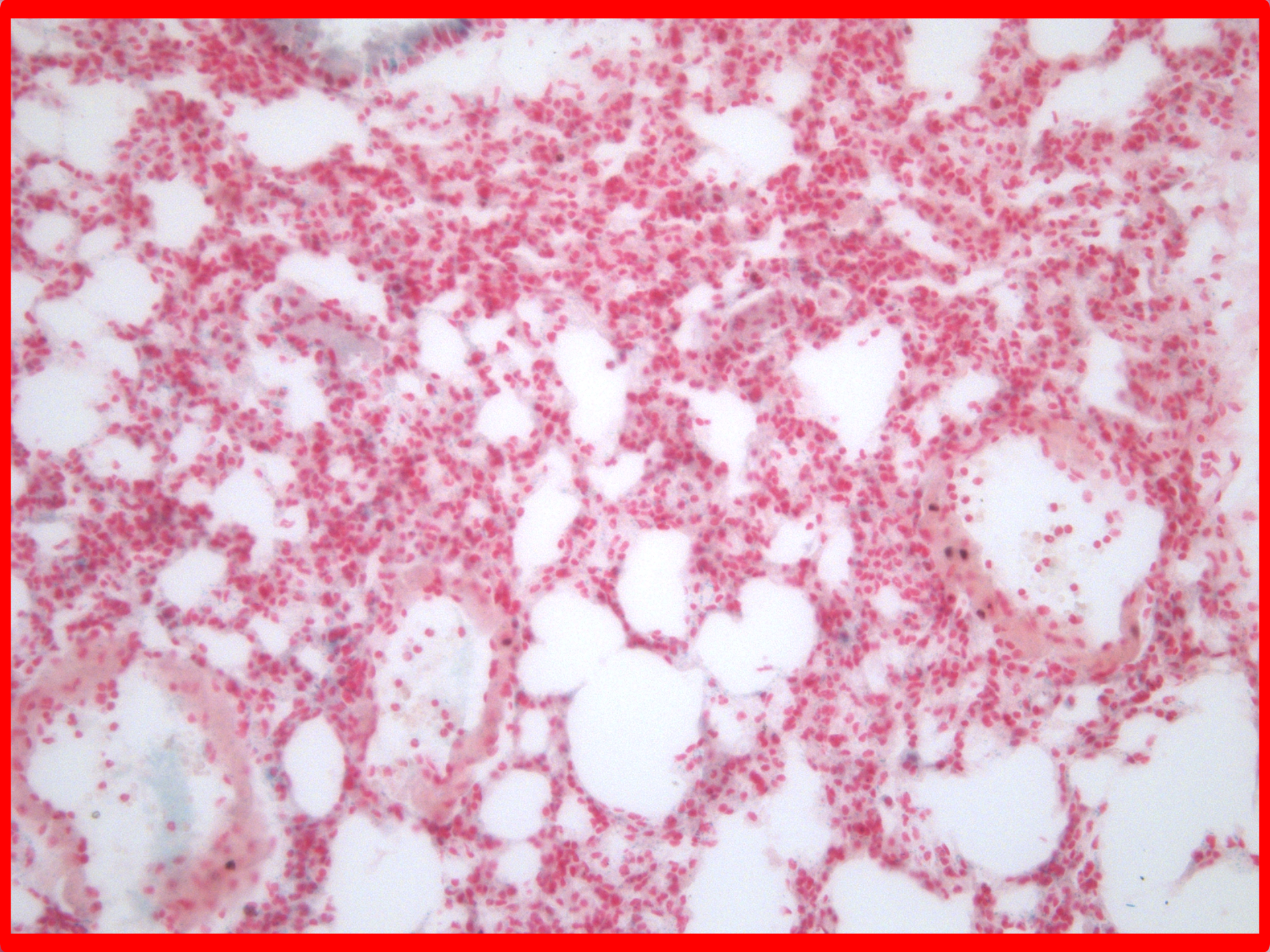

Supplement: Figure 1—source data 1. [file elife-54523-fig1-data1.zip › Figure 1/b/Lung 0h.tif]

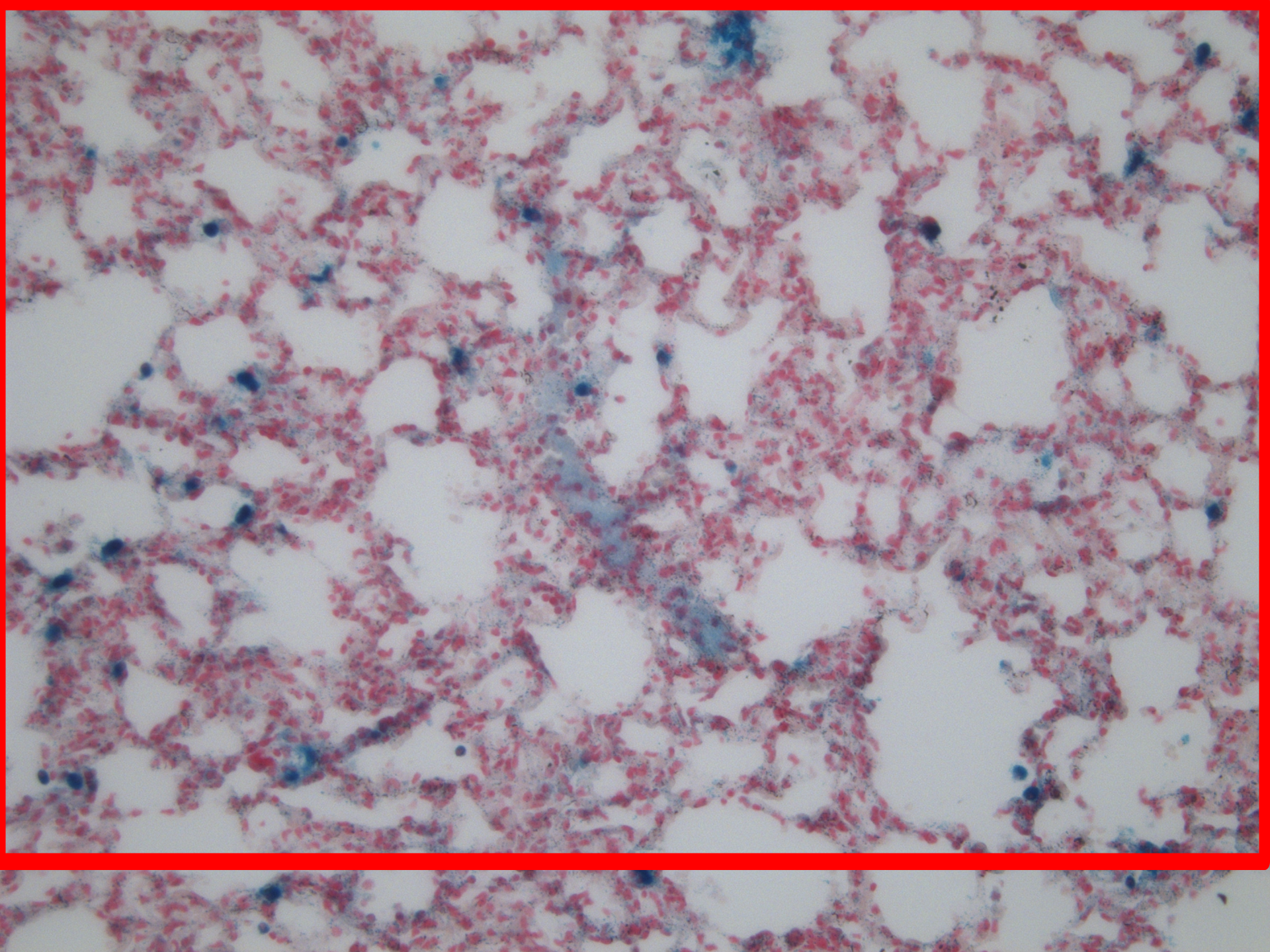

Supplement: Figure 1—source data 1. [file elife-54523-fig1-data1.zip › Figure 1/b/Lung 144h.tif]

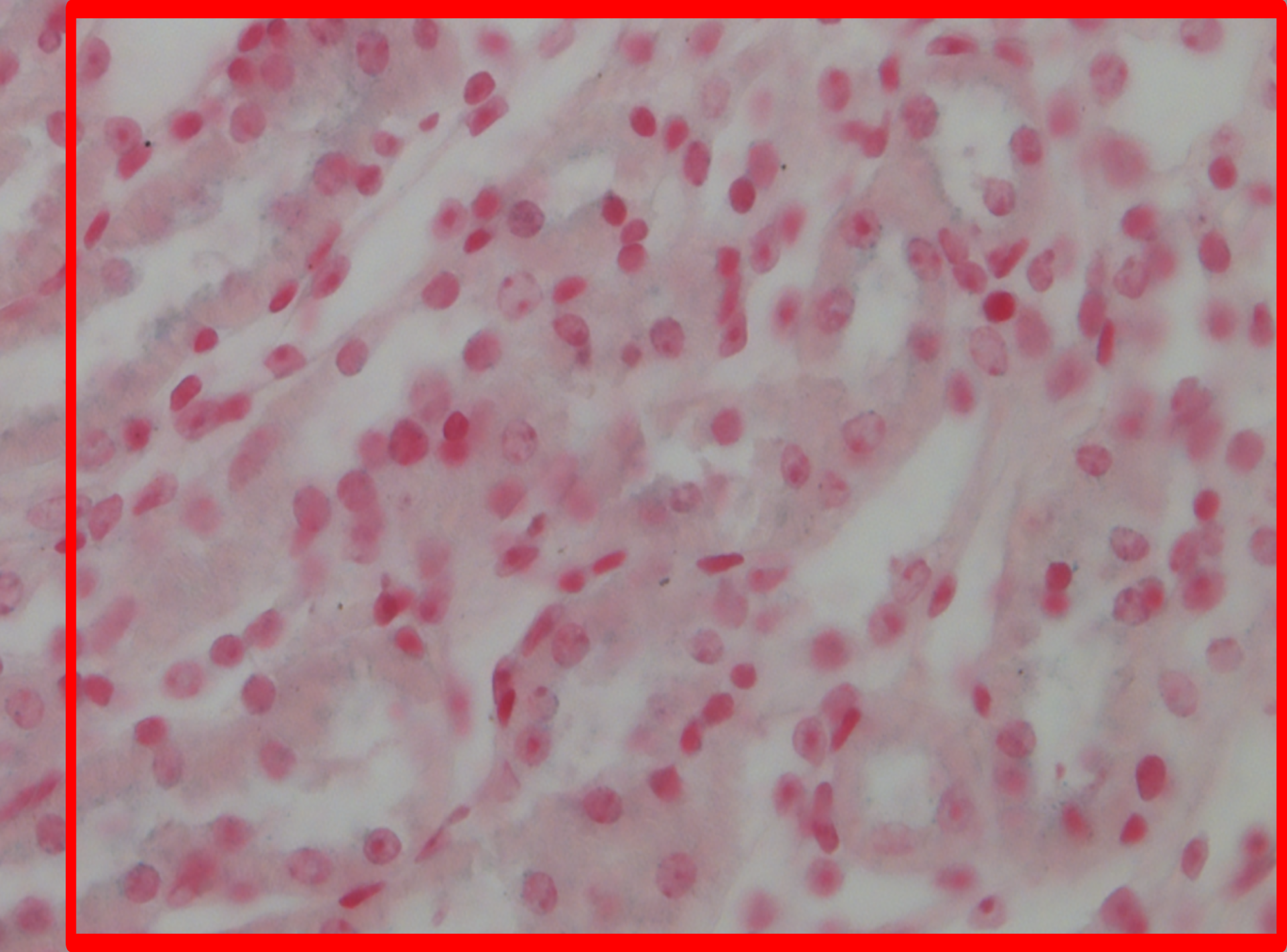

Supplement: Figure 1—source data 1. [file elife-54523-fig1-data1.zip › Figure 1/b/Kidney 0h.tif]

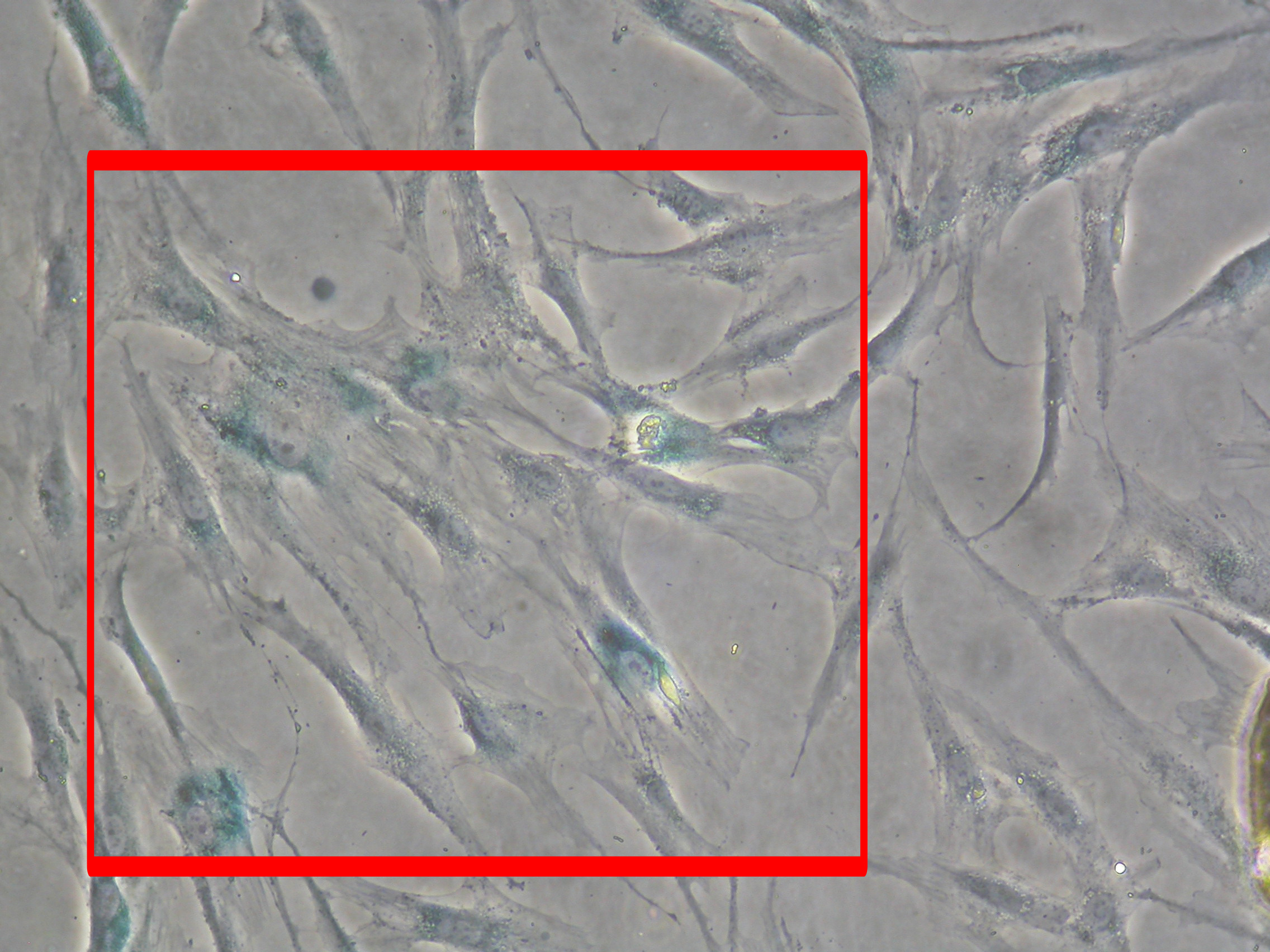

Supplement: Figure 1—figure supplement 1—source data 1. [file elife-54523-fig1-figsupp1-data1.zip › Figure 1ΓÇöfigure supplement 1/a/DOXO SUP File 1.JPG]

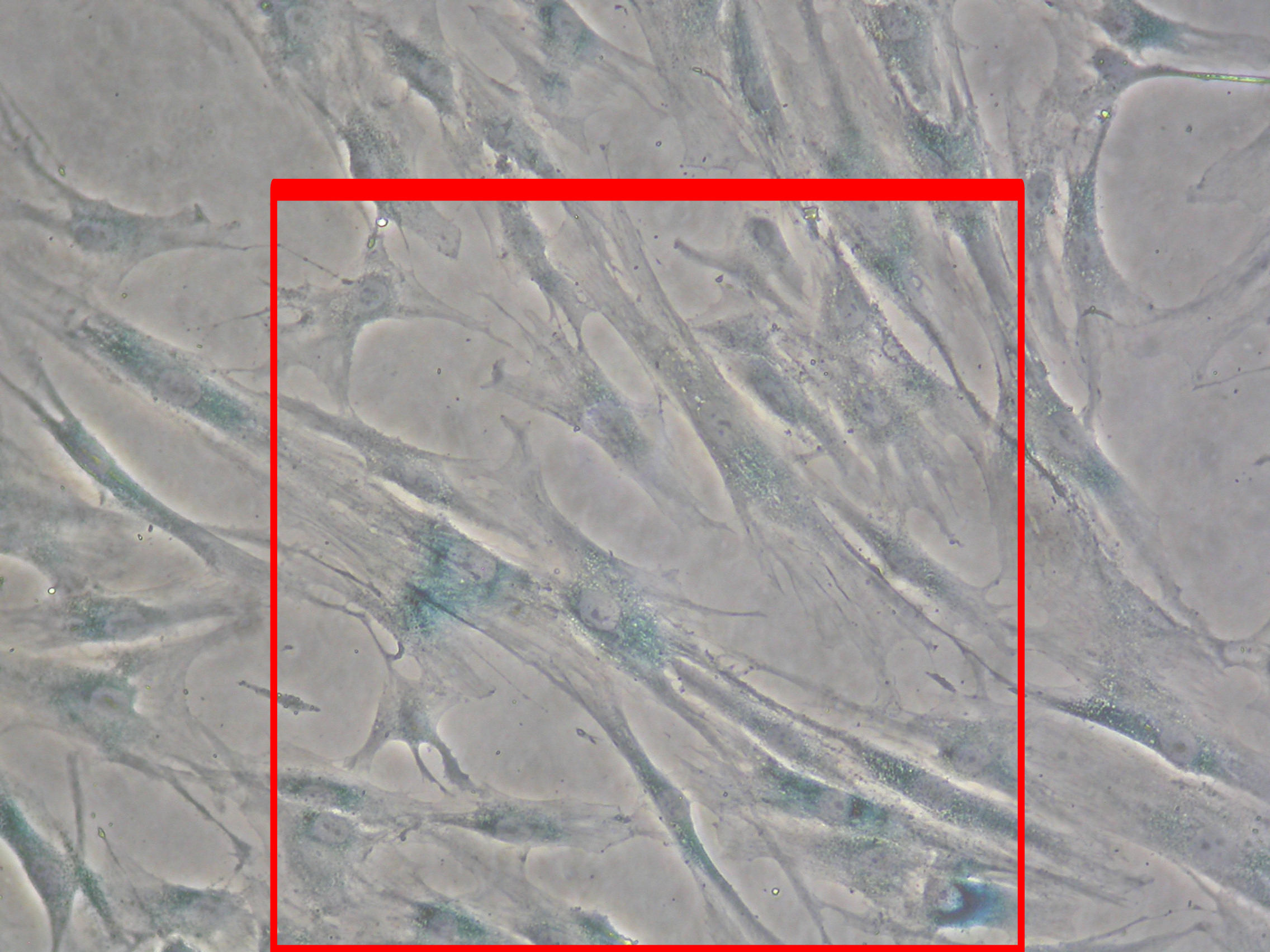

Supplement: Figure 1—figure supplement 1—source data 1. [file elife-54523-fig1-figsupp1-data1.zip › Figure 1ΓÇöfigure supplement 1/a/IRH SUP File 1.JPG]

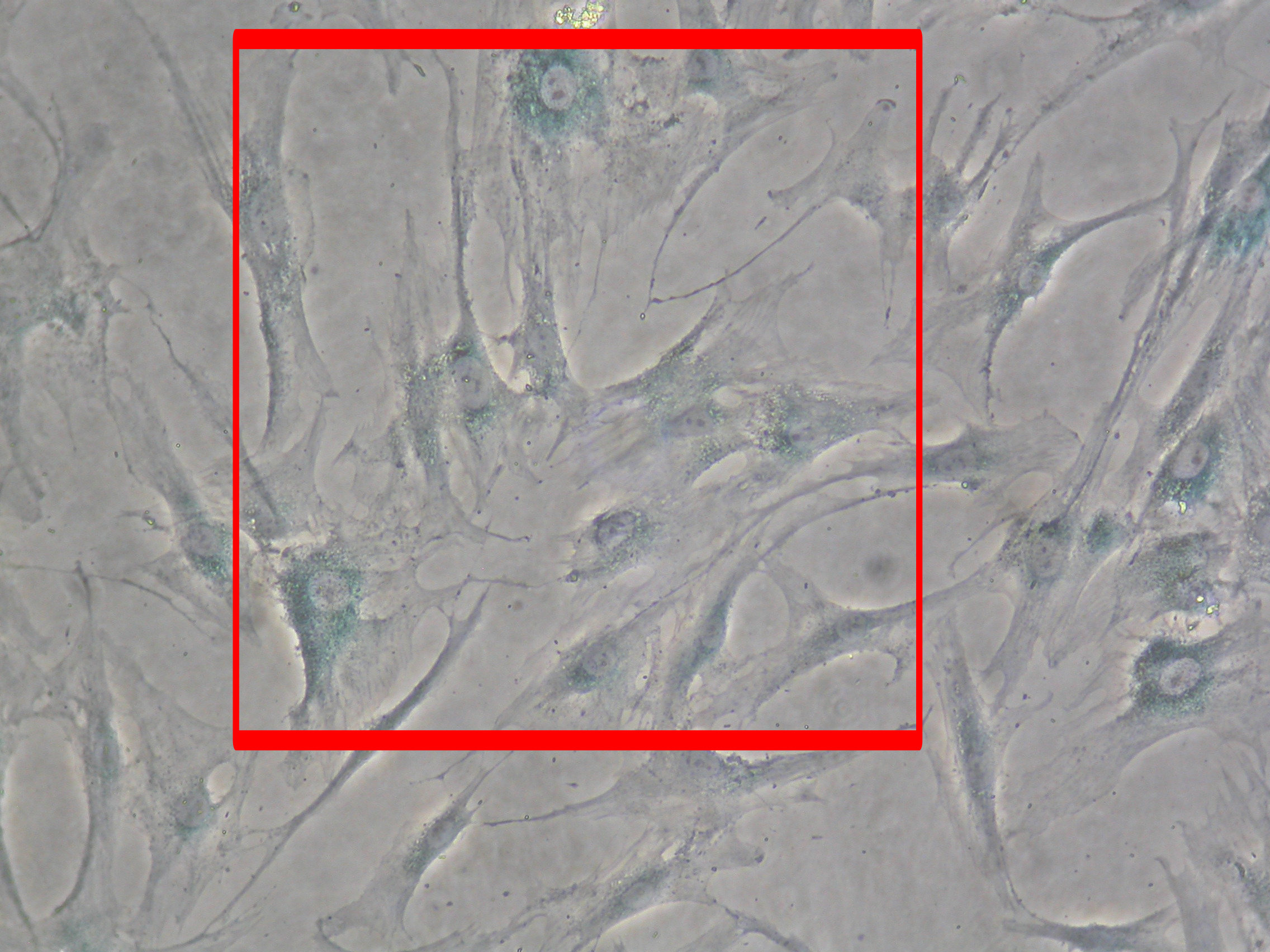

Supplement: Figure 1—figure supplement 1—source data 1. [file elife-54523-fig1-figsupp1-data1.zip › Figure 1ΓÇöfigure supplement 1/a/REP SUP File 1.JPG]

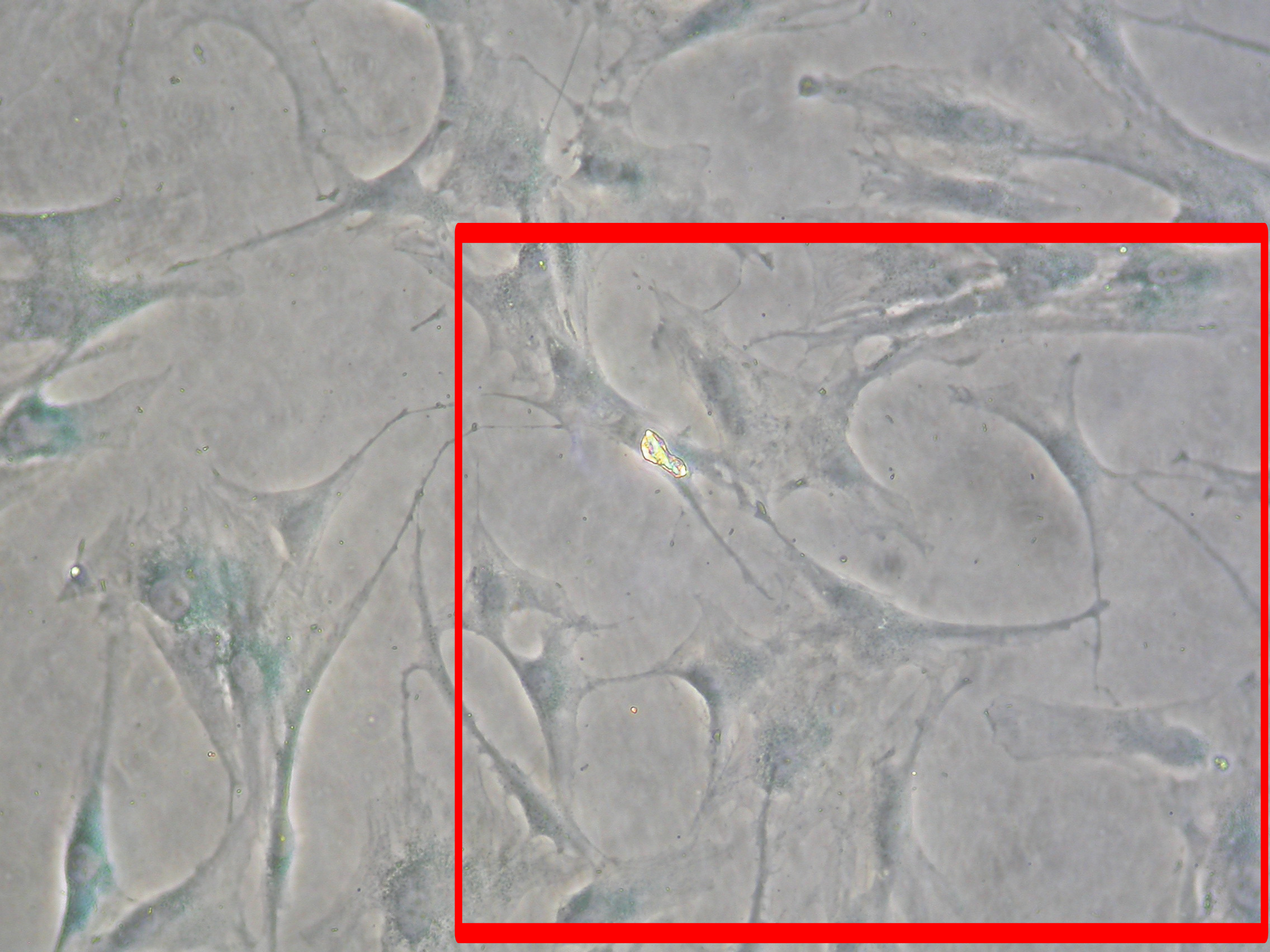

Supplement: Figure 1—figure supplement 1—source data 1. [file elife-54523-fig1-figsupp1-data1.zip › Figure 1ΓÇöfigure supplement 1/a/CTRL SUP File1.JPG]

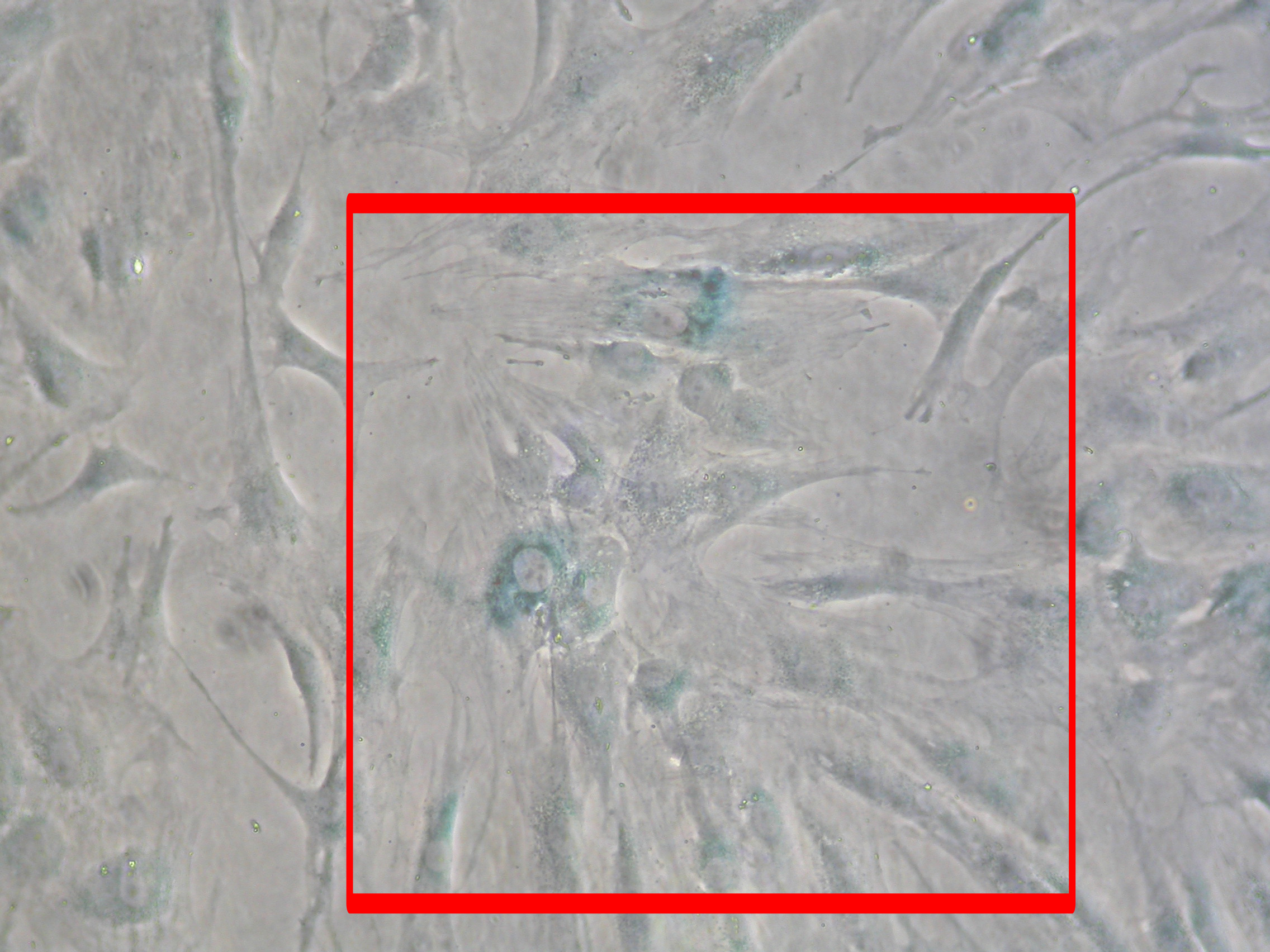

Supplement: Figure 1—figure supplement 1—source data 1. [file elife-54523-fig1-figsupp1-data1.zip › Figure 1ΓÇöfigure supplement 1/a/H2O2 SUP File 1.JPG]

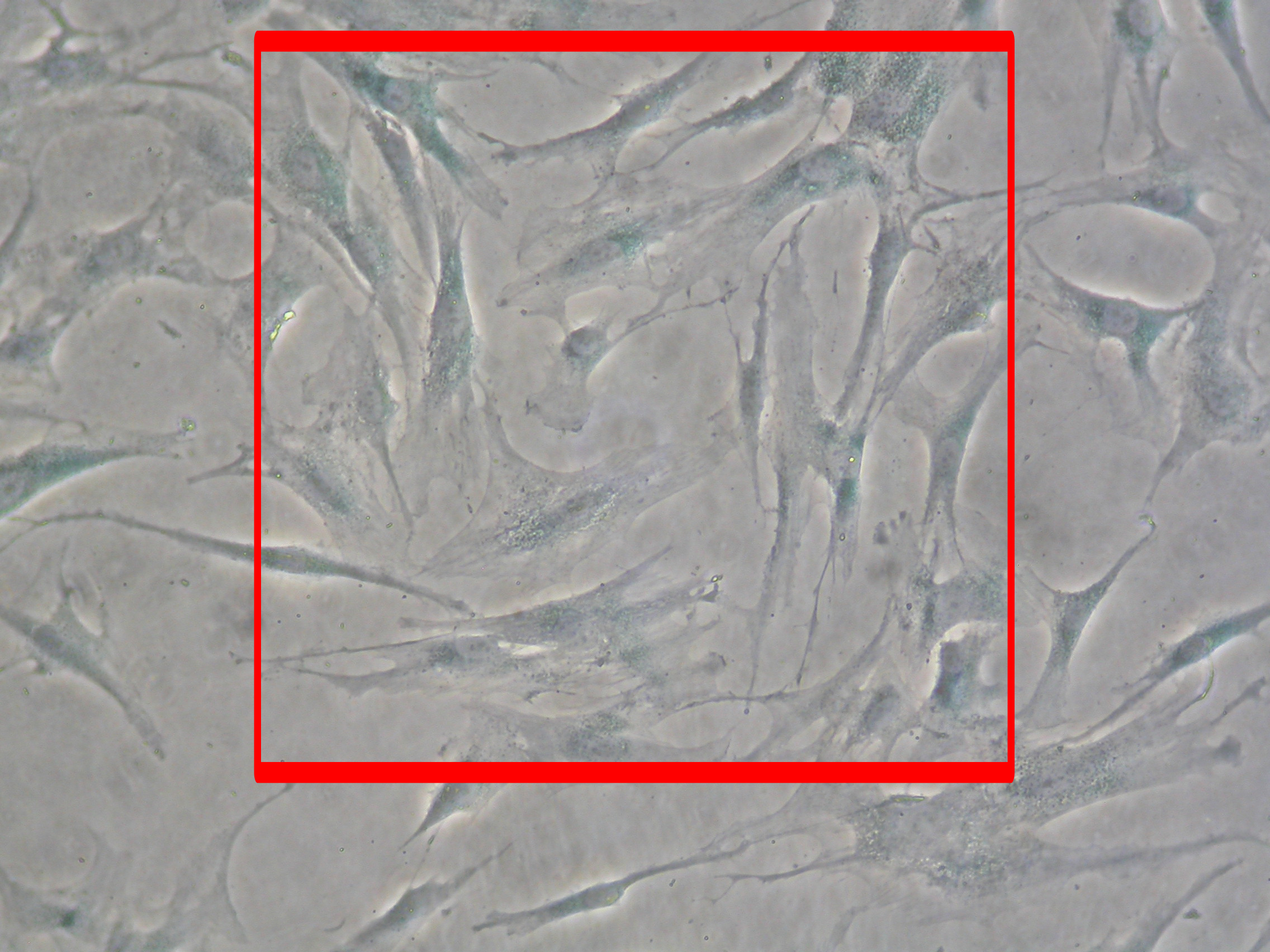

Supplement: Figure 1—figure supplement 1—source data 1. [file elife-54523-fig1-figsupp1-data1.zip › Figure 1ΓÇöfigure supplement 1/a/IRL SUP File1.JPG]

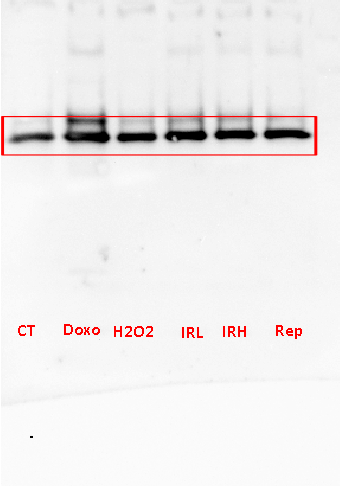

Supplement: Figure 1—figure supplement 1—source data 1. [file elife-54523-fig1-figsupp1-data1.zip › Figure 1ΓÇöfigure supplement 1/c/WB .tif]

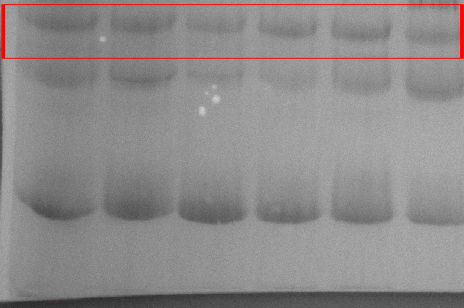

Supplement: Figure 1—figure supplement 1—source data 1. [file elife-54523-fig1-figsupp1-data1.zip › Figure 1ΓÇöfigure supplement 1/c/LC2.jpg]

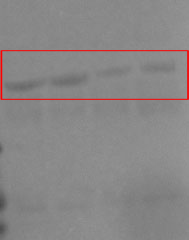

Supplement: Figure 1—figure supplement 1—source data 1. [file elife-54523-fig1-figsupp1-data1.zip › Figure 1ΓÇöfigure supplement 1/d/LC.jpg]

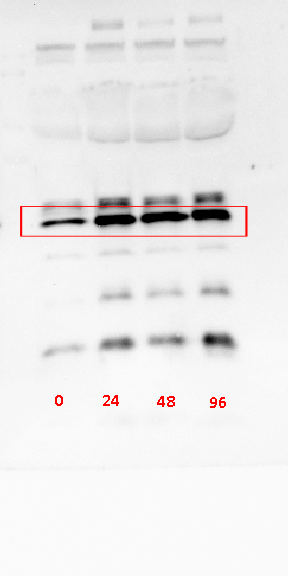

Supplement: Figure 1—figure supplement 1—source data 1. [file elife-54523-fig1-figsupp1-data1.zip › Figure 1ΓÇöfigure supplement 1/d/WB .tif]

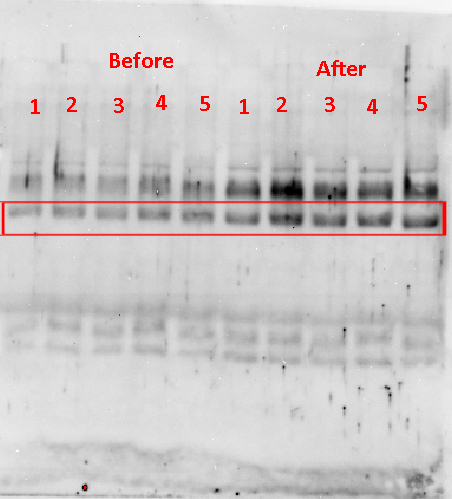

Supplement: Figure 2—source data 1. [file elife-54523-fig2-data1.zip › Figure 2/Sera 1-5 WB.jpg]

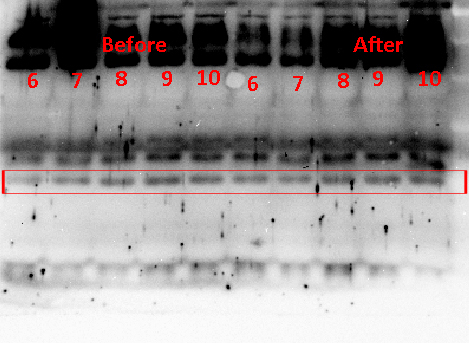

Supplement: Figure 2—source data 1. [file elife-54523-fig2-data1.zip › Figure 2/Sera 6-10 WB.jpg]

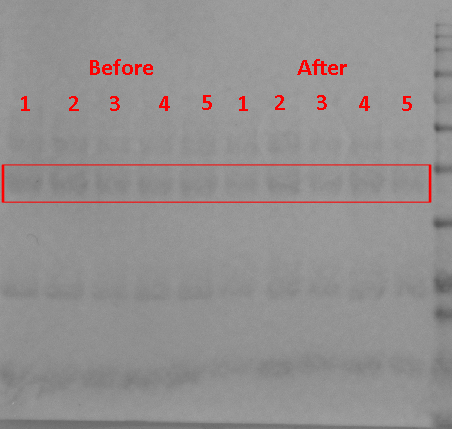

Supplement: Figure 2—source data 1. [file elife-54523-fig2-data1.zip › Figure 2/Sera 1-5 LC.jpg]

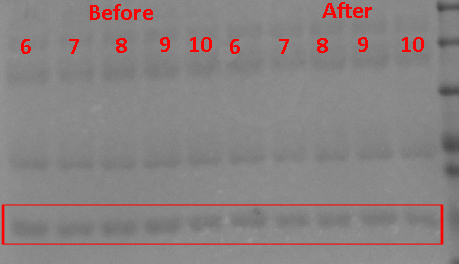

Supplement: Figure 2—source data 1. [file elife-54523-fig2-data1.zip › Figure 2/Sera 6-10 LC.jpg]

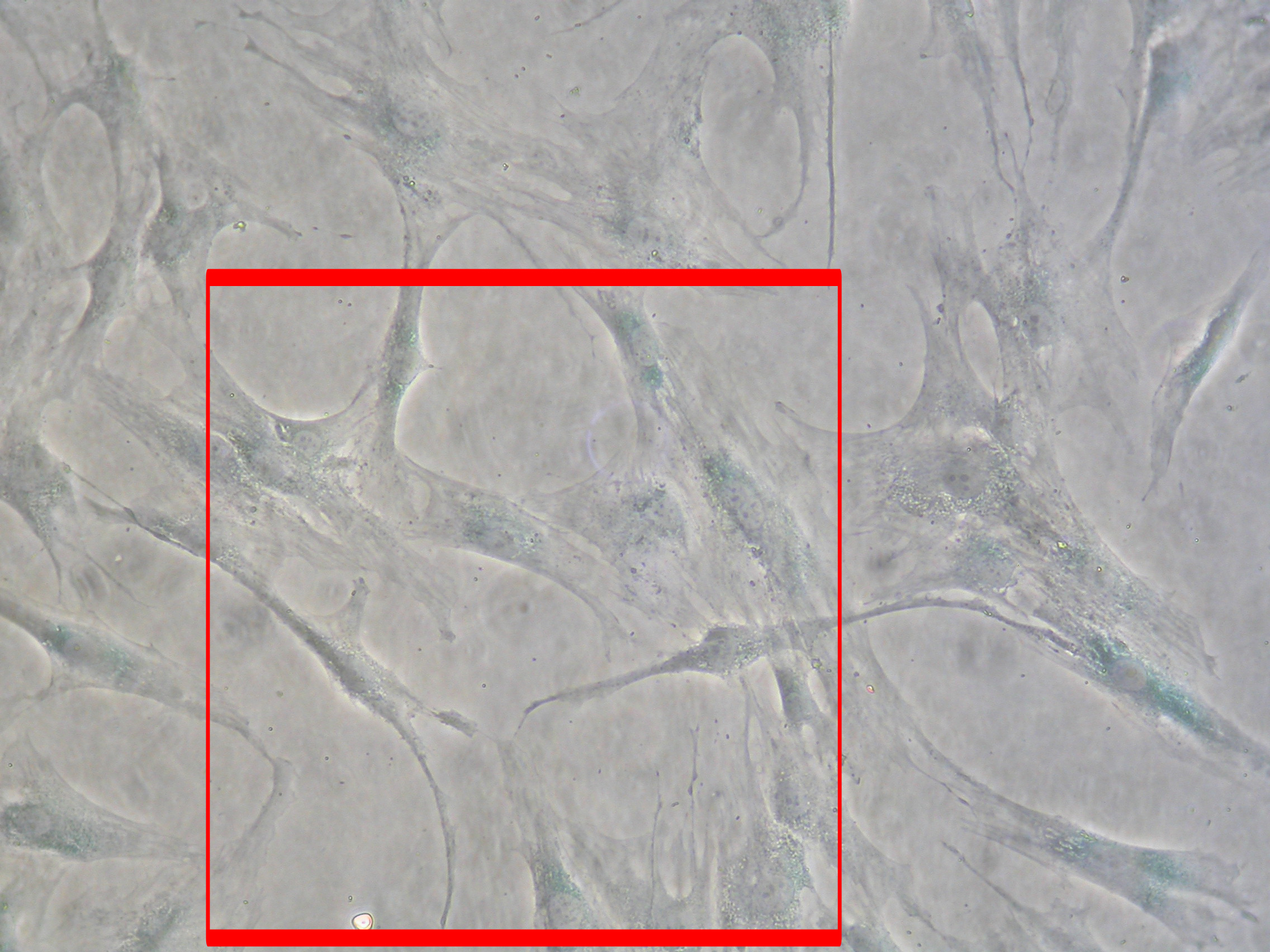

Supplement: Figure 3—source data 1. [file elife-54523-fig3-data1.zip › Figure 3/a/CTRL + PBX.JPG]

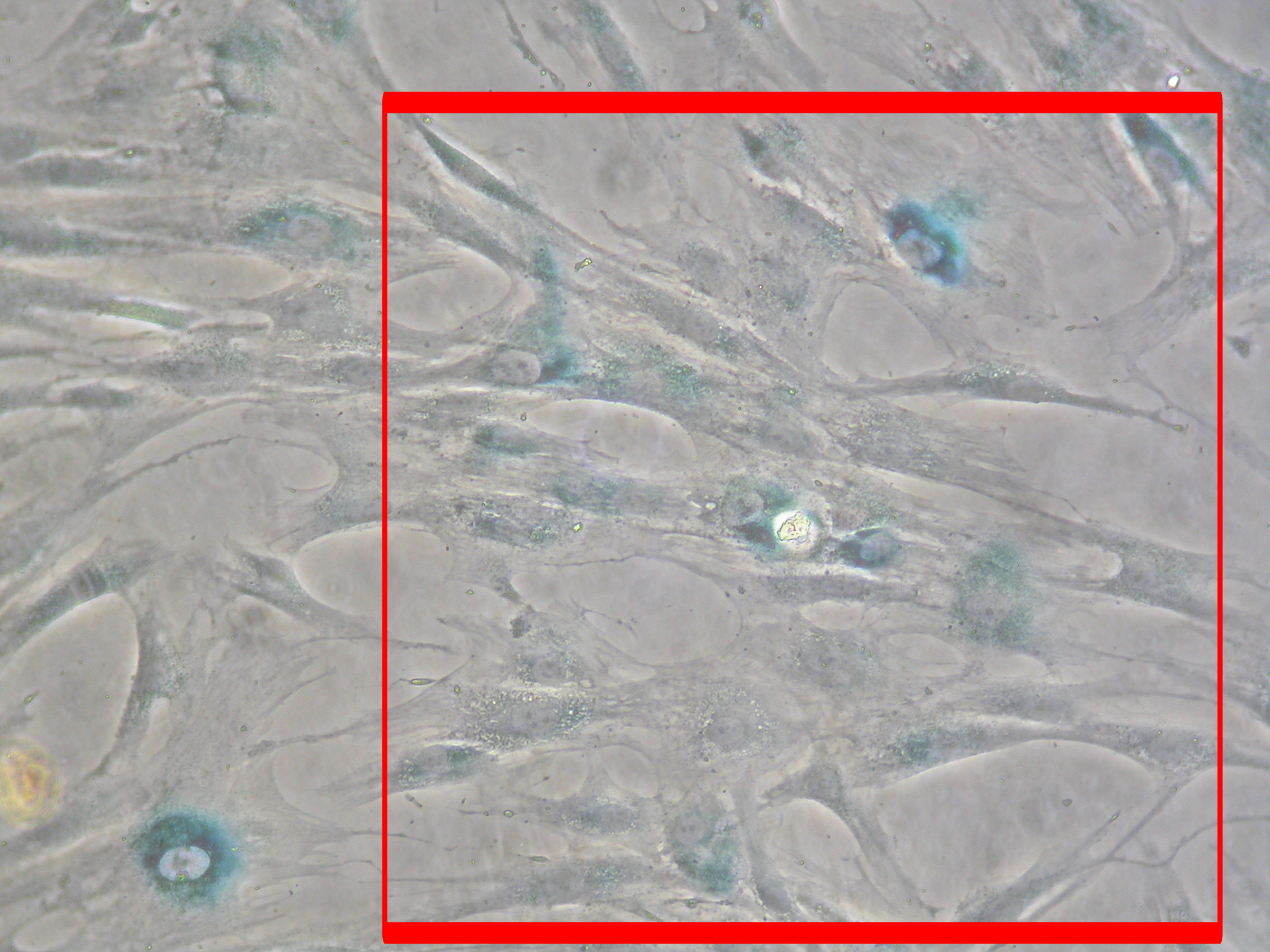

Supplement: Figure 3—source data 1. [file elife-54523-fig3-data1.zip › Figure 3/a/H2O2 - PBX.JPG]

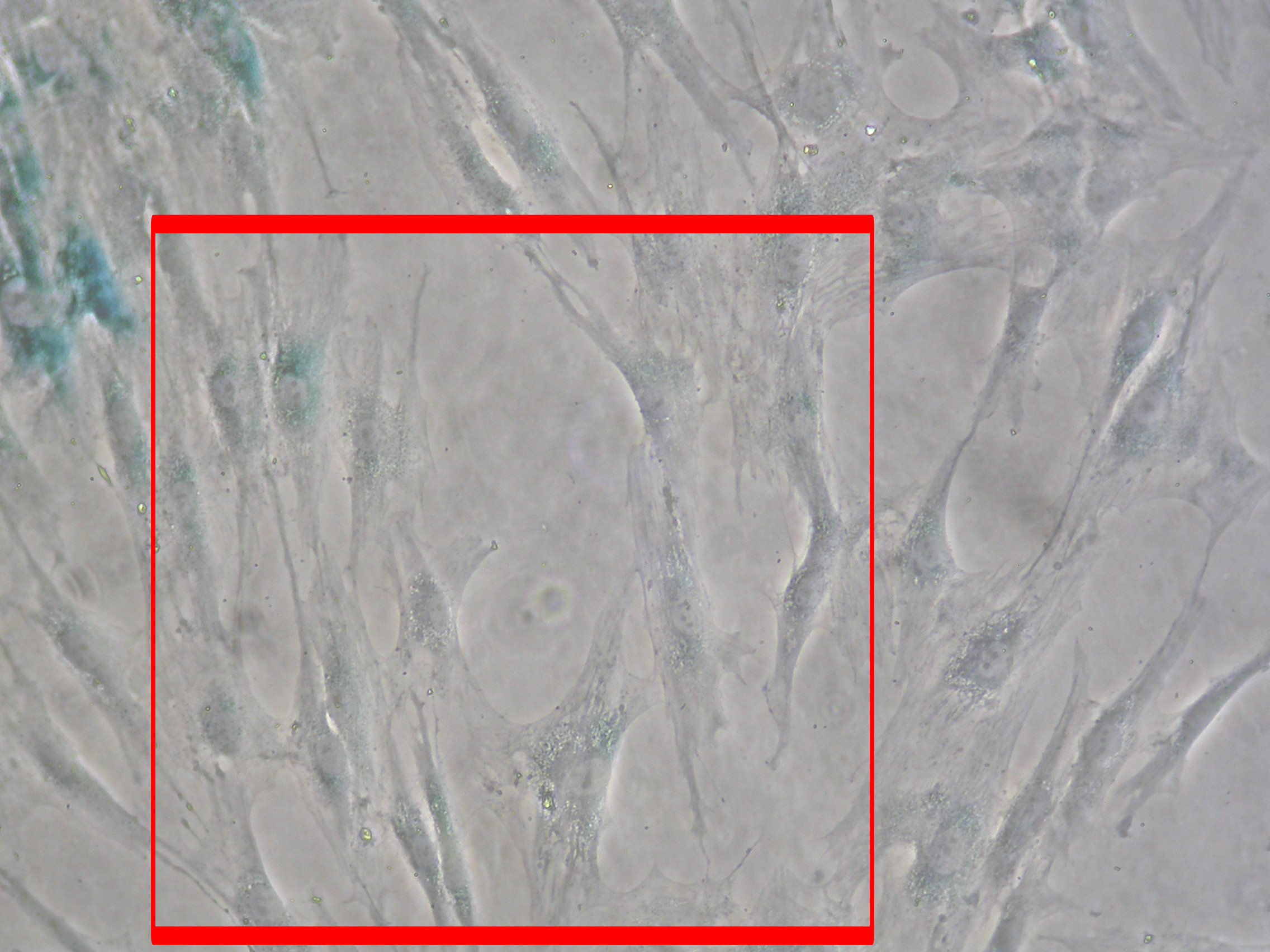

Supplement: Figure 3—source data 1. [file elife-54523-fig3-data1.zip › Figure 3/a/PGE2 -PBX.JPG]

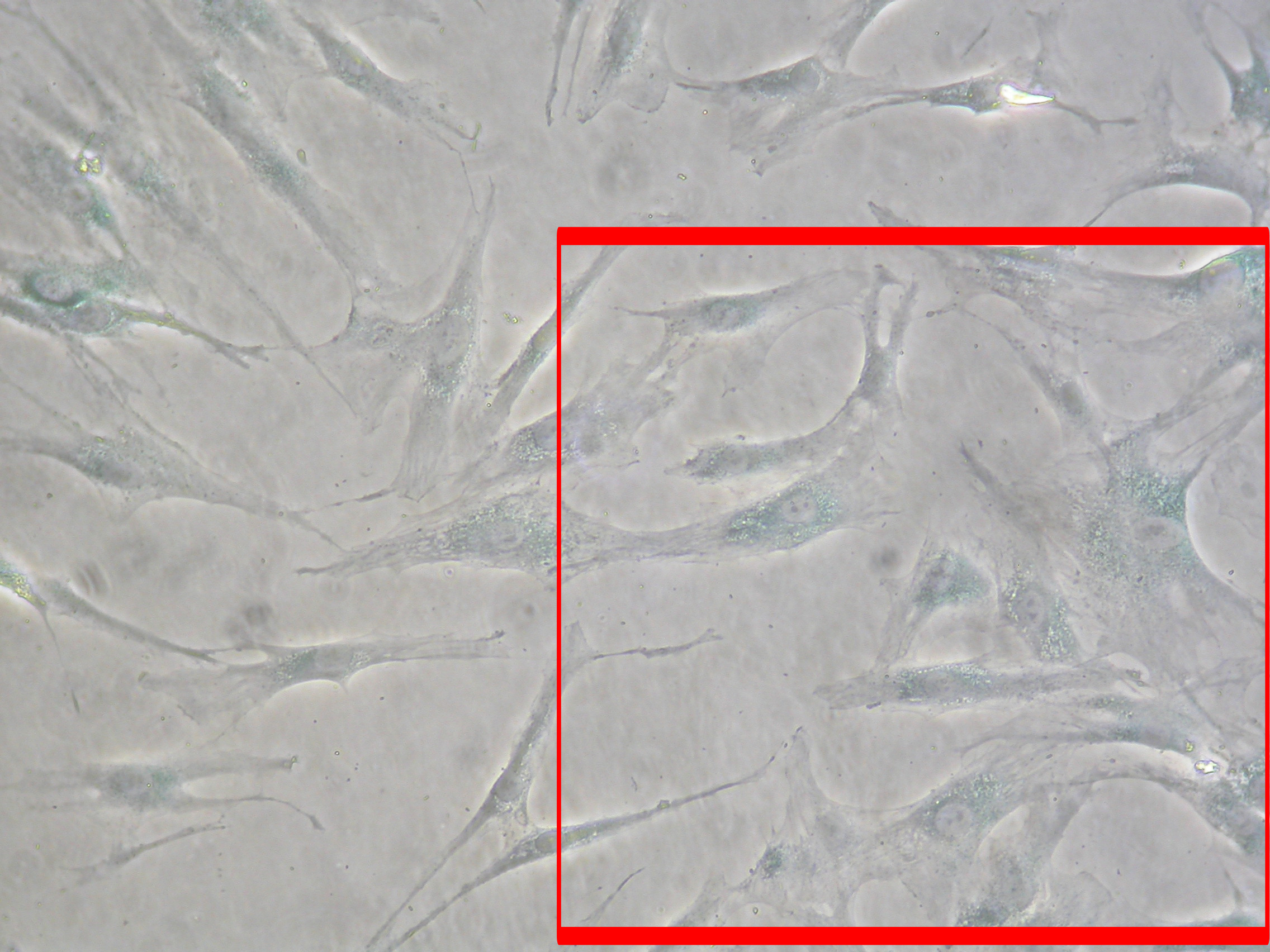

Supplement: Figure 3—source data 1. [file elife-54523-fig3-data1.zip › Figure 3/a/PGE2 + PXB.JPG]

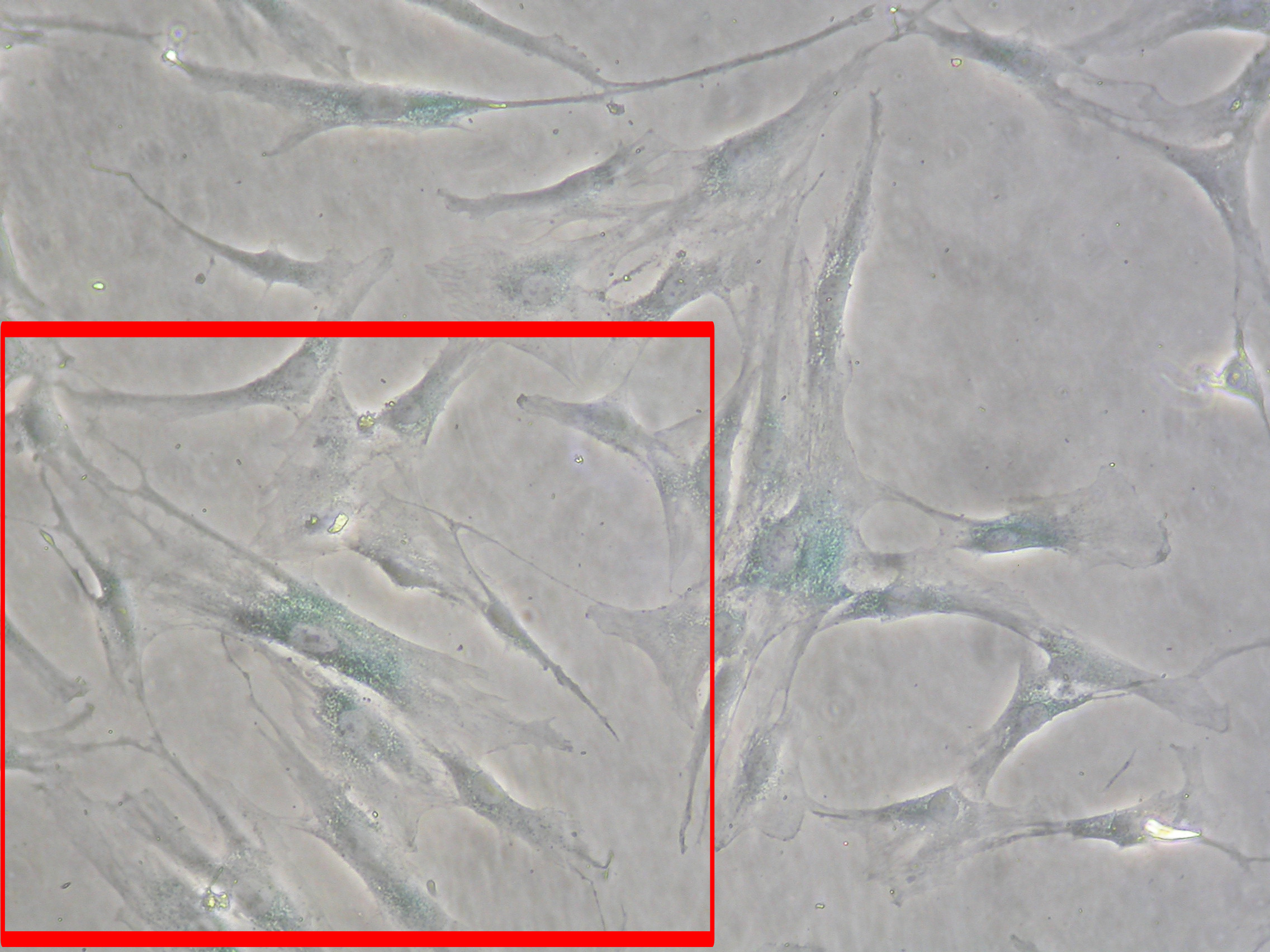

Supplement: Figure 3—source data 1. [file elife-54523-fig3-data1.zip › Figure 3/a/H2O2 + PXB.JPG]

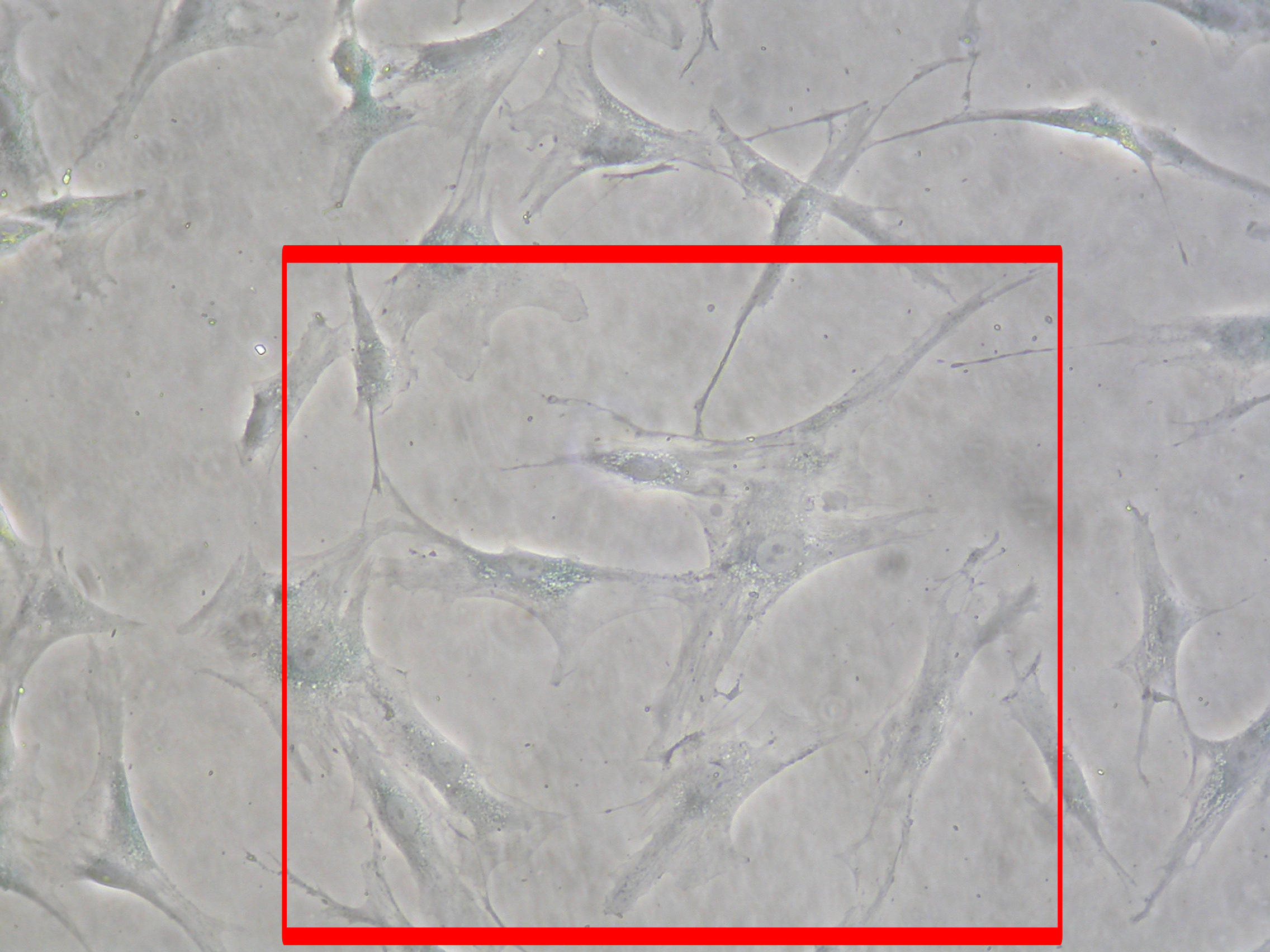

Supplement: Figure 3—source data 1. [file elife-54523-fig3-data1.zip › Figure 3/a/CTRL - PBX.JPG]

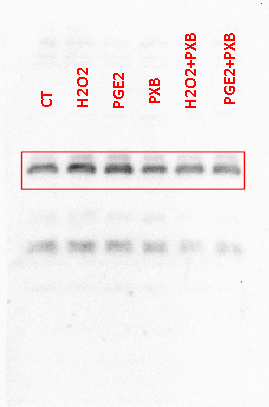

Supplement: Figure 3—source data 1. [file elife-54523-fig3-data1.zip › Figure 3/c/3C.jpg]

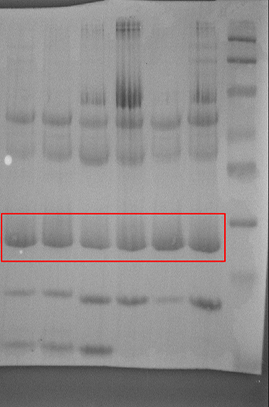

Supplement: Figure 3—source data 1. [file elife-54523-fig3-data1.zip › Figure 3/c/3C LC.jpg]

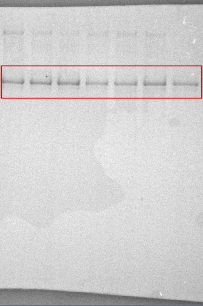

Supplement: Figure 4—source data 1. [file elife-54523-fig4-data1.zip › Figure 4/c/4C LC.jpg]

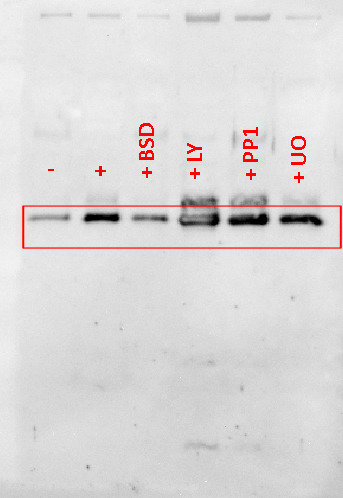

Supplement: Figure 4—source data 1. [file elife-54523-fig4-data1.zip › Figure 4/c/4C WB.jpg]

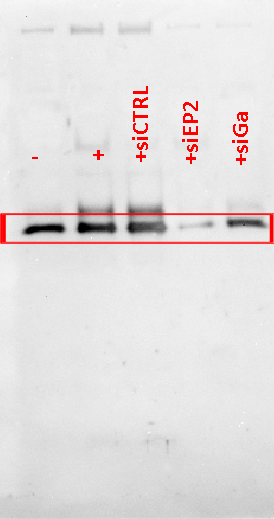

Supplement: Figure 4—source data 1. [file elife-54523-fig4-data1.zip › Figure 4/b/4B WB.jpg]

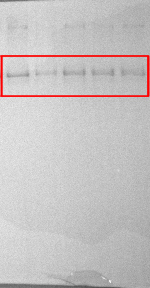

Supplement: Figure 4—source data 1. [file elife-54523-fig4-data1.zip › Figure 4/b/4B LC.jpg]

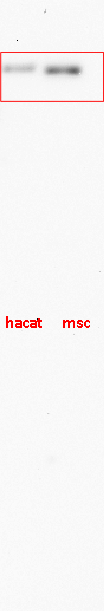

Supplement: Figure 4—figure supplement 1—source data 1. [file elife-54523-fig4-figsupp1-data1.zip › Figure 4ΓÇöfigure supplement 1/j.tif]

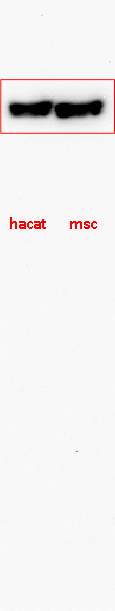

Supplement: Figure 4—figure supplement 1—source data 1. [file elife-54523-fig4-figsupp1-data1.zip › Figure 4ΓÇöfigure supplement 1/i.tif]

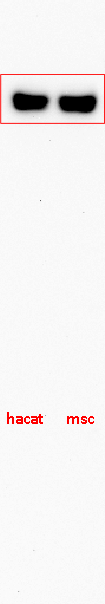

Supplement: Figure 4—figure supplement 1—source data 1. [file elife-54523-fig4-figsupp1-data1.zip › Figure 4ΓÇöfigure supplement 1/h.tif]

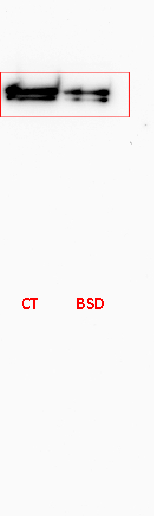

Supplement: Figure 4—figure supplement 1—source data 1. [file elife-54523-fig4-figsupp1-data1.zip › Figure 4ΓÇöfigure supplement 1/g/pMEK.tif]

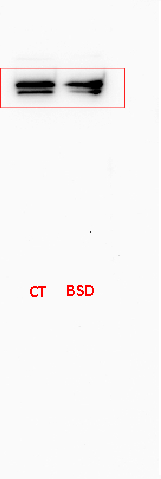

Supplement: Figure 4—figure supplement 1—source data 1. [file elife-54523-fig4-figsupp1-data1.zip › Figure 4ΓÇöfigure supplement 1/g/MEK tot.tif]

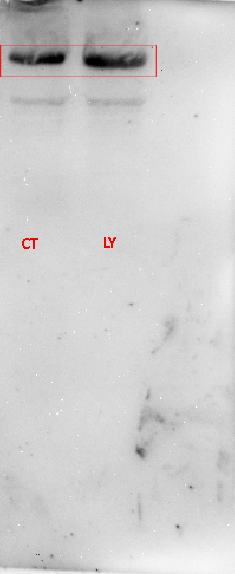

Supplement: Figure 4—figure supplement 1—source data 1. [file elife-54523-fig4-figsupp1-data1.zip › Figure 4ΓÇöfigure supplement 1/f/AKT tot.tif]

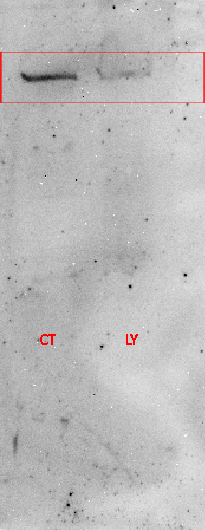

Supplement: Figure 4—figure supplement 1—source data 1. [file elife-54523-fig4-figsupp1-data1.zip › Figure 4ΓÇöfigure supplement 1/f/pAKT.tif]

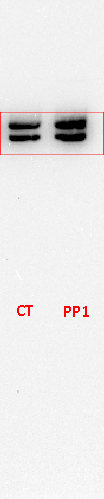

Supplement: Figure 4—figure supplement 1—source data 1. [file elife-54523-fig4-figsupp1-data1.zip › Figure 4ΓÇöfigure supplement 1/d/PI3K.jpg]

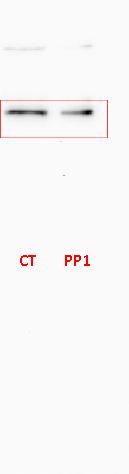

Supplement: Figure 4—figure supplement 1—source data 1. [file elife-54523-fig4-figsupp1-data1.zip › Figure 4ΓÇöfigure supplement 1/d/pPI3Ktyr.jpg]

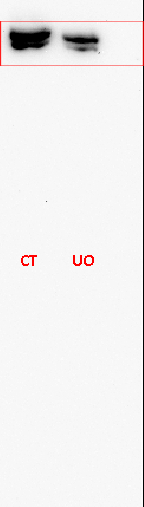

Supplement: Figure 4—figure supplement 1—source data 1. [file elife-54523-fig4-figsupp1-data1.zip › Figure 4ΓÇöfigure supplement 1/e/pERK.tif]

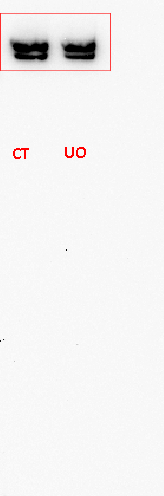

Supplement: Figure 4—figure supplement 1—source data 1. [file elife-54523-fig4-figsupp1-data1.zip › Figure 4ΓÇöfigure supplement 1/e/ERK tot.tif]

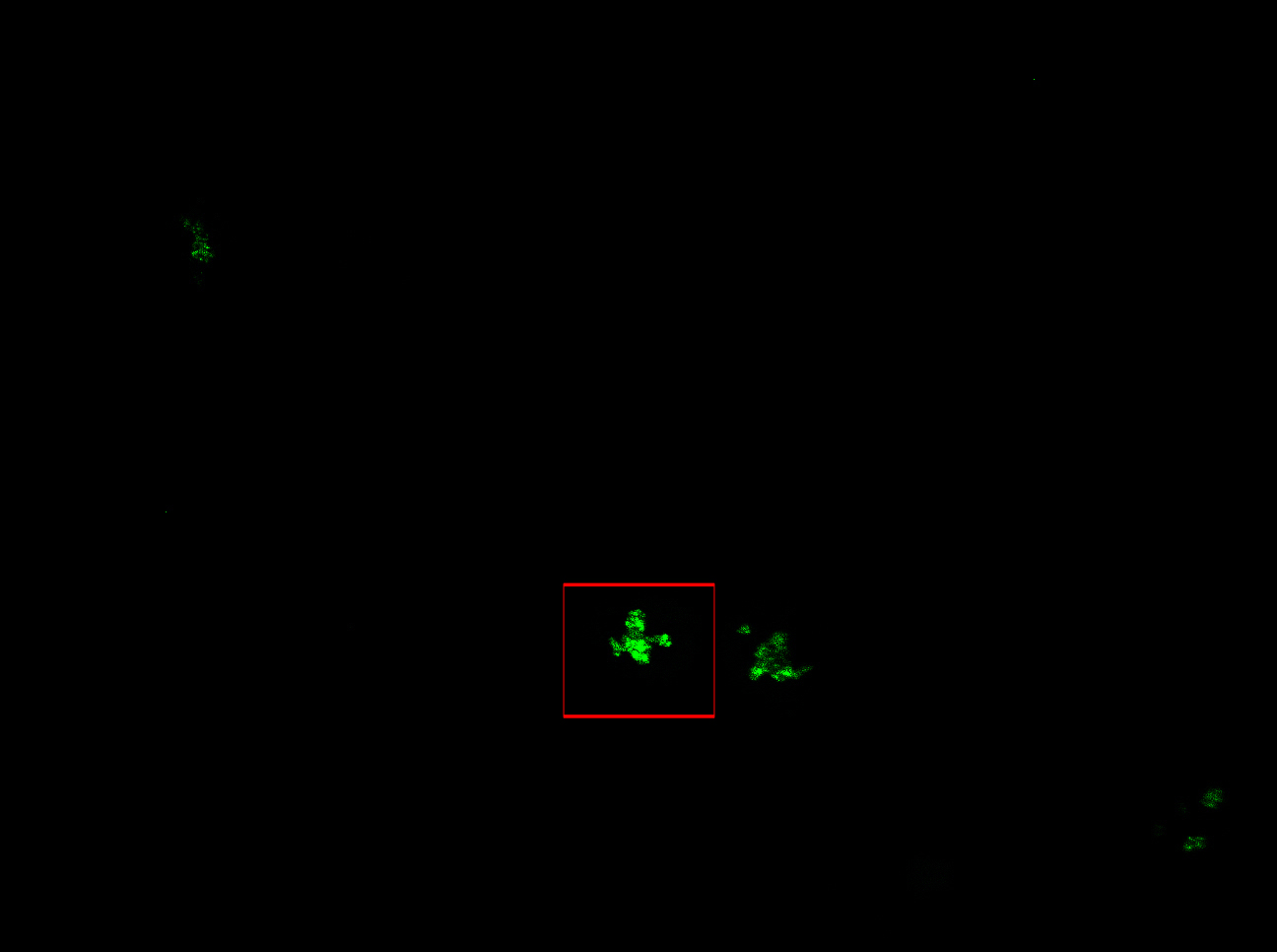

Supplement: Figure 5—source data 1. [file elife-54523-fig5-data1.zip › Figure 5/i/2_HP1a.jpg]

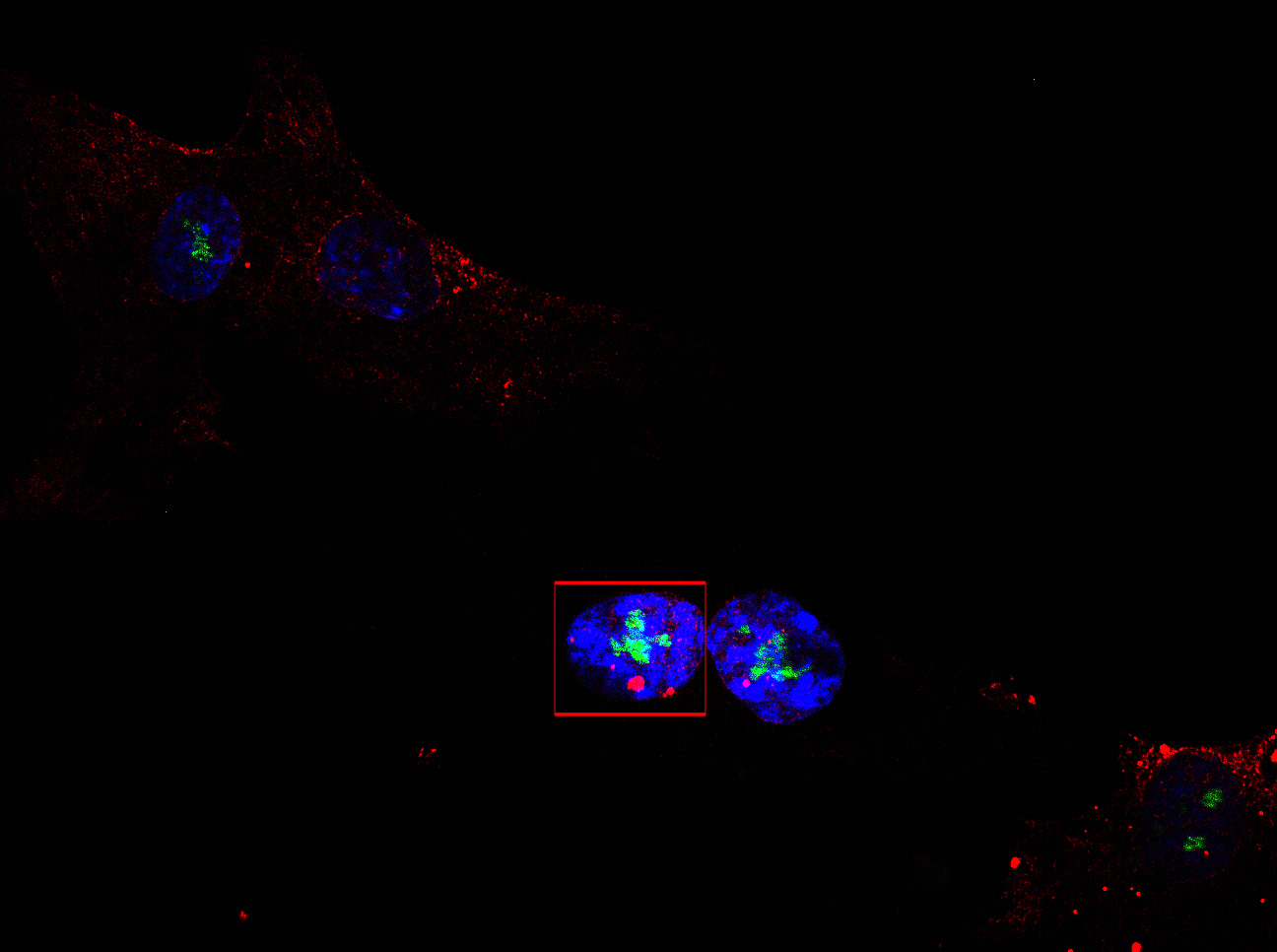

Supplement: Figure 5—source data 1. [file elife-54523-fig5-data1.zip › Figure 5/i/Merge.jpg]

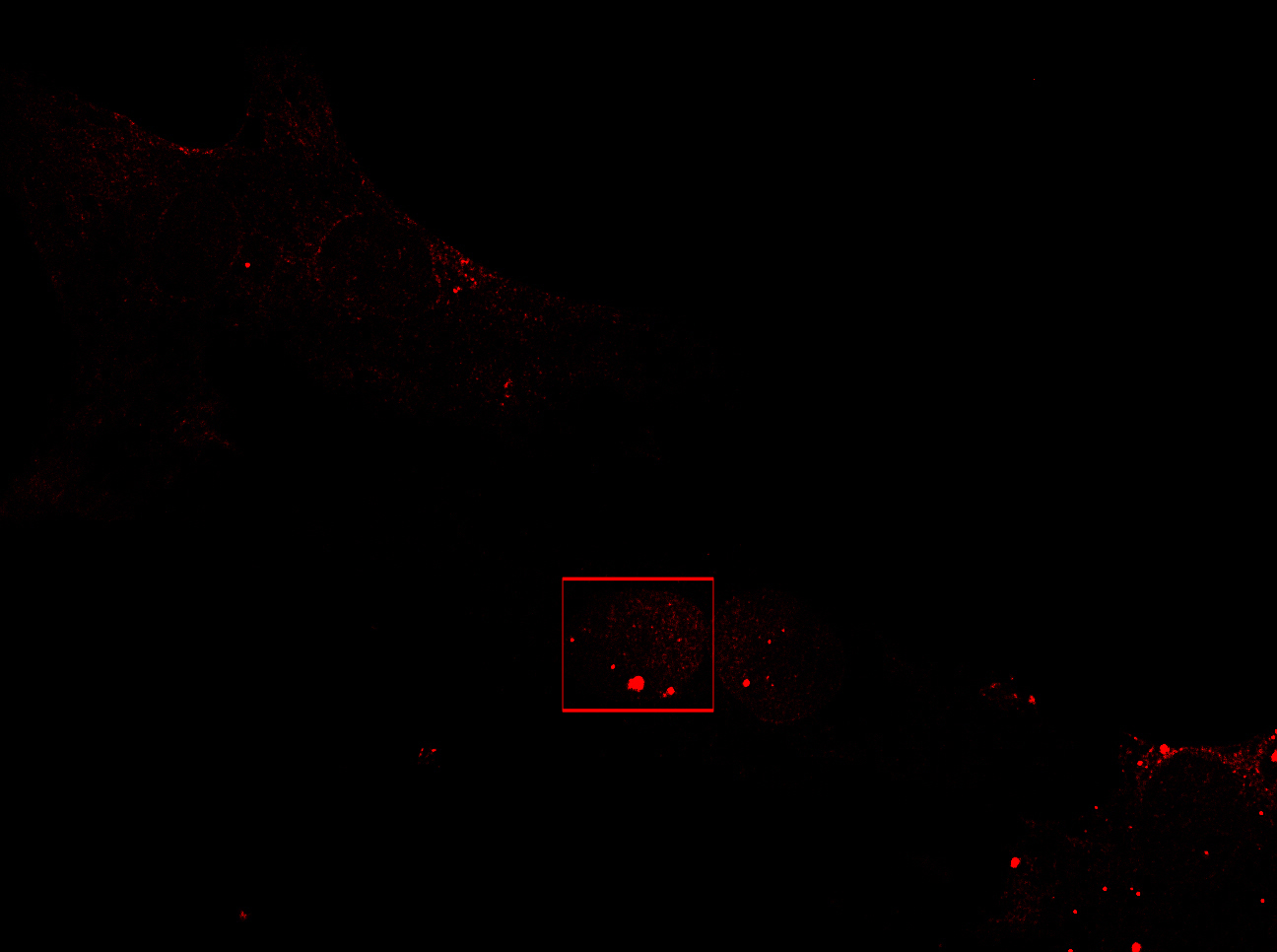

Supplement: Figure 5—source data 1. [file elife-54523-fig5-data1.zip › Figure 5/i/3_MacroH2A.1.jpg]

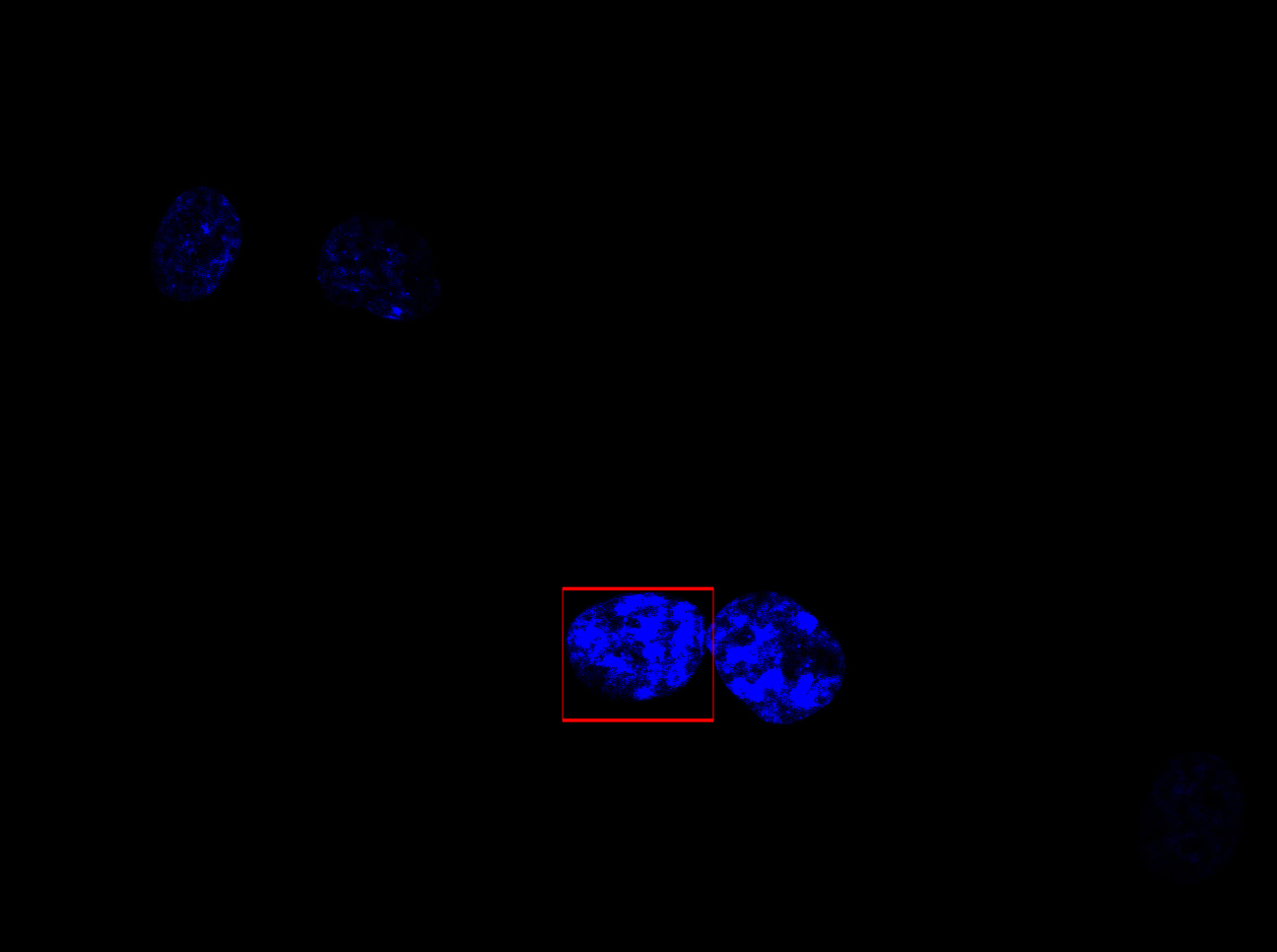

Supplement: Figure 5—source data 1. [file elife-54523-fig5-data1.zip › Figure 5/i/1_DAPI.jpg]

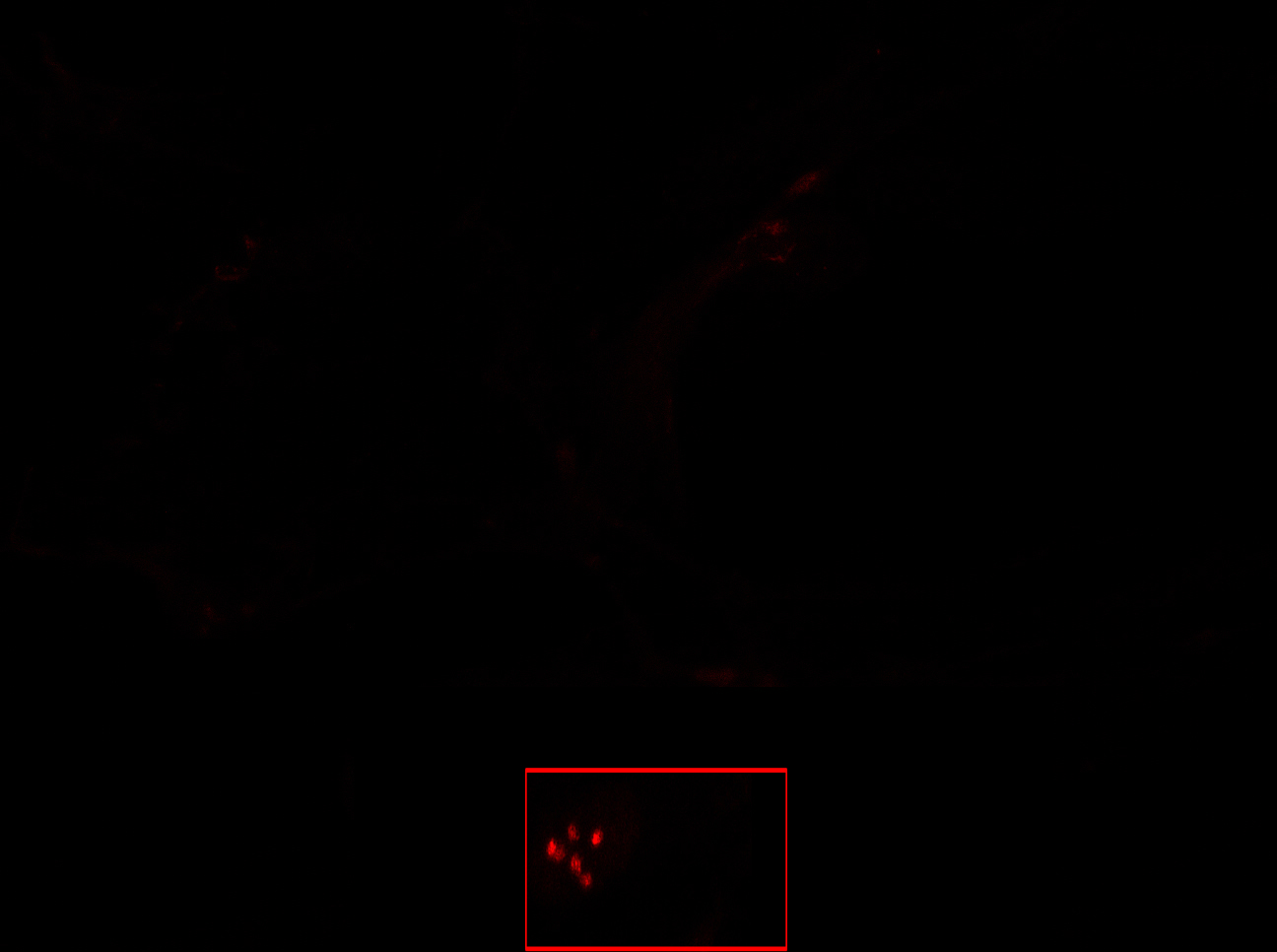

Supplement: Figure 5—source data 1. [file elife-54523-fig5-data1.zip › Figure 5/f/3_Ki67.jpg]

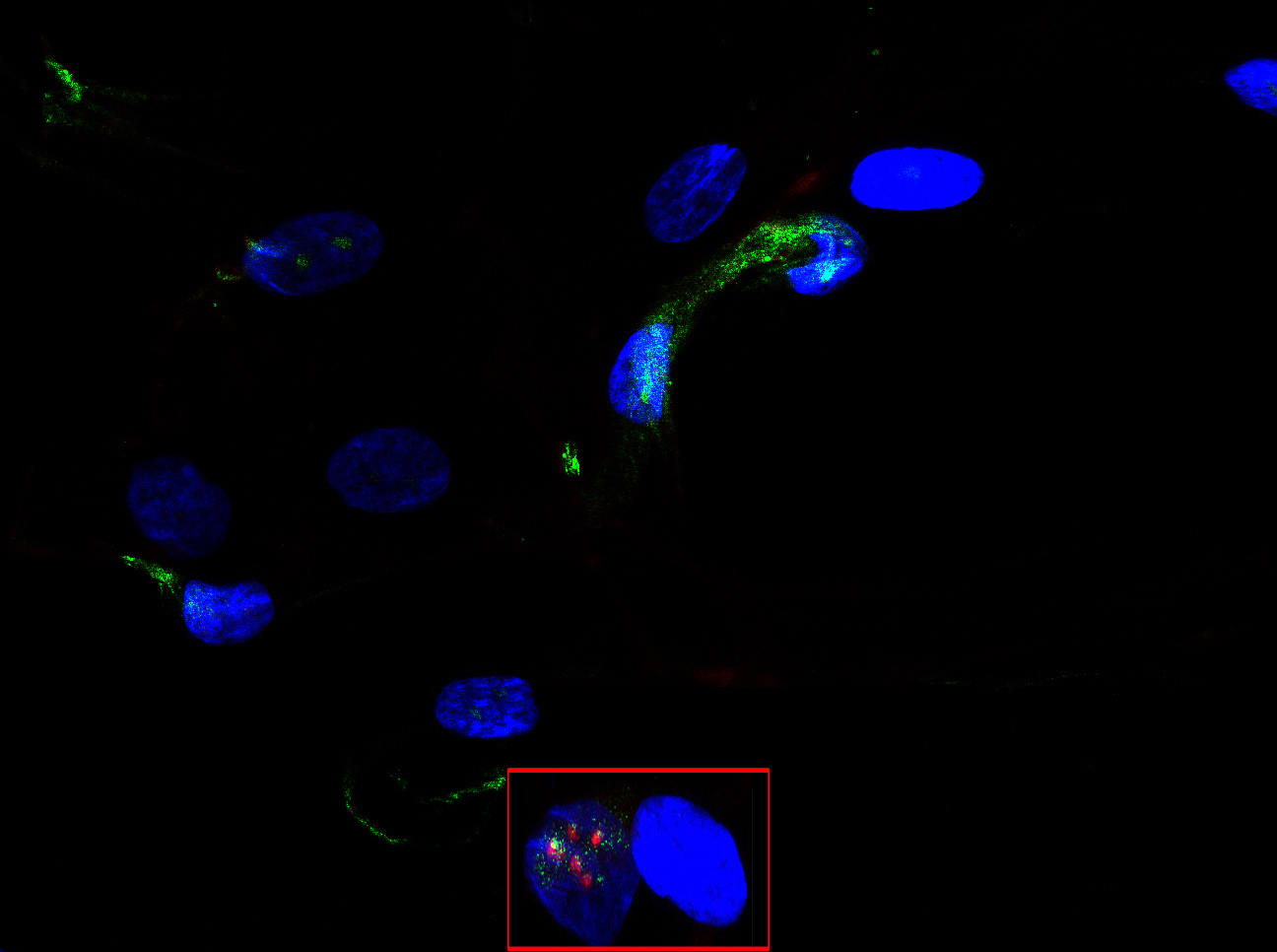

Supplement: Figure 5—source data 1. [file elife-54523-fig5-data1.zip › Figure 5/f/Merge.jpg]

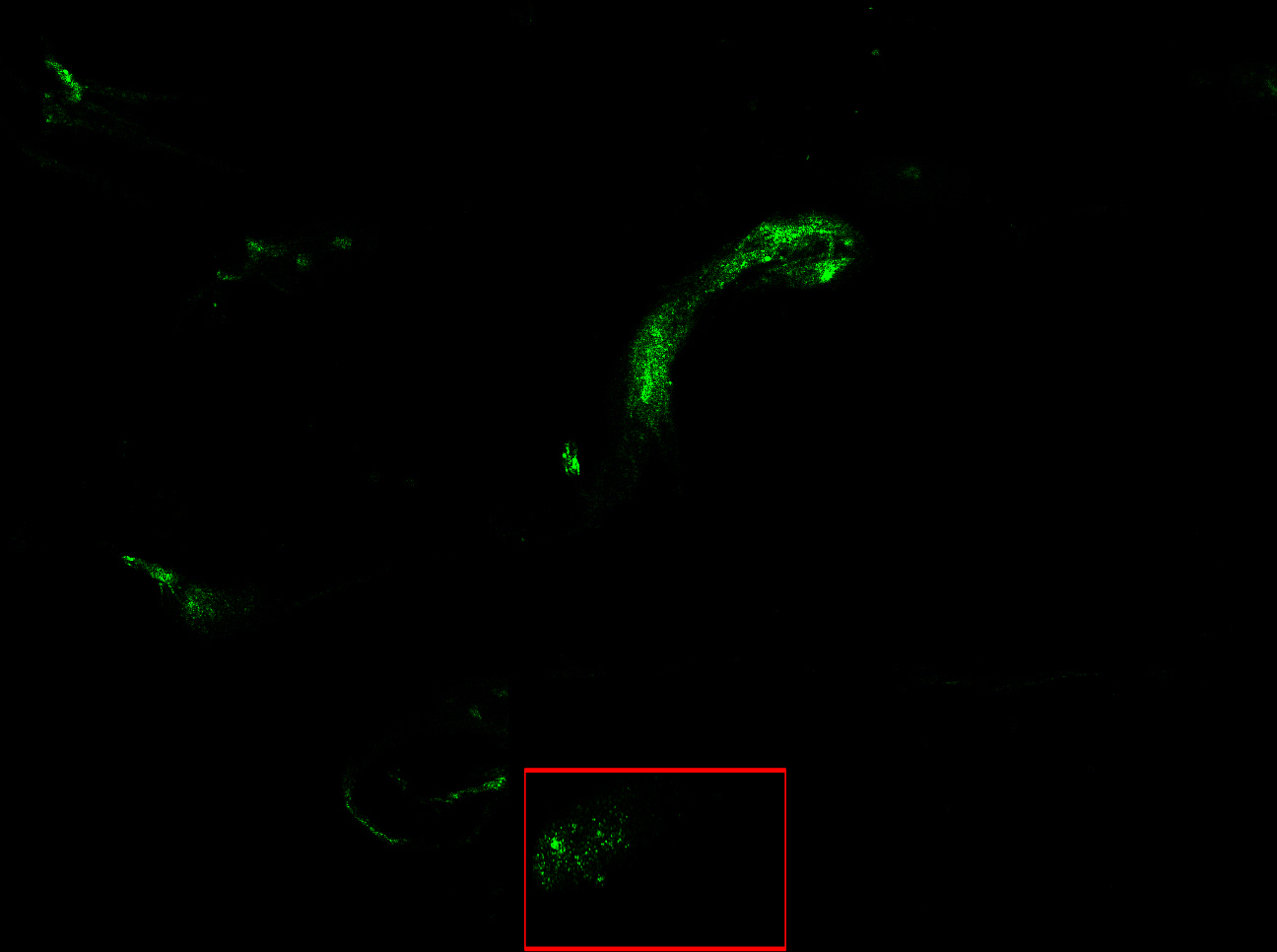

Supplement: Figure 5—source data 1. [file elife-54523-fig5-data1.zip › Figure 5/f/2_H1.2.jpg]

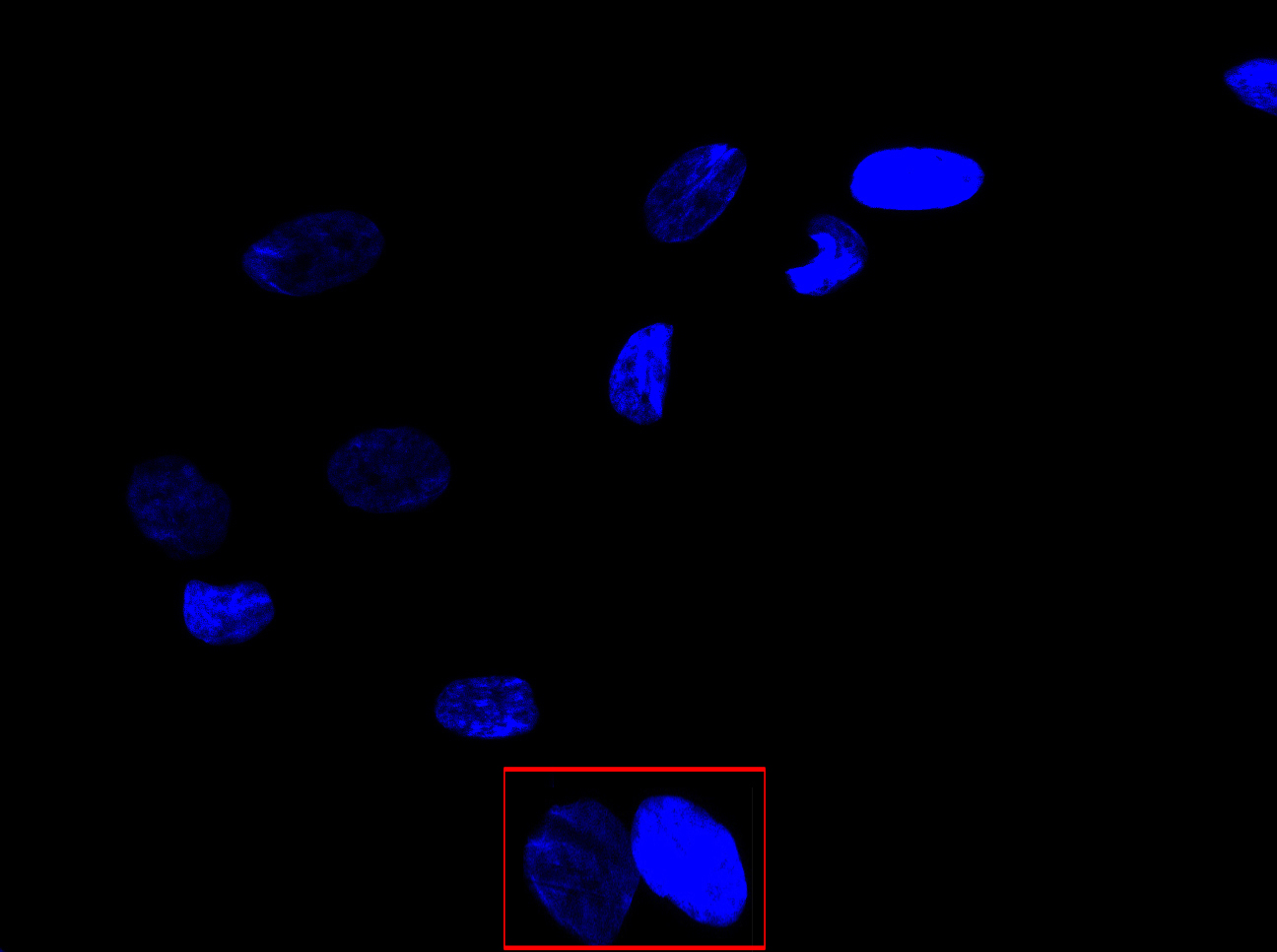

Supplement: Figure 5—source data 1. [file elife-54523-fig5-data1.zip › Figure 5/f/1_DAPI.jpg]

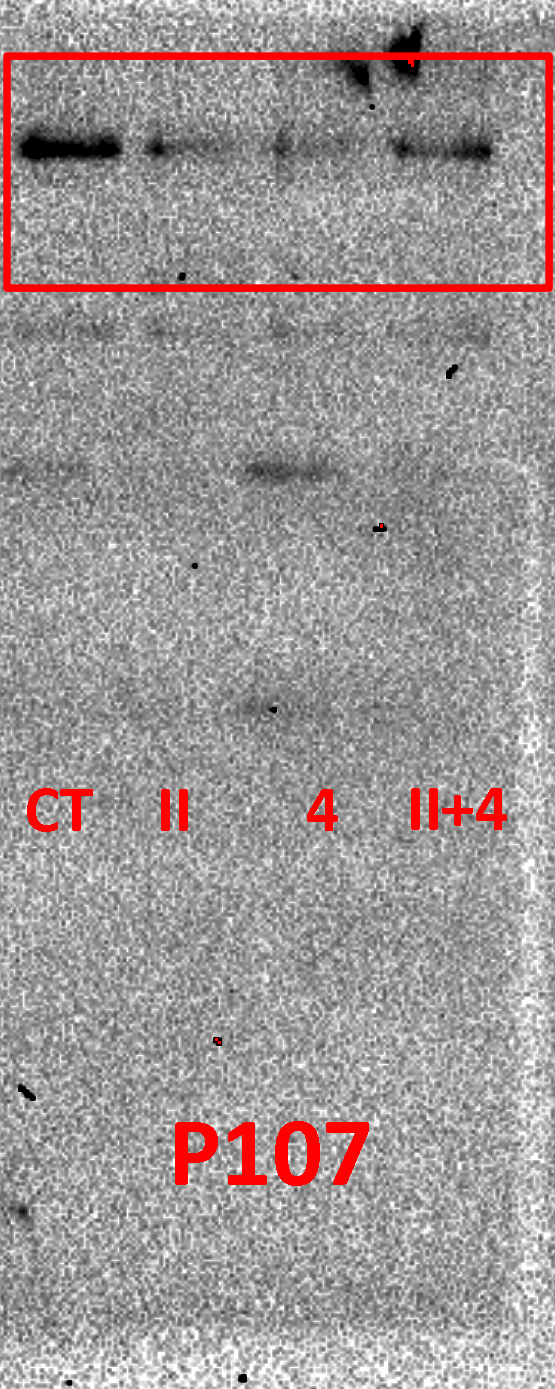

Supplement: Figure 5—source data 1. [file elife-54523-fig5-data1.zip › Figure 5/d/3.P107.jpg]

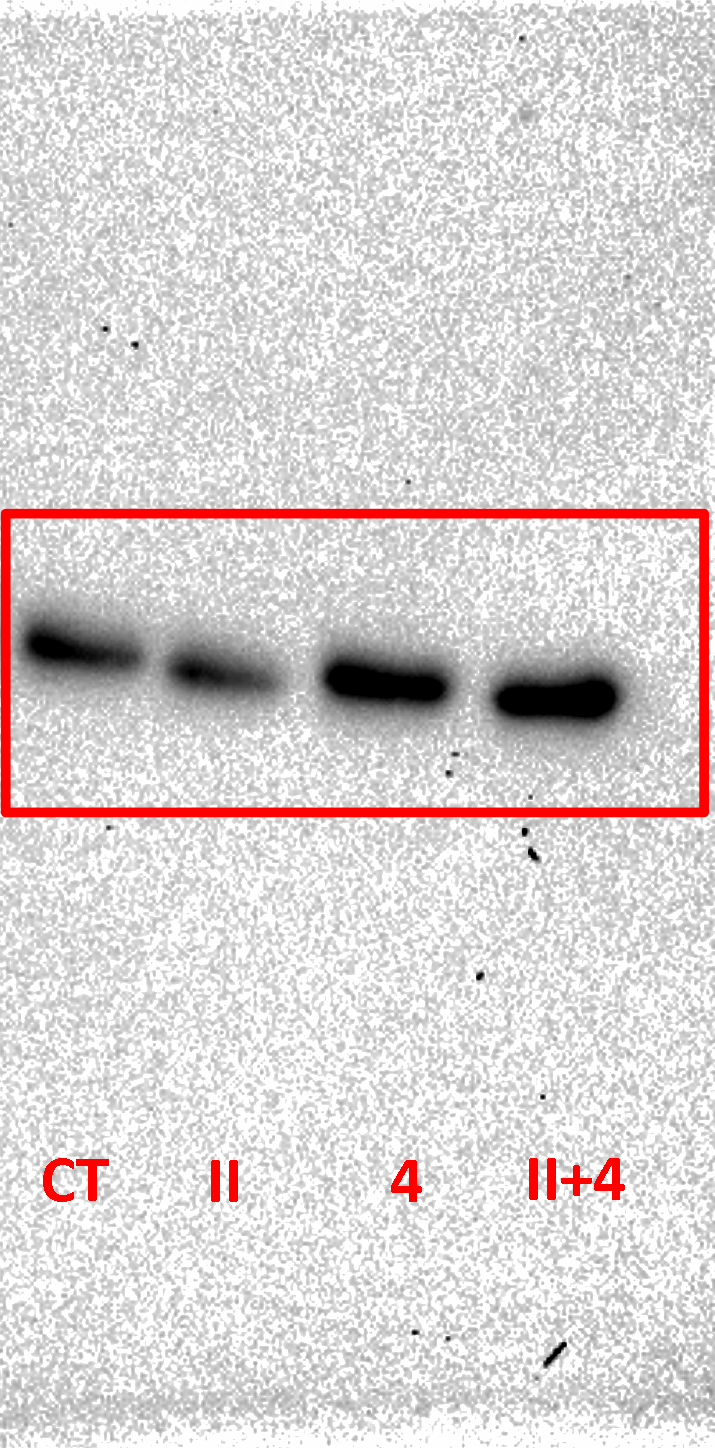

Supplement: Figure 5—source data 1. [file elife-54523-fig5-data1.zip › Figure 5/d/5.P27 .tif]

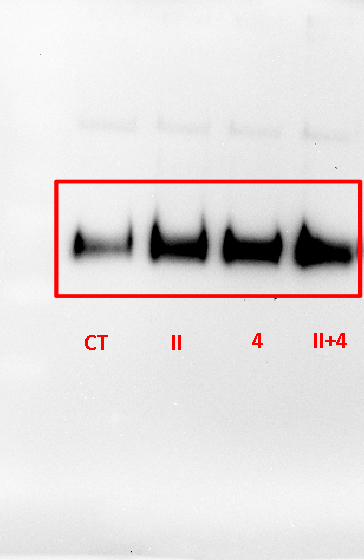

Supplement: Figure 5—source data 1. [file elife-54523-fig5-data1.zip › Figure 5/d/7.P16.jpg]

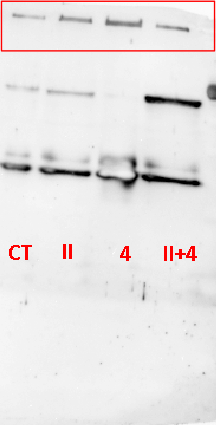

Supplement: Figure 5—source data 1. [file elife-54523-fig5-data1.zip › Figure 5/d/2.RB2.tif]

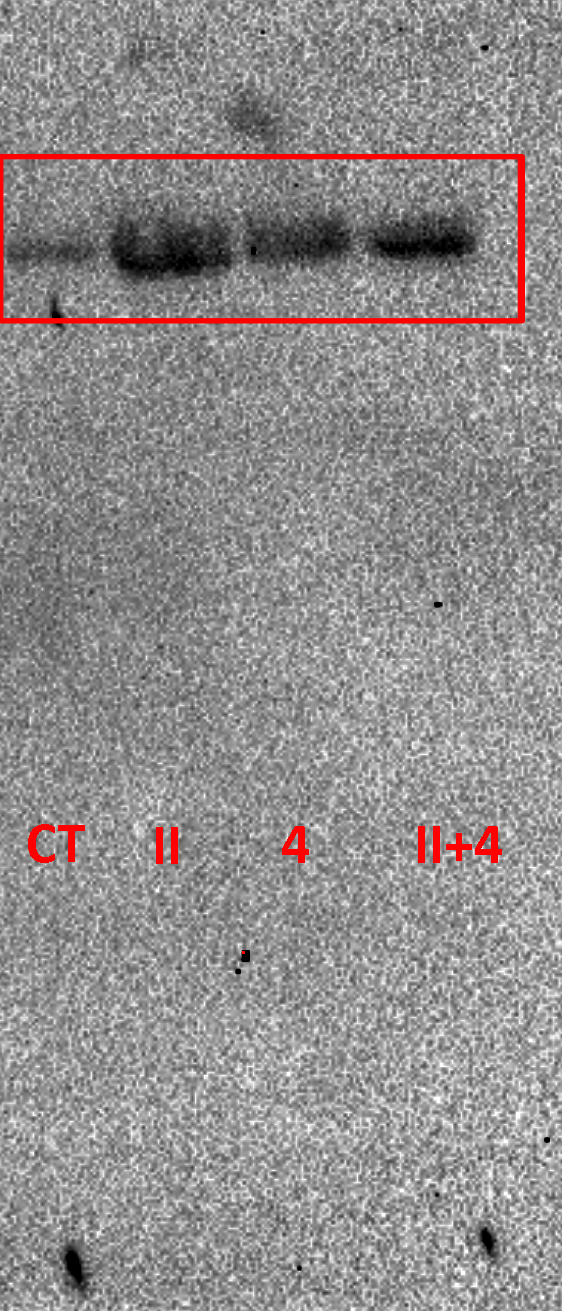

Supplement: Figure 5—source data 1. [file elife-54523-fig5-data1.zip › Figure 5/d/4.P53.jpg]

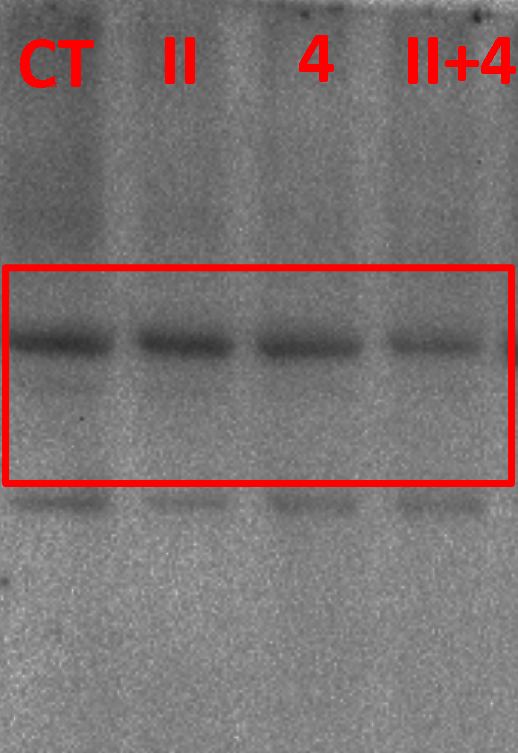

Supplement: Figure 5—source data 1. [file elife-54523-fig5-data1.zip › Figure 5/d/1.RB.jpg]

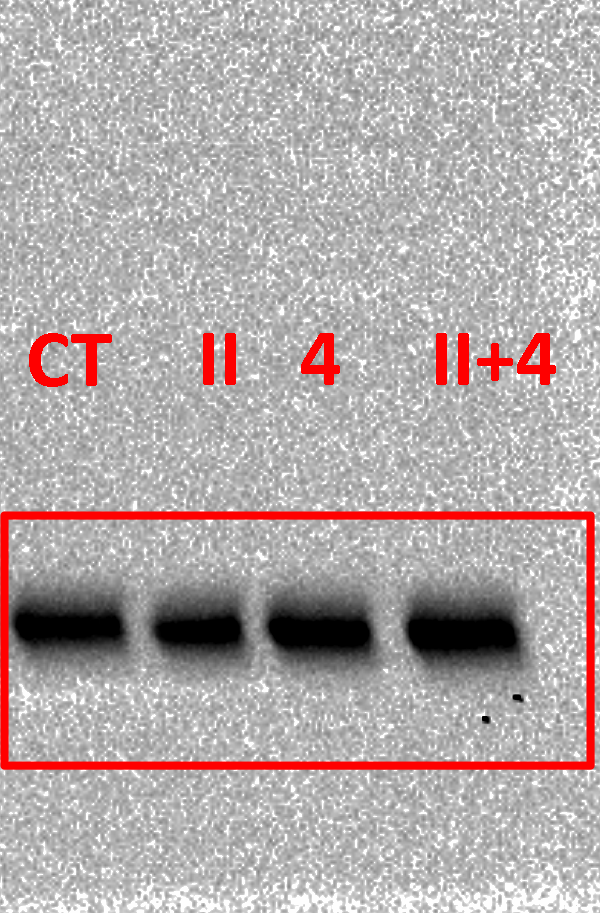

Supplement: Figure 5—source data 1. [file elife-54523-fig5-data1.zip › Figure 5/d/GAPDH.tif]

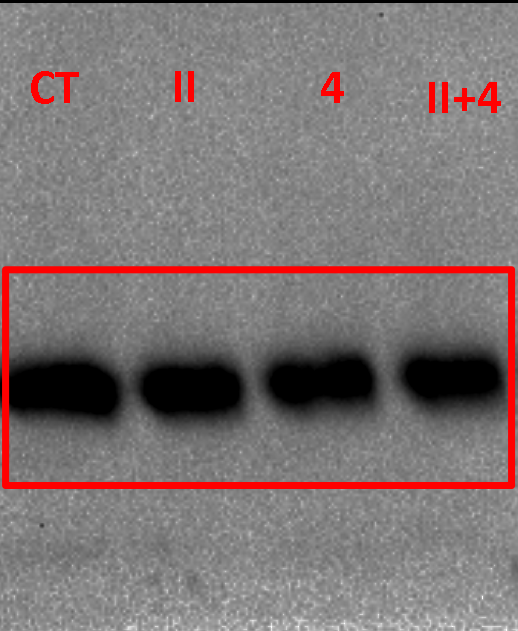

Supplement: Figure 5—source data 1. [file elife-54523-fig5-data1.zip › Figure 5/d/6.P21.tif]

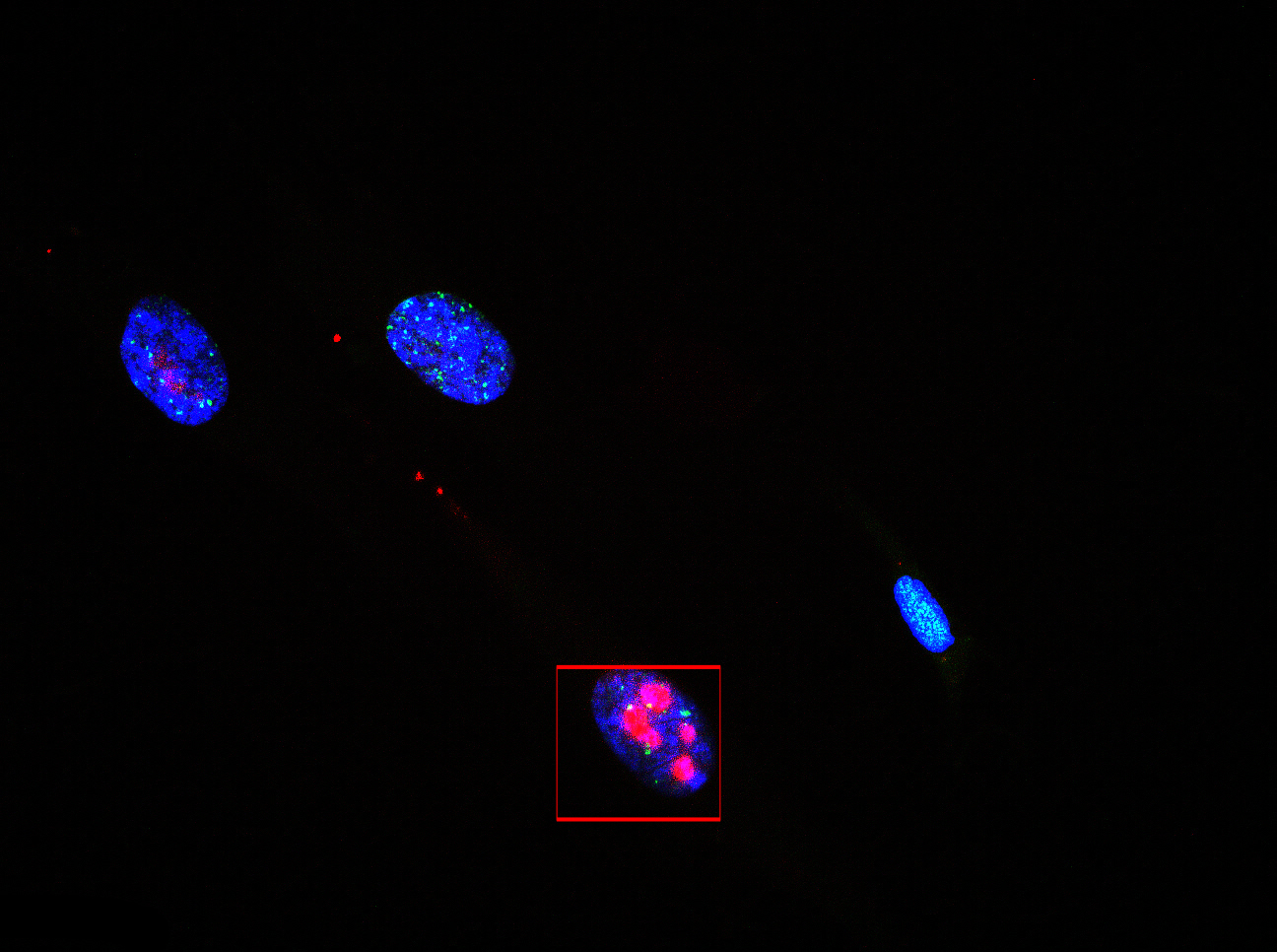

Supplement: Figure 5—source data 1. [file elife-54523-fig5-data1.zip › Figure 5/l/Merge.jpg]

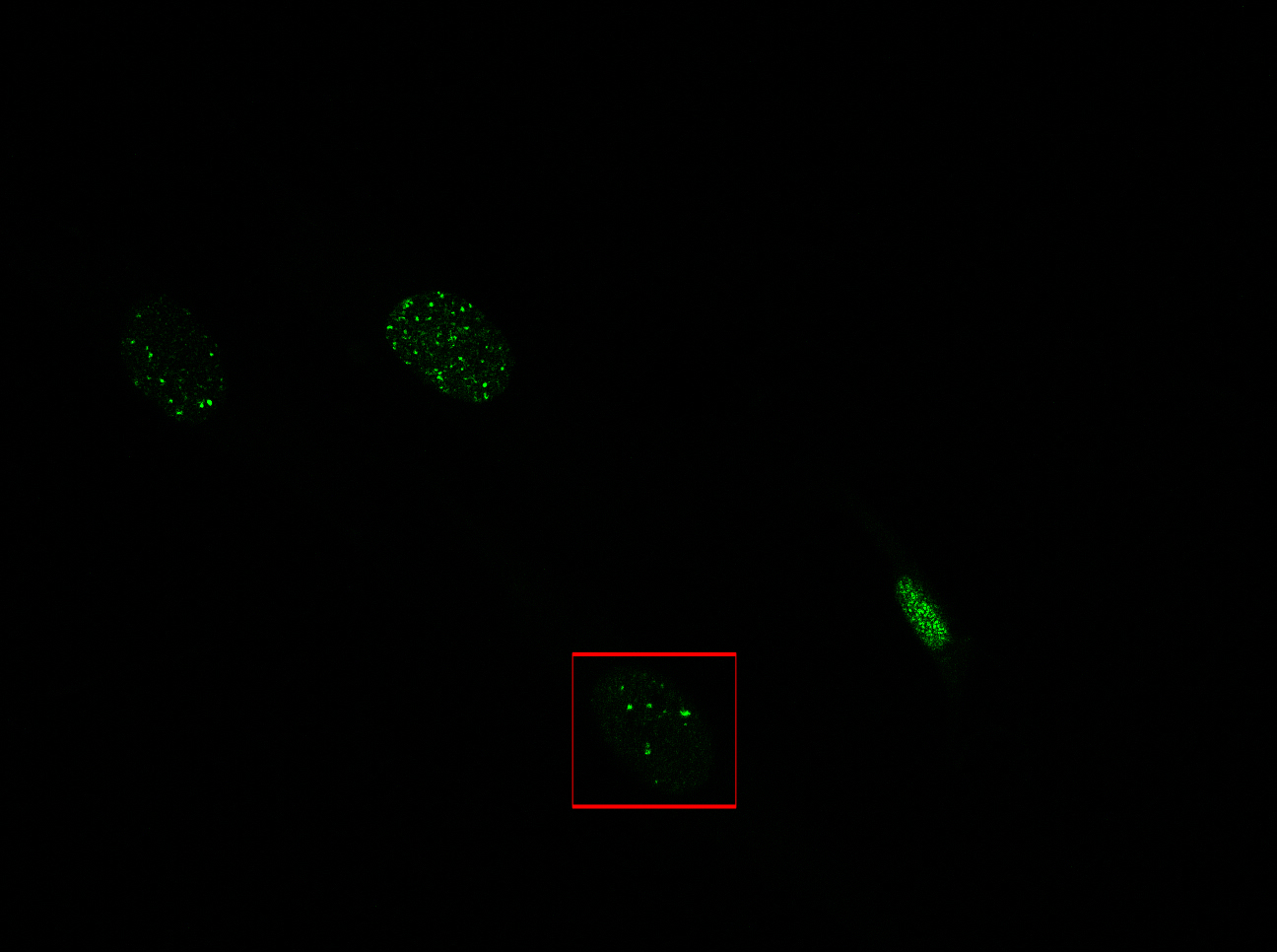

Supplement: Figure 5—source data 1. [file elife-54523-fig5-data1.zip › Figure 5/l/2_gH2AX.1.jpg]

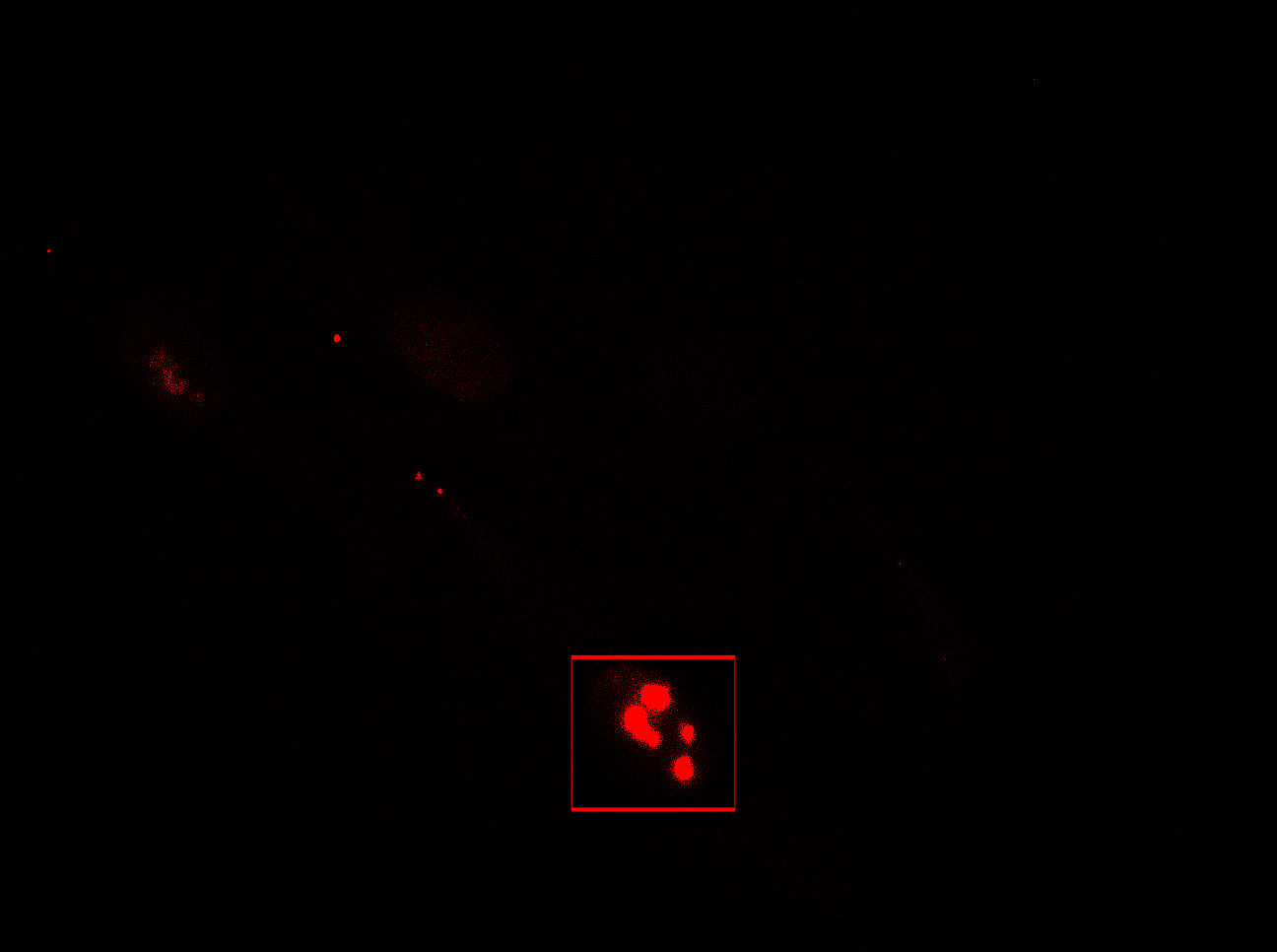

Supplement: Figure 5—source data 1. [file elife-54523-fig5-data1.zip › Figure 5/l/3_ATMjpg.jpg]

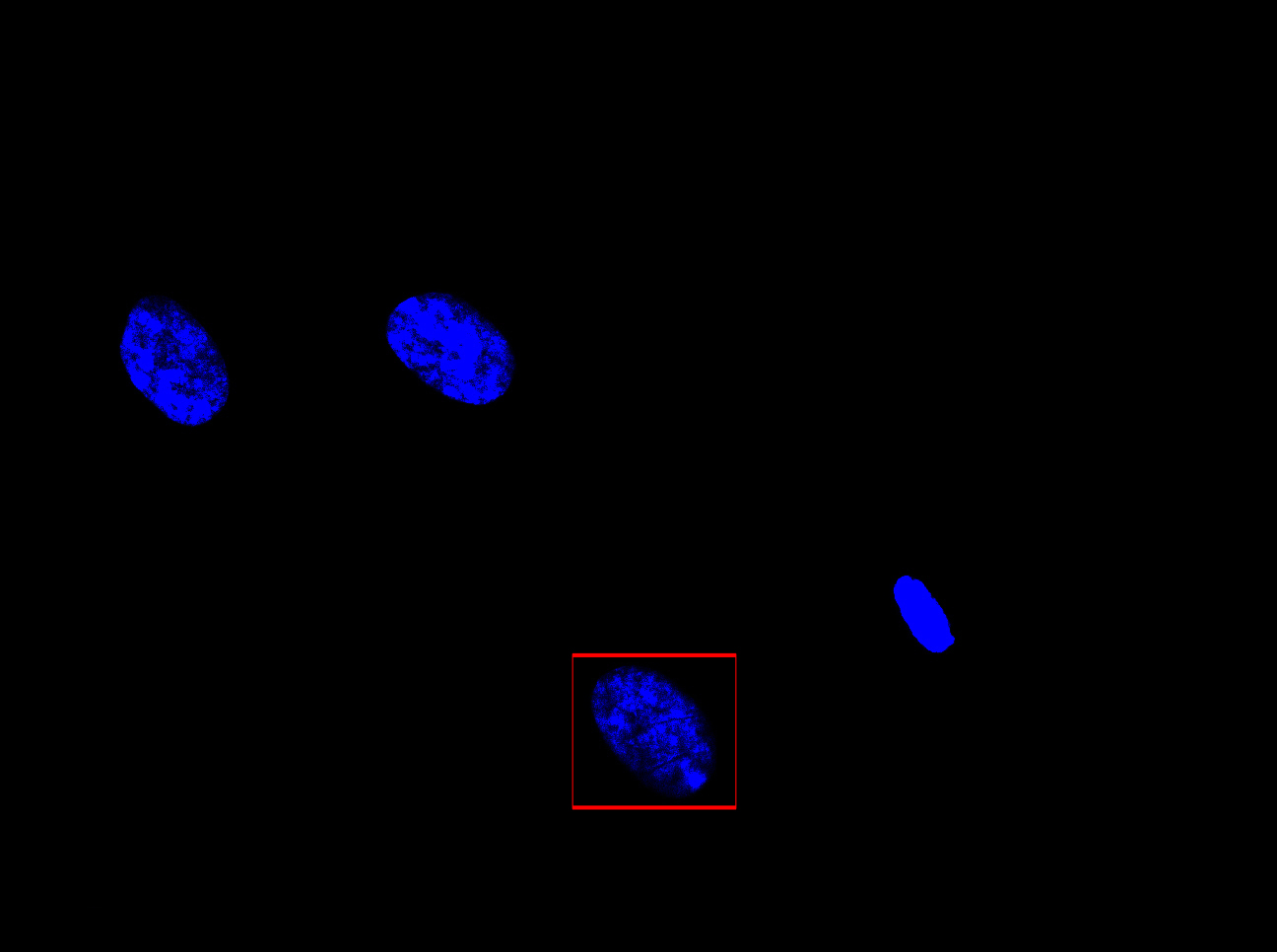

Supplement: Figure 5—source data 1. [file elife-54523-fig5-data1.zip › Figure 5/l/1_DAPI.jpg]

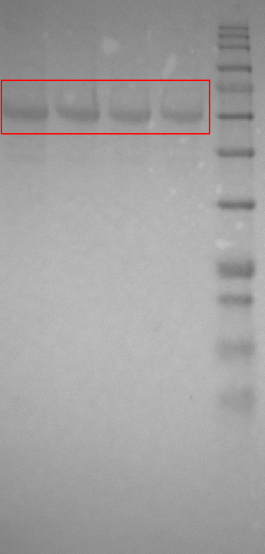

Supplement: Figure 7—source data 1. [file elife-54523-fig7-data1.zip › Figure 7/LC.jpg]

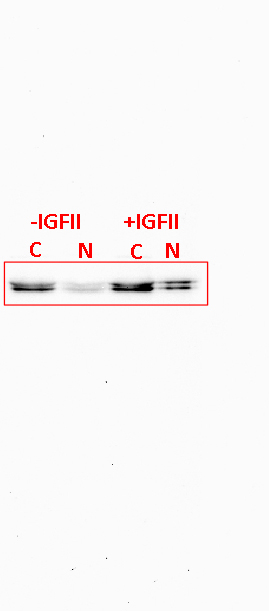

Supplement: Figure 7—source data 1. [file elife-54523-fig7-data1.zip › Figure 7/ERK nucleo cyto.jpg]

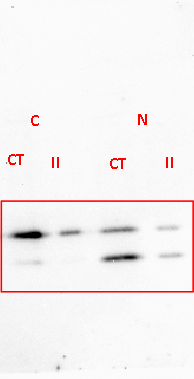

Supplement: Figure 7—figure supplement 1—source data 1. [file elife-54523-fig7-figsupp1-data1.zip › Figure 7ΓÇöfigure supplement 1/h.tif]

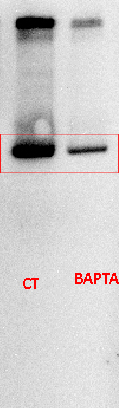

Supplement: Figure 7—figure supplement 1—source data 1. [file elife-54523-fig7-figsupp1-data1.zip › Figure 7ΓÇöfigure supplement 1/f/pCAMK2.jpg]

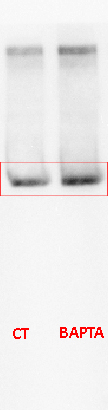

Supplement: Figure 7—figure supplement 1—source data 1. [file elife-54523-fig7-figsupp1-data1.zip › Figure 7ΓÇöfigure supplement 1/f/CAMK2 tot.jpg]

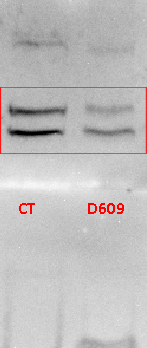

Supplement: Figure 7—figure supplement 1—source data 1. [file elife-54523-fig7-figsupp1-data1.zip › Figure 7ΓÇöfigure supplement 1/c/pP62.jpg]

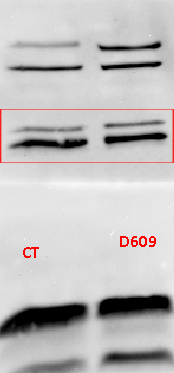

Supplement: Figure 7—figure supplement 1—source data 1. [file elife-54523-fig7-figsupp1-data1.zip › Figure 7ΓÇöfigure supplement 1/c/P62 tot.jpg]

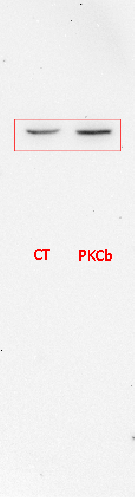

Supplement: Figure 7—figure supplement 1—source data 1. [file elife-54523-fig7-figsupp1-data1.zip › Figure 7ΓÇöfigure supplement 1/d/pP38.tif]

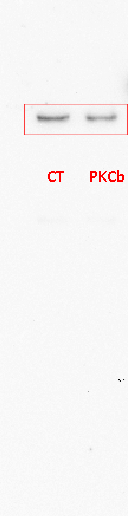

Supplement: Figure 7—figure supplement 1—source data 1. [file elife-54523-fig7-figsupp1-data1.zip › Figure 7ΓÇöfigure supplement 1/d/P38 tot.tif]

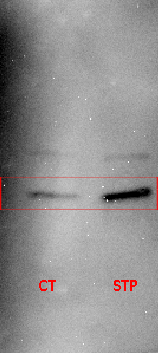

Supplement: Figure 7—figure supplement 1—source data 1. [file elife-54523-fig7-figsupp1-data1.zip › Figure 7ΓÇöfigure supplement 1/e/pP38.jpg]

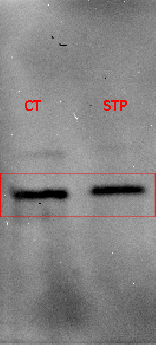

Supplement: Figure 7—figure supplement 1—source data 1. [file elife-54523-fig7-figsupp1-data1.zip › Figure 7ΓÇöfigure supplement 1/e/P38 tot.jpg]
