# Supplementary material for: Increase of circulating IGFBP-4 following genotoxic stress and its implication for senescence
Source: eLife. 2020 Mar 30;9:e54523. doi: 10.7554/eLife.54523 (PMC7136022; doi:10.7554/eLife.54523)
Supplement: Supplementary file 1. — For each figure the results from ANOVA and post hoc tests are provided in a separate file. We do not insert it here since it is a 40 pages document. [file elife-54523-supp1.docx]

**Supplementary File 1 Statistical Analysis**

# Results Figure 1a

ANOVA

ANOVA - IGFBP-4

Cases Sum of Squares Df Mean Square F p

| V1 | 6.858 | 5 | 1.372 | 33.82 < .001 |
| --- | --- | --- | --- | --- |
| Residual | 0.487 | 12 | 0.041 |  |

*Note.* Type III Sum of Squares

Post Hoc Tests

Post Hoc Comparisons - V1

Mean

|  | **Difference** |  | | | |
| --- | --- | --- | --- | --- | --- |
| 0h 6h | -0.433 | 0.164 | -2.635 | 0.162 |  |
| 24h | -1.467 | 0.164 | -8.920 | < .001 | *** |
| 48h | -1.233 | 0.164 | -7.501 | < .001 | *** |
| 72h | -1.467 | 0.164 | -8.920 | < .001 | *** |
| 144h | -1.700 | 0.164 | -10.339 | < .001 | *** |

**SE t p tukey Symbol**

# Results Figure 1b

ANOVA

ANOVA - β-galactosidase

Cases Sum of Squares Df Mean Square F p

| V1 | 4673.11 | 5 | 934.622 | 141.4 < .001 |
| --- | --- | --- | --- | --- |
| Residual | 79.33 | 12 | 6.611 |  |

*Note.* Type III Sum of Squares

Post Hoc Tests

Post Hoc Comparisons - V1

Mean

|  | | **Difference** |  | | | |
| --- | --- | --- | --- | --- | --- | --- |
| 0h Lung | 144h Lung | -17.000 | 2.099 | -8.098 | < .001 | *** |
| 0h Heart | 144h Heart | -11.000 | 2.099 | -5.240 | 0.002 | ** |
| 0h Kidney | 144h Kidney | -37.333 | 2.099 | -17.783 | < .001 | *** |

SE t p _tukey_ Symbol

# Results Figure 1*c*

ANOVA

ANOVA - β-galactosidase

Cases Sum of Squares df Mean Square F p

| V1 | 181.50 | 1 | 181.500 | 19.45 0.012 |
| --- | --- | --- | --- | --- |
| Residual | 37.33 | 4 | 9.333 |  |

*Note.* Type III Sum of Squares

Post Hoc Tests

Post Hoc Comparisons - V1

Mean Difference SE t p _tukey_ Symbol

0h MSC 144h MSC -11.00 2.494 -4.410 0.012 *

# Results Figure 1d

ANOVA

ANOVA - CCK-8

Cases Sum of Squares df Mean Square F p

| V1 | 1.472 | 7 | 0.210 | 38.21 < .001 |
| --- | --- | --- | --- | --- |
| Residual | 0.088 | 16 | 0.006 |  |

*Note.* Type III Sum of Squares

Post Hoc Tests

Post Hoc Comparisons - V1

Mean Difference SE t p _tukey_ Symbol

| 0h-1d 144h-1d | 0.010 | 0.061 0.165 1.000 |  |
| --- | --- | --- | --- |
| 0h-2d 144h-2d | 0.049 | 0.061 0.814 0.990 |  |
| 0h-3d 144h-3d | 0.259 | 0.061 4.281 0.011 | * |
| 0h-4d 144h-4d | 0.373 | 0.061 6.163 < .001 | *** |

# Results Figure 1e

ANOVA

ANOVA - protein level

Cases Sum of Squares df Mean Square F p

V1 4.170e +6 9 463316 28.18 < .001

Residual 328810 20 16441

*Note.* Type III Sum of Squares

Post Hoc Tests

Post Hoc Comparisons - V1

Mean Difference SE t p _tukey_ Symbol

| rb 0h rb 144h | -384.667 | 104.7 -3.674 0.038 | * |
| --- | --- | --- | --- |
| p53 0h p53 144h | -826.667 | 104.7 -7.896 < .001 | *** |
| p27 0h p27 144h | -576.000 | 104.7 -5.502 < .001 | *** |
| p21 0h p21 144h | -304.333 | 104.7 -2.907 0.038 | * |
| p16 0h p16 144h | -502.667 | 104.7 -4.801 0.003 | ** |

# Results Figure 1f

ANOVA

ANOVA - CFU

Cases Sum of Squares df Mean Square F p

| V1 | 13728 | 1 | 13728.2 | 15.90 0.016 |
| --- | --- | --- | --- | --- |
| Residual | 3455 | 4 | 863.7 |  |

*Note.* Type III Sum of Squares

Post Hoc Tests

Post Hoc Comparisons - V1

Mean Difference SE t p _tukey_ Symbol

CTRL IGFBP-4 95.67 24.00 3.987 0.016 *

# Results Figure 1g

ANOVA

ANOVA - β-galactosidase

Cases Sum of Squares df Mean Square F p

| V1 | 11365.8 | 5 | 2273.16 | 81.35 < .001 |
| --- | --- | --- | --- | --- |
| Residual | 335.3 | 12 | 27.94 |  |

*Note.* Type III Sum of Squares

Post Hoc Tests

Post Hoc Comparisons - V1

Mean Difference SE t p _tukey_ Symbol

| CTRL Lung IGFBP-4 Lung | -25.170 | 4.383 -5.742 0.010 | ** |
| --- | --- | --- | --- |
| CTRL Heart IGFBP-4 Heart | -14.000 | 3.073 -4.556 0.020 | * |
| CTRL Kidney IGFBP-4 Kidney | -37.000 | 7.601 -4.868 0.017 | * |

# Results Figure 1h

ANOVA

ANOVA - β-galactosidase

Cases Sum of Squares df Mean Square F p

| V1 | 308.17 | 1 | 308.167 | 57.78 0.002 |
| --- | --- | --- | --- | --- |
| Residual | 21.33 | 4 | 5.333 |  |

*Note.* Type III Sum of Squares

Post Hoc Tests

Post Hoc Comparisons - V1

Mean Difference SE t p _tukey_ Symbol

CTRL IGFBP-4 -14.33 1.886 -7.601 0.002 **

ANOVA - CCK-8

Cases Sum of Squares df Mean Square F p

| V1 | 3.042 | 7 | 0.435 | 24.26 < .001 |
| --- | --- | --- | --- | --- |
| Residual | 0.287 | 16 | 0.018 |  |

*Note.* Type III Sum of Squares

Post Hoc Tests

Post Hoc Comparisons - V1

Mean Difference SE t p _tukey_ Symbol

CTRL 1d IGFBP-4 1d -0.069 0.102 -0.678 0.996

| CTRL 2d IGFBP-4 2d | 0.147 | 0.109 | 1.345 | 0.867 |  |
| --- | --- | --- | --- | --- | --- |
| CTRL 3d IGFBP-4 3d | 0.336 | 0.109 | 3.072 | 0.101 |  |
| CTRL 4d IGFBP-4 4d | 0.649 | 0.122 | 5.310 | 0.001 | ** |

# Results Figure 1j

ANOVA

ANOVA - protein level

Cases Sum of Squares df Mean Square F p

V1 5.707e +6 9 634072 43.37 < .001

Residual 292408 20 14620

*Note.* Type III Sum of Squares

Post Hoc Tests

Post Hoc Comparisons - V1

Mean Difference SE t p _tukey_ Symbol

| rb CTRL rb IGFBP-4 | -736.33 | 98.73 -7.458 < .001 | | | *** |
| --- | --- | --- | --- | --- | --- |
| p53 CTRL p53 IGFBP-4 | -1083.00 | 98.73 -10.970 < .001 | | | *** |
| p27 CTRL p27 IGFBP-4 | -361.67 | 98.73 | -3.663 | 0.039 | * |
| p21 CTRL p21 IGFBP-4 | -419.00 | 98.73 | -4.244 | 0.011 | * |
| p16 CTRL p16 IGFBP-4 | -406.33 | 98.73 | -4.116 | 0.015 | * |

ANOVA - IGFBP-4

Cases Sum of Squares df Mean Square F p

| V1 | 32.087 | 19 | 1.689 | 30.99 < .001 |
| --- | --- | --- | --- | --- |
| Residual | 2.180 | 40 | 0.054 |  |

*Note.* Type III Sum of Squares

Post Hoc Tests

Post Hoc Comparisons - V1

Mean Difference SE t p _tukey_ Symbol

| 1B | 1A | 1.300 | 0.191 6.820 0.002 | ** |
| --- | --- | --- | --- | --- |
| 2B | 2A | 0.600 | 0.191 3.148 0.035 | * |
| 3B | 3A | 0.933 | 0.191 4.896 0.011 | * |
| 4B | 4A | 0.967 | 0.191 5.071 0.005 | ** |
| 5B | 5A | 0.867 | 0.191 4.547 0.004 | ** |
| 6B | 6A | 0.733 | 0.191 3.847 0.002 | ** |
| 7B | 7A | 0.833 | 0.191 4.372 0.035 | ** |
| 8B | 8A | 0.967 | 0.191 5.071 0.014 | ** |
| 9B | 9A | 1.200 | 0.191 6.295 <.001 | *** |
| 10B | 10A | 0.800 | 0.191 4.197 0.014 | * |

ANOVA - β-galactosidase

Cases Sum of Squares df Mean Square F p

| V1 | 1888.44 | 5 | 377.689 | 123.6 < .001 |
| --- | --- | --- | --- | --- |
| Residual | 36.67 | 12 | 3.056 |  |

*Note.* Type III Sum of Squares

Post Hoc Tests

Post Hoc Comparisons - V1

Mean Difference SE t p _tukey_ Symbol

| CTRL CTRL + PXB | -0.333 | 1.427 -0.234 1.000 | | |  |
| --- | --- | --- | --- | --- | --- |
| H_2_O_2_ | -22.667 | 1.427 -15.881 < .001 | | | *** |
| H_2_O_2_ +PXB | -20.667 | 1.427 -14.480 < .001 | | | *** |
| PGE2 | -0.667 | 1.427 | -0.467 | 0.997 | |
| PGE2 +PXB | 1.000 | 1.427 | 0.701 | 0.978 | |
| H_2_O_2_ H_2_O_2_ +PXB | 2.000 | 1.427 | 1.401 | 0.726 | |
| PGE2 PGE2 +PXB | 1.667 | 1.427 | 1.168 | 0.843 | |

Post Hoc Comparisons - V1

Mean Difference SE t p _tukey_ Symbol

| CTRL H_2_O_2_ | -0.593 | 0.065 | -9.139 | < .001 | *** |
| --- | --- | --- | --- | --- | --- |
| PGE2 | -0.993 | 0.065 | -15.301 | < .001 | *** |
| CTRL + PXB | 0.350 | 0.065 | 5.391 | 0.002 | ** |
| H_2_O_2_ + PXB | 0.317 | 0.065 | 4.878 | 0.004 | ** |
| PGE2 + PXB | 0.433 | 0.065 | 6.675 | < .001 | *** |
| H_2_O_2_ H_2_O_2_ + PXB | 0.910 | 0.065 | 14.017 | < .001 | ### |
| PGE2 PGE2 + PXB | 1.427 | 0.065 | 21.975 | < .001 | §§§ |

# Results Figure 4b

ANOVA

ANOVA - IGFBP

Cases Sum of Squares df Mean Square F p

| V1 | 4.772 | 4 | 1.193 | 107.2 < .001 |
| --- | --- | --- | --- | --- |
| Residual | 0.111 | 10 | 0.011 |  |

*Note.* Type III Sum of Squares

Post Hoc Tests

Post Hoc Comparisons - V1

Mean Difference SE t p _tukey_ Symbol

CTRL H_2_O_2_ -0.953 0.101 -9.409 < .001 ***

| H_2_O_2_ H_2_O_2_ + siCTRL | 0.043 | 0.101 0.428 0.992 |  |
| --- | --- | --- | --- |
| H_2_O_2_ + siEP2 | 1.330 | 0.101 13.126 < .001 | ### |
| H_2_O_2_ + siGαs | 1.237 | 0.101 12.205 < .001 | ### |

Post Hoc Comparisons - V1

Mean Difference SE t p _tukey_ Symbol

CT H_2_O_2_ -1.203 0.160 -7.499 < .001 ***

H_2_O_2_ H_2_O_2_ + BSD 1.230 0.160 7.665 < .001 ###

| H_2_O_2_ + LY | -0.413 | 0.160 -2.576 0.177 |
| --- | --- | --- |
| H_2_O_2_ + PP1 | -0.170 | 0.160 -1.059 0.888 |
| H_2_O_2_ + U0 | -0.490 | 0.160 -3.053 0.083 |

# Results Figure 5a

ANOVA

ANOVA - β-galactosidase

Cases Sum of Squares df Mean Square F p

| V1 | 1335.90 | 6 | 222.651 | 79.25 < .001 |
| --- | --- | --- | --- | --- |
| Residual | 39.33 | 14 | 2.810 |  |

*Note.* Type III Sum of Squares

Post Hoc Tests

Post Hoc Comparisons - V1

Mean Difference SE t p _tukey_ Symbol

| CTRL IGF-I | 1.000 | 1.369 | 0.731 | 0.988 |  |
| --- | --- | --- | --- | --- | --- |
| IGF-I + IGF-IR | -1.000 | 1.369 | -0.731 | 0.988 |  |
| IGF-I + IGF-IIR | 1.000 | 1.369 | 0.731 | 0.988 |  |
| IGF-II | -10.000 | 1.369 | -7.307 | < .001 | *** |
| IGF-II + IGF-IR | -22.333 | 1.369 | -16.319 | < .001 | *** |
| IGF-II +IGF-IIR | -2.333 | 1.369 | -1.705 | 0.624 |  |
| IGF-I IGF-I + IGF-IR | -2.000 | 1.369 | -1.461 | 0.762 |  |
| IGF-I + IGF-IIR -2.665e -15 1.369 -1.947e -15 1.000 | | | | | |
| IGF-II IGF-II + IGF-IR | -12.333 | 1.369 | -9.012 | < .001 | |
| IGF-II +IGF-IIR | 7.667 | 1.369 | 5.602 | 0.001 | |

ANOVA - β-galactosidase

Cases Sum of Squares df Mean Square F p

| V1 | 597.58 | 3 | 199.194 | 40.51 < .001 |
| --- | --- | --- | --- | --- |
| Residual | 39.33 | 8 | 4.917 |  |

*Note.* Type III Sum of Squares

Post Hoc Tests

Post Hoc Comparisons - V1

Mean Difference SE t p _tukey_ Symbol

| CTRL | IGF-II | -9.667 | 1.810 | -5.339 | 0.003 | ** |
| --- | --- | --- | --- | --- | --- | --- |
|  | IGFBP-4 | -7.000 | 1.810 | -3.866 | 0.020 | * |

IGF-II + IGFBP-4 -19.667 1.810 -10.863 < .001 ***

# Results Figure 5*c*

ANOVA

ANOVA - cell cycle

Cases Sum of Squares df Mean Square F p

| V1 | 24481.7 | 11 | 2225.606 | 388.9 < .001 |
| --- | --- | --- | --- | --- |
| Residual | 137.3 | 24 | 5.722 |  |

*Note.* Type III Sum of Squares

Post Hoc Tests

Post Hoc Comparisons - V1

Mean Difference SE t p _tukey_ Symbol

| G1 CTRL | G1 IGF-II | -9.000 | 1.953 -4.608 0.005 | | | ** |
| --- | --- | --- | --- | --- | --- | --- |
|  | G1 IGFBP-4 | -5.667 | 1.953 -2.901 0.201 | | |  |
|  | G1 IGF-II + IGFBP-4 | -11.667 | 1.953 -5.973 < .001 | | | *** |
| S CTRL | S IGF-II | 12.333 | 1.953 | 6.315 | < .001 | *** |
|  | S IGFBP-4 | 12.000 | 1.953 | 6.144 | < .001 | *** |
|  | S IGF-II + IGFBP-4 | 13.667 | 1.953 | 6.997 | < .001 | *** |
| G2/M CTRL | G2/M IGF-II | -3.333 | 1.953 | -1.707 | 0.849 |  |
| G2/M IGFBP-4 | | 1.000 | 1.953 0.512 1.000 | | | |
| G2/M IGF-II + IGFBP-4 | | -2.000 | 1.953 -1.024 0.995 | | | |

# Results Figure 5e

ANOVA

ANOVA - protein level

Cases Sum of Squares df Mean Square F p

| V1 | 39.890 | 27 | 1.477 | 52.18 < .001 |
| --- | --- | --- | --- | --- |
| Residual | 1.586 | 56 | 0.028 |  |

*Note.* Type III Sum of Squares

Post Hoc Tests

Post Hoc Comparisons - V1

Mean Difference SE t p _tukey_ Symbol

| RB CTRL | RB IGF-II | 0.143 | 0.137 | 1.043 | 1.000 |  |
| --- | --- | --- | --- | --- | --- | --- |
|  | RB IFGBP-4 | 0.847 | 0.137 | 6.162 | < .001 | *** |
|  | RB IGF-II + IGFBP-4 | 1.287 | 0.137 | 9.365 | < .001 | *** |
| RB2 CTRL | RB2 IGF-II | -1.257 | 0.137 | -9.146 | < .001 | *** |
| RB2 IFGBP-4 -1.707 0.137 -12.422 < .001 *** | | | | | | |
|  | RB2 IGF-II + IGFBP-4 | -0.933 | 0.137 | -6.793 | < .001 | *** |
| P107 CTRL | P107 IGF-II | 2.117 | 0.137 | 15.406 | < .001 | *** |
|  | P107 IFGBP-4 | 2.150 | 0.137 | 15.648 | < .001 | *** |
|  | P107 IGF-II + IGFBP-4 | 1.567 | 0.137 | 11.403 | < .001 | *** |
| P53 CTRL | P53 IGF-II | -0.433 | 0.137 | -3.154 | 0.023 | * |
|  | P53 IFGBP-4 | -0.243 | 0.137 | -1.771 | 0.990 |  |
|  | P53 IGF-II + IGFBP-4 | -0.567 | 0.137 | -4.124 | 0.028 | * |
| P27 CTRL | P27 IGF-II | -0.220 | 0.137 | -1.601 | 0.997 |  |
|  | P27 IFGBP-4 | -0.553 | 0.137 | -4.027 | 0.037 | * |
|  | P27 IGF-II + IGFBP-4 | -0.610 | 0.137 | -4.440 | 0.011 | * |
| P21 CTRL | P21 IGF-II | -0.090 | 0.137 | -0.655 | 1.000 |  |
|  | P21 IFGBP-4 | 0.027 | 0.137 | 0.194 | 1.000 |  |
|  | P21 IGF-II + IGFBP-4 | 0.070 | 0.137 | 0.509 | 1.000 |  |
| P16 CTRL | P16 IGF-II | -1.077 | 0.137 | -7.836 | < .001 | *** |
|  | P16 IFGBP-4 | -1.100 | 0.137 | -8.006 | < .001 | *** |

P16 IGF-II + IGFBP-4 -1.580 0.137 -11.500 < .001 ***

# Results Figure 5g

ANOVA

ANOVA - H1.2

Cases Sum of Squares df Mean Square F p

| V1 | 152.830 | 3 | 50.943 | 44.85 < .001 |
| --- | --- | --- | --- | --- |
| Residual | 9.088 | 8 | 1.136 |  |

*Note.* Type III Sum of Squares

Post Hoc Tests

Post Hoc Comparisons - V1

Mean Difference SE t p _tukey_ Symbol

| CTRL | IGF-II | 6.100 | 0.870 7.010 < .001 | *** |
| --- | --- | --- | --- | --- |
|  | IFGBP-4 | 8.727 | 0.870 10.028 < .001 | *** |
|  | IGF-II + IGFBP-4 | 8.747 | 0.870 10.051 < .001 | *** |

# Results Figure 5h

ANOVA

ANOVA - Ki-67

Cases Sum of Squares df Mean Square F p

| V1 | 1702.9 | 3 | 567.64 | 25.80 < .001 |
| --- | --- | --- | --- | --- |
| Residual | 176.0 | 8 | 22.00 |  |

*Note.* Type III Sum of Squares

Post Hoc Tests

Post Hoc Comparisons - V1

Mean Difference SE t p _tukey_ Symbol

| CTRL | IGF-II | 17.333 | 3.830 4.526 0.008 | ** |
| --- | --- | --- | --- | --- |
|  | IFGBP-4 | 16.000 | 3.830 4.178 0.013 | * |
|  | IGF-II + IGFBP-4 | 33.667 | 3.830 8.791 < .001 | *** |

# Results Figure 5j

ANOVA

ANOVA - HP1

Cases Sum of Squares df Mean Square F p

| V1 | 28.833 | 3 | 9.611 | 62.34 < .001 |
| --- | --- | --- | --- | --- |
| Residual | 1.233 | 8 | 0.154 |  |

*Note.* Type III Sum of Squares

Post Hoc Tests

Post Hoc Comparisons - V1

Mean Difference SE t p _tukey_ Symbol

| CTRL | IGF-II | -3.567 | 0.321 -11.125 < .001 | *** |
| --- | --- | --- | --- | --- |
|  | IFGBP-4 | -3.967 | 0.321 -12.373 < .001 | *** |
|  | IGF-II + IGFBP-4 | -2.200 | 0.321 -6.862 < .001 | *** |

# Results Figure 5k

ANOVA

ANOVA - Macro H2A.1

Cases Sum of Squares df Mean Square F p

| V1 | 0.313 | 3 | 0.104 | 1.694 0.245 |
| --- | --- | --- | --- | --- |
| Residual | 0.493 | 8 | 0.062 |  |

*Note.* Type III Sum of Squares

Post Hoc Tests

Post Hoc Comparisons - V1

Mean Difference SE t p _tukey_ Symbol

| CTRL | IGF-II | 0.367 | 0.203 1.808 0.336 |
| --- | --- | --- | --- |
|  | IFGBP-4 | 0.400 | 0.203 1.973 0.274 |
|  | IGF-II + IGFBP-4 | 0.167 | 0.203 0.822 0.843 |

ANOVA - y-H2AX

Cases Sum of Squares df Mean Square F p

| V1 | 0.004 | 3 | 0.001 | 13.00 0.002 |
| --- | --- | --- | --- | --- |
| Residual | 8.000e -4 | 8 | 1.000e -4 |  |

*Note.* Type III Sum of Squares

Post Hoc Tests

Post Hoc Comparisons - V1

Mean Difference SE t p _tukey_ Symbol

| CTRL | IGF-II | -0.030 | 0.008 -3.674 0.026 | * |
| --- | --- | --- | --- | --- |
|  | IFGBP-4 | 0.020 | 0.008 2.449 0.145 |  |
|  | IGF-II + IGFBP-4 | -0.010 | 0.008 -1.225 0.630 |  |

# Results Figure 5n

ANOVA

ANOVA – ATM

Cases Sum of Squares df Mean Square F p

| V1 | 1.583 | 3 | 0.528 | 0.373 0.775 |
| --- | --- | --- | --- | --- |
| Residual | 11.333 | 8 | 1.417 |  |

*Note.* Type III Sum of Squares

Post Hoc Tests

Post Hoc Comparisons - V1

|  | | **Mean Difference** | **SE** | **t** | **p _tukey_ Symbol** |
| --- | --- | --- | --- | --- | --- |
| CTRL | IGF-II | 0.333 | 0.972 | 0.343 | 0.985 |
|  | IFGBP-4 | -0.667 | 0.972 | -0.686 | 0.900 |

IGF-II + IGFBP-4 1.721e -15 0.972 1.771e -15 1.000

ANOVA - IGF-II

Cases Sum of Squares df Mean Square F p

V1 1.577e +7 19 829781 175.7 < .001

Residual 188867 40 4722

*Note.* Type III Sum of Squares

Post Hoc Tests

Post Hoc Comparisons - V1

Mean Difference SE t p _tukey_ Symbol

0 IGF-II 0 IGFBP-4 -2.387e -12 56.11 -4.255e -14 1.000

| 10 IGF-II | 10 IGFBP-4 | -103.333 | 56.11 | -1.842 | 0.938 |  |
| --- | --- | --- | --- | --- | --- | --- |
| 20 IGF-II | 20 IGFBP-4 | -300.000 | 56.11 | -5.347 | < .001 | *** |
| 30 IGF-II | 30 IGFBP-4 | -560.000 | 56.11 | -9.981 | < .001 | *** |
| 60 IGF-II | 60 IGFBP-4 | -690.000 | 56.11 | -12.298 | < .001 | *** |
| 90 IGF-II | 90 IGFBP-4 | -670.000 | 56.11 | -11.942 | < .001 | *** |
| 120 IGF-II | 120 IGFBP-4 | -666.667 | 56.11 | -11.882 | < .001 | *** |
| 240 IGF-II | 240 IGFBP-4 | -816.667 | 56.11 | -14.556 | < .001 | *** |
| 480 IGF-II | 480 IGFBP-4 | -906.667 | 56.11 | -16.160 | < .001 | *** |
| 1080 IGF-II | 1080 IGFBP-4 | -1190.000 | 56.11 | -21.210 | < .001 | *** |

ANOVA – β-galactosidase

Cases Sum of Squares df Mean Square F p

| V1 | 569.17 | 7 | 81.310 | 18.95 < .001 |
| --- | --- | --- | --- | --- |
| Residual | 68.67 | 16 | 4.292 |  |

*Note.* Type III Sum of Squares

Post Hoc Tests

Post Hoc Comparisons - V1

|  | **Mean Difference** | **SE** | **t** | **p _tukey_** | **Symbol** |
| --- | --- | --- | --- | --- | --- |
| CTRL IGF-II | -4.667 | 1.691 | -2.759 | 0.174 |  |
| IGFBP-4 | -7.000 | 1.691 | -4.138 | 0.014 | ** |
| IGF-II + IGFBP-4 | -16.333 | 1.691 | -9.656 | < .001 | *** |
| PAPP-A | -1.333 | 1.691 | -0.788 | 0.991 |  |
| IGF-II + PAPP-A | -3.333 | 1.691 | -1.971 | 0.528 |  |
| IGFBP-4 + PAPP-A | -1.000 | 1.691 | -0.591 | 0.999 |  |
| IGF-II + IGFBP-4+ PAPP-A | -4.333 | 1.691 | -2.562 | 0.238 |  |
| PAPP-A IGF-II + PAPP-A | 2.000 | 1.691 | 1.182 | 0.926 |  |
| IGFBP-4 + PAPP-A | -0.333 | 1.691 | -0.197 | 1.000 |  |
| IGF-II + IGFBP-4 + PAPP-A | 3.000 | 1.691 | 1.774 | 0.645 |  |

ANOVA - β-galactosidase

Cases Sum of Squares df Mean Square F p

| V1 | 499.87 | 9 | 55.541 | 20.07 < .001 |
| --- | --- | --- | --- | --- |
| Residual | 55.33 | 20 | 2.767 |  |

*Note.* Type III Sum of Squares

Post Hoc Tests

Post Hoc Comparisons - V1

Mean Difference SE t p _tukey_ Symbol

CTRL IGF-II -10.000 1.358 -7.363 < .001 ***

| IGF-II | IGF-II + PTX | 3.000 | 1.358 | 2.209 | 0.482 |  |
| --- | --- | --- | --- | --- | --- | --- |
|  | IGF-II + STP | 1.667 | 1.358 | 1.227 | 0.959 |  |
|  | IGF-II + YM | 11.000 | 1.358 | 8.100 | < .001 | ### |
|  | IGF-II + D609 | 10.667 | 1.358 | 7.854 | < .001 | ### |
|  | IGF-II + PKCi | 11.000 | 1.358 | 8.100 | < .001 | ### |
|  | IGF-II + BAPTA-AM | 5.333 | 1.358 | 3.927 | 0.022 | # |
|  | IGF-II + U0 | 11.000 | 1.358 | 8.100 | < .001 | ### |
|  | IGF-II + CPZ | 7.000 | 1.358 | 5.154 | 0.002 | ## |

# Results Supp File 1b

ANOVA

ANOVA - β-galactosidase

Cases Sum of Squares df Mean Square F p

| V1 | 2226.3 | 5 | 445.26 | 24.14 < .001 |
| --- | --- | --- | --- | --- |
| Residual | 221.3 | 12 | 18.44 |  |

*Note.* Type III Sum of Squares

Post Hoc Tests

Post Hoc Comparisons - V1

Mean Difference SE t p _tukey_ Symbol

CTRL Doxo -21.000 3.507 -5.989 < .001 ***

| H_2_O_2_ | -24.333 | 3.507 -6.939 < .001 | *** |
| --- | --- | --- | --- |
| IRH | -22.667 | 3.507 -6.464 < .001 | *** |
| IRL | -5.667 | 3.507 -1.616 0.604 |  |
| Rep | -32.000 | 3.507 -9.126 < .001 | *** |

# Results Supp File 1*c*

ANOVA

ANOVA - IGFBP-4

Cases Sum of Squares df Mean Square F p

| V1 | 3.078 | 5 | 0.616 | 22.61 < .001 |
| --- | --- | --- | --- | --- |
| Residual | 0.327 | 12 | 0.027 |  |

*Note.* Type III Sum of Squares

Post Hoc Tests

Post Hoc Comparisons - V1

Mean Difference SE t p _tukey_ Symbol

| CTRL Doxo | -0.467 | 0.135 -3.464 0.042 | * |
| --- | --- | --- | --- |
| H_2_O_2_ | -1.133 | 0.135 -8.413 < .001 | *** |
| IRH | -1.100 | 0.135 -8.165 < .001 | *** |
| IRL | -0.333 | 0.135 -2.474 0.206 |  |
| Rep | -0.833 | 0.135 -6.186 < .001 | *** |

# Results Supp File 1d

ANOVA

ANOVA - IGFBP-4

Cases Sum of Squares df Mean Square F p

| V1 | 5.383 | 3 | 1.794 | 30.76 < .001 |
| --- | --- | --- | --- | --- |
| Residual | 0.467 | 8 | 0.058 |  |

*Note.* Type III Sum of Squares

Post Hoc Tests

Post Hoc Comparisons - V1

Mean Difference SE t p _tukey_ Symbol

| 0h 24h | -0.333 | 0.197 -1.690 0.387 |  |
| --- | --- | --- | --- |
| 48h | -1.533 | 0.197 -7.775 < .001 | *** |
| 72h | -1.433 | 0.197 -7.268 < .001 | *** |

ANOVA - PGE2

Cases Sum of Squares df Mean Square F p

| V1 | 7.008 | 9 | 0.779 | 135.4 < .001 |
| --- | --- | --- | --- | --- |
| Residual | 0.115 | 20 | 0.006 |  |

*Note.* Type III Sum of Squares

Post Hoc Tests

Post Hoc Comparisons - V1

Mean Difference SE t p _tukey_ Symbol

| 0 min 10 min | -0.433 | 0.062 -6.999 < .001 | *** |
| --- | --- | --- | --- |
| 20 min | -1.000 | 0.062 -16.151 < .001 | *** |
| 30 min | -1.683 | 0.062 -27.188 < .001 | *** |
| 40 min | -1.533 | 0.062 -24.766 < .001 | *** |
| 50 min | -1.233 | 0.062 -19.920 < .001 | *** |
| 60 min | -1.067 | 0.062 -17.228 < .001 | *** |
| 90 min | -0.967 | 0.062 -15.613 < .001 | *** |
| 180 min | -0.750 | 0.062 -12.114 < .001 | *** |
| 360 min | -0.550 | 0.062 -8.883 < .001 | *** |

# Results Supp File *2*b

ANOVA

ANOVA - EP2 mRNA

Cases Sum of Squares df Mean Square F P

| V1 | 0.534 | 1 | 0.534 | 44.01 0.003 |
| --- | --- | --- | --- | --- |
| Residual | 0.049 | 4 | 0.012 |  |

*Note.* Type III Sum of Squares

Post Hoc Tests

Post Hoc Comparisons - V1

Mean Difference SE t p _tukey_ Symbol

siCTRL siEP2 0.597 0.090 6.634 0.003 **

ANOVA - Gas mRNA

Cases Sum of Squares df Mean Square F p

| V1 | 3.375 | 1 | 3.375 | 144.6 < .001 |
| --- | --- | --- | --- | --- |
| Residual | 0.093 | 4 | 0.023 |  |

*Note.* Type III Sum of Squares

Post Hoc Tests

Post Hoc Comparisons - V1

Mean Difference SE t p _tukey_ Symbol

siCTRL siGαs 1.500 0.125 12.03 < .001 ***

# Results Supp File 3a

ANOVA

ANOVA – cAMP

Cases Sum of Squares df Mean Square F p

| V1 | 3.010e +6 | 1 | 3.010e +6 | 118.4 < .001 |
| --- | --- | --- | --- | --- |
| Residual | 101667 | 4 | 25417 |  |

*Note.* Type III Sum of Squares

Post Hoc Tests

Post Hoc Comparisons - V1

Mean Difference SE T p _tukey_ Symbol

CTRL PTX 1417 130.2 10.88 < .001 ***

ANOVA - PLCb

Cases Sum of Squares df Mean Square F p

| V1 | 0.522 | 1 | 0.522 | 91.07 < .001 |
| --- | --- | --- | --- | --- |
| Residual | 0.023 | 4 | 0.006 |  |

*Note.* Type III Sum of Squares

Post Hoc Tests

Post Hoc Comparisons - V1

Mean Difference SE t p _tukey_ Symbol

CTRL YM 0.590 0.062 9.543 < .001 ***
