# Supplementary material for: Increase of circulating IGFBP-4 following genotoxic stress and its implication for senescence
Source: eLife. 2020 Mar 30;9:e54523. doi: 10.7554/eLife.54523 (PMC7136022; doi:10.7554/eLife.54523)

Power size calculation for IGFBP-4 increase following X ray irradiation in mice (Figure 1a). Based on previous experiments, we assumed that basal serum value of IGFBP-4 in control mice was 100 ± 20. Data were expressed in arbitrary units. We expected at least a 50% increase in IGFBP-4 value following irradiation.


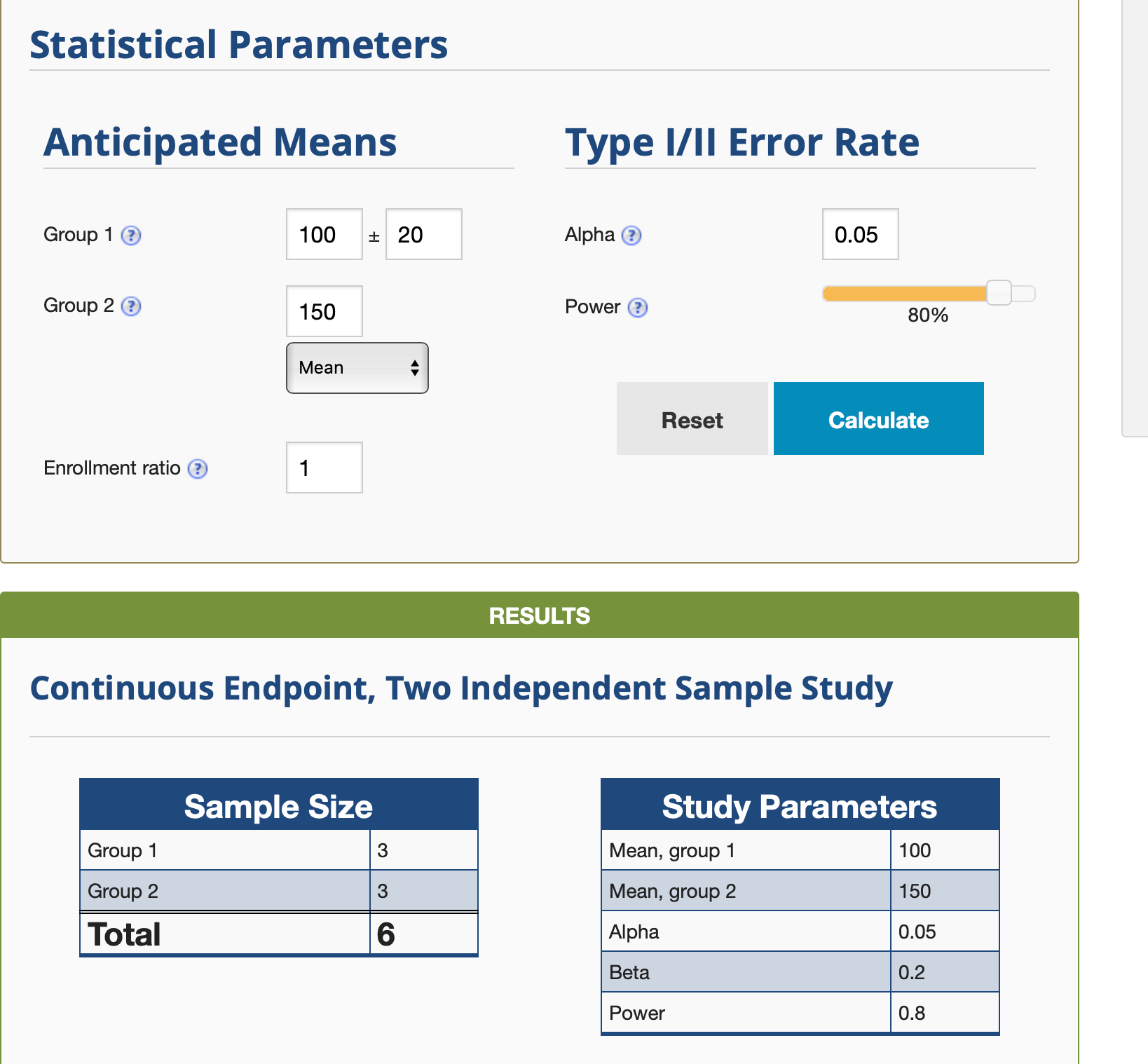


For other data on mice shown in figure 1 we used the same approach.

Power size calculation for paired data obtained from patients before and after CT scan (Figure 2) Based on previous experiments, we assumed that expected mean of the paired differences in serum IGFBP-4 before and after CT scan was 1.02 (arbitrary units) and the expected standard

deviation of the paired differences was 0.4.


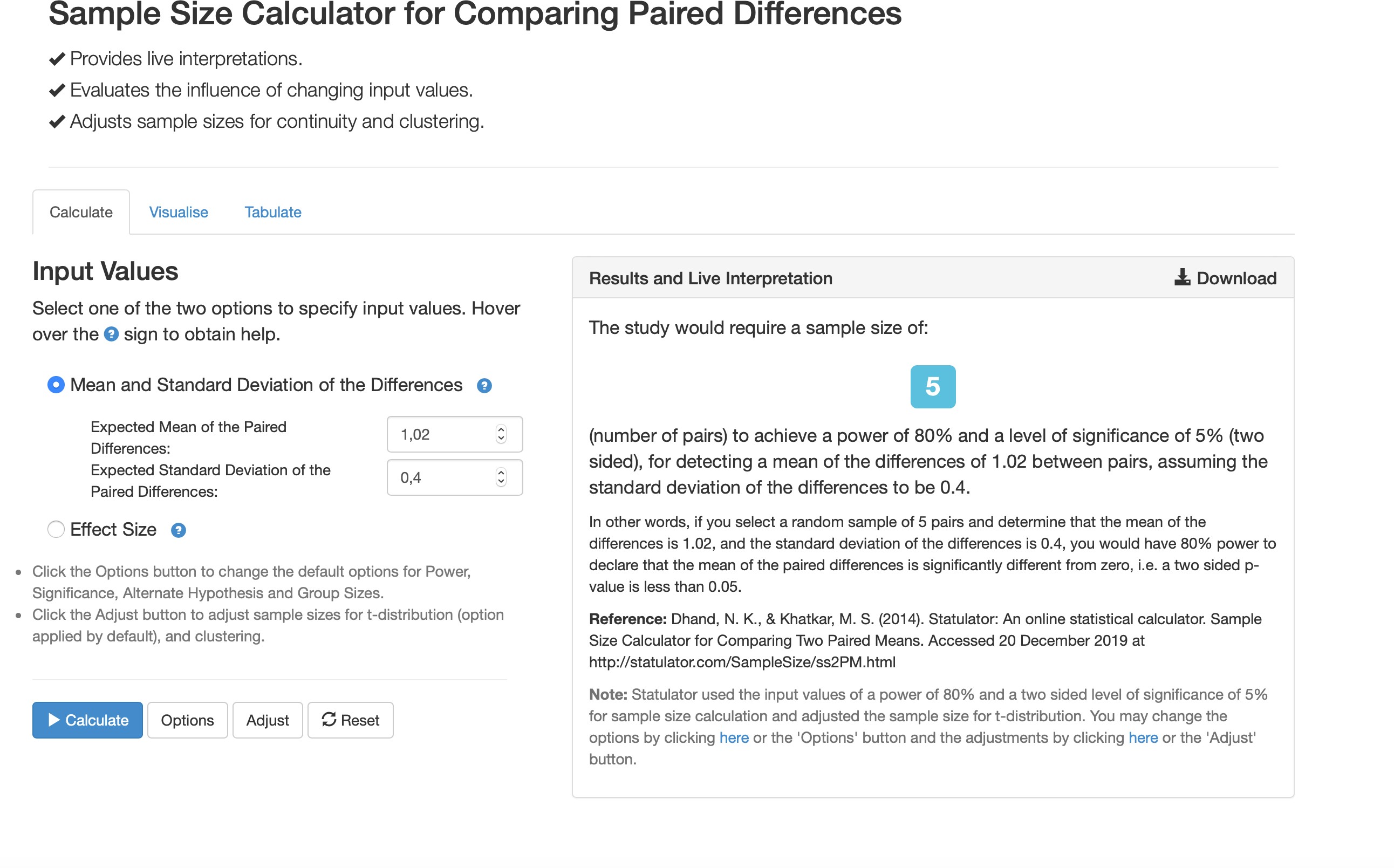

Supplement: Supplementary file 2. [file elife-54523-supp2.docx]
