# Supplementary material for: Increase of circulating IGFBP-4 following genotoxic stress and its implication for senescence
Source: eLife. 2020 Mar 30;9:e54523. doi: 10.7554/eLife.54523 (PMC7136022; doi:10.7554/eLife.54523)
Supplement: Supplementary file 3. [file elife-54523-supp3.docx]

**Primers used for RT-PCR**

| mRNA | sense primer | antisense primer | Optimal  Annealing | Product lenght |
| --- | --- | --- | --- | --- |
| EP1 | ACTTCTAAGCACAACCAG | TTTATTCCCAAAGGCTCT | 59 | 88 |
| EP2 | CCTCCTGTTCTGAGACTAA | TTACTGGCATCTGACTGT | 59 | 104 |
| EP3 | CTTCAATCAGACATCAGT | TCTTAACAGCAGGTAAAC | 57 | 133 |
| EP4 | GTGCTCATCTGCTCCATC | TGATATAACTGGTTGACGAATACT | 58 | 114 |
| GNAS (Gαs) | GCAAGTGGATCCAGTGCTTC | CGGTTGGTCTGGTTGTCCTC | 59 | 100 |
| GAPDH | GGAGTCAACGGATTTGGTCGT | ACGGTGCCATGGAATTTGC | 59 | 161 |
|  |  |  |  |  |
|  |  |  |  |  |
|  |  |  |  |  |
|  |  |  |  |  |
|  |  |  |  |  |
|  |  |  |  |  |
